# Non-invasive optoacoustic imaging of glycogen-storage and muscle degeneration in late-onset Pompe disease

**DOI:** 10.1038/s41467-024-52143-6

**Published:** 2024-09-08

**Authors:** Lina Tan, Jana Zschüntzsch, Stefanie Meyer, Alica Stobbe, Hannah Bruex, Adrian P. Regensburger, Merle Claßen, Frauke Alves, Jörg Jüngert, Ulrich Rother, Yi Li, Vera Danko, Werner Lang, Matthias Türk, Sandy Schmidt, Matthias Vorgerd, Lara Schlaffke, Joachim Woelfle, Andreas Hahn, Alexander Mensch, Martin Winterholler, Regina Trollmann, Rafael Heiß, Alexandra L. Wagner, Roman Raming, Ferdinand Knieling

**Affiliations:** 1 Department of Pediatrics and Adolescent Medicine, University Hospital Erlangen, Friedrich-Alexander-Universität (FAU) Erlangen-Nürnberg, Erlangen, 91054 Germany; 2 Translational Pediatrics, Department of Pediatrics and Adolescent Medicine, University Hospital Erlangen, Friedrich-Alexander-Universität (FAU) Erlangen-Nürnberg, Erlangen, 91054 Germany; 3 Neuromuscular Disease Research, Clinic for Neurology, University Medical Center Göttingen (UMG), Göttingen, 37075 Germany; 4 Translational Molecular Imaging, Max-Planck Institute for Multidisciplinary Sciences (MPI-NAT), City Campus, Göttingen, 37075 Germany; 5 Clinic for Haematology and Medical Oncology, Institute of Diagnostic and Interventional Radiology, University Medical Center Göttingen (UMG), Göttingen, 37075 Germany; 6 Department of Vascular Surgery, University Hospital Erlangen, Friedrich-Alexander-Universität (FAU) Erlangen-Nürnberg, Erlangen, 91054 Germany; 7 Department of Neurology, University Hospital Erlangen, Friedrich-Alexander-Universität (FAU) Erlangen-Nürnberg, Erlangen, 91054 Germany; 8 Institute of Radiology, University Hospital Erlangen, Friedrich-Alexander-Universität (FAU) Erlangen-Nürnberg, Erlangen, 91054 Germany; 9 Department of Neurology, BG-University Hospital Bergmannsheil, Ruhr-University Bochum, 44789 Bochum, Germany; 10 Heimer Institute for Muscle Research, BG-University Hospital Bergmannsheil, 44789 Bochum, Germany; 11 Department of Child Neurology, Justus-Liebig-Universität Giessen, 35385 Giessen, Germany; 12 Department of Neurology, Martin-Luther-Universität Halle-Wittenberg, 06120 Halle (Saale), Germany; 13 Sana Krankenhaus Rummelsberg, 90489 Nuremberg/Schwarzenbruck, Germany; 14 Center for Social Pediatrics, University Hospital Erlangen: Friedrich-Alexander-Universität (FAU) Erlangen-Nürnberg, Erlangen, 91054 Germany; 15 Department of Pediatric Neurology, Center for Chronically Sick Children, Charité Berlin, 13353 Berlin, Germany

**Keywords:** Translational research, Diagnostic markers, Neuromuscular disease

## Abstract

Pompe disease (PD) is a rare autosomal recessive glycogen storage disorder that causes proximal muscle weakness and loss of respiratory function. While enzyme replacement therapy (ERT) is the only effective treatment, biomarkers for disease monitoring are scarce. Following ex vivo biomarker validation in phantom studies, we apply multispectral optoacoustic tomography (MSOT), a laser- and ultrasound-based non-invasive imaging approach, in a clinical trial (NCT05083806) to image the biceps muscles of 10 late-onset PD (LOPD) patients and 10 matched healthy controls. MSOT is compared with muscle magnetic resonance imaging (MRI), ultrasound, spirometry, muscle testing and quality of life scores. Next, results are validated in an independent LOPD patient cohort from a second clinical site. Our study demonstrates that MSOT enables imaging of subcellular disease pathology with increases in glycogen/water, collagen and lipid signals, providing higher sensitivity in detecting muscle degeneration than current methods. This translational approach suggests implementation in the complex care of these rare disease patients.

## Abstract (web summary)

Pompe disease (PD) is a rare disorder that leads to progressive muscle weakness if left untreated. Here, the authors use multispectral optoacoustic tomography (MSOT) to map and quantify the composition of affected muscle tissue to determine disease severity and potentially monitor future therapies.

## Introduction

Pompe disease (PD) is a rare, autosomal-recessive metabolic myopathy caused by mutations in the gene that encodes for acid alpha-glucosidase (GAA)^1–3^. Regularly, GAA catalyzes the hydrolysis of glycogen to glucose, but in PD, its impaired activity results in a generalized build-up of glycogen in metabolic active organs, such as heart, muscle and liver^4,5^. The disease progress is variable in age of onset, severity of organ involvement and degree of myopathy^6^. There is a differentiation in infantile (IOPD) and late-onset (LOPD) forms based on cardiac involvement, age of onset and residual enzyme activity^7^. IOPD patients may have less than 1% GAA activity, therefore, quickly develop severe symptoms, such as cardiac involvement, resulting in a high mortality rate by year one if untreated^1,8^. Children and adults with LOPD have residual enzyme activity below 30%, leading to more slowly progressive limb-girdle type weakness and respiratory insufficiency^9,10^. Replacement therapies (ERT) are available, leading to a slower progression of cardiac and musculoskeletal involvement, prevention of deterioration of pulmonary function and increasing survival^11–14^. However, an early initiation of treatment may positively impact the overall treatment response^15^.

The diagnosis of PD is usually established by confirmation of GAA deficiency, and confirmed by genetic testing^16,17^. Furthermore, PD patients require regular clinical follow-up monitoring, especially to assess the response to ERT^8,9,17–20^. While rapid determination of GAA in dried blood spots is possible, enzymatic analysis is unable to discriminate between patients with PD and those individuals harboring pseudo deficiency mutations. In this regard, a tetraglucose oligomer (Glc(4)) in the urine and maltotetraose (Hex4) in plasma may hold promise as a biomarker to identify PD patients from individuals harboring pseudo deficiency mutations^21^ and even to assess response to ERT^22,23^. However, the interpretation of the values is not trivial and must be considered with respect to the individual age of the patient^18^. Therefore, follow-up is mostly ensured by clinical and functional tests, which are essentially dependent on the individual patient’s active cooperation and performance^1^. More recently, magnetic resonance imaging (MRI) studies in LOPD demonstrated significant correlations between muscle involvement and function^24–26^ or efficacy of enzyme replacement therapy and degree of lipomatous muscle alterations^27^. Moreover, it clearly identifies the proximal to distal involvement pattern of the disease^25^. Particularly in young patients, MRI has attributable risks for the requirement of sedation or difficult positioning for patients with respiratory impairment. Therefore, there is an unmet need for non-invasive techniques to better and more objectively assess disease involvement directly in the muscles of PD patients with the lowest burden possible. In this regard, multispectral optoacoustic tomography (MSOT) may be used to quantitatively image subcellular tissue composition and visualize disease-specific muscle changes^28–31^. MSOT applies the principle of “light in and sound out” through short-pulsed near-infrared laser emission and ultrasound detection, enabling it to retrieve deep-tissue information^32–37^. The mechanism of thermal expansion-based optoacoustic imaging (OAI) is that optical energy is absorbed by tissue chromophores, such as hemoglobins, lipids, water or collagens, causing localized heating and expansion, generating detectable acoustic pressure waves^38^. In this work, we show that in LOPD patients, MSOT enables imaging of subcellular disease pathology with increases in glycogen/water, collagen and lipid signals, providing higher sensitivity to detect muscle degeneration than current methods.

## Results

### Phantom imaging reveals optoacoustic properties of glycogen

**Fig. 1:**
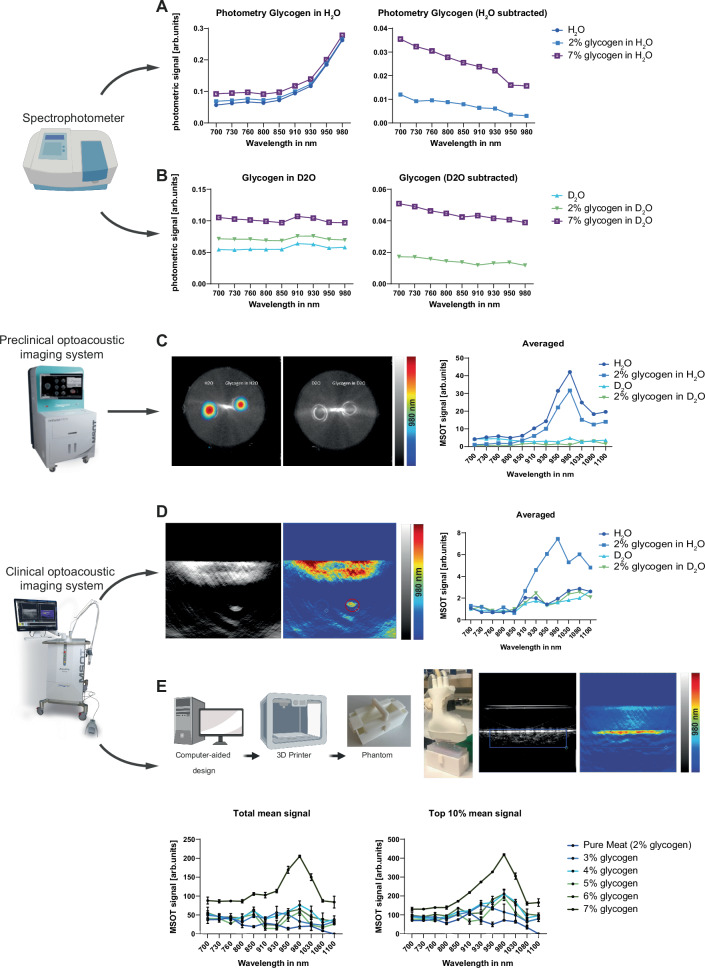
Multimodal derivation of spectral information for glycogen reveals specific signatures for clinical imaging. **A** Photometric absorption spectra between 700 and 980 nm for pure H_2_O, and H_2_O with 2% and 7% glycogen, respectively (left). Photometric absorption spectra with subtracted H_2_O background (right). **B** Photometric absorption spectra between 700 and 980 nm for pure D_2_O, and D_2_O with 2% and 7% glycogen, respectively (left). Photometric absorption spectra with subtracted D_2_O background (right). **C** Averaged optoacoustic signal in the preclinical imaging system from 700 to 1100 nm for pure H_2_O, pure D_2_O, and 2% glycogen in H_2_O and D_2_O, respectively. **D** Averaged optoacoustic signal in the clinical imaging system from 700 to 1100 nm for pure H_2_O, pure D_2_O, and 2% glycogen in H_2_O and D_2_O, respectively. **E** Averaged optoacoustic signal in the clinical imaging system from 700 to 1100 nm for pure minced meat and minced meat of the same origin with increasing glycogen concentrations. Values are given as mean values of scan data with negative signal intensities set to 0 or given as mean values of the top 10% of signal intensities per scan. The data represent one of two independent experiments with similar results. This figure was created with BioRender.com released under a Creative Commons Attribution-NonCommercial-NoDerivs 4.0 International license.

To identify a possible specific glycogen spectrum, we first aimed to determine whether we could visualize its spectrophotometric absorption. An increase in glycogen concentration did not change the specific peak of the photometric spectrum with the investigated wavelengths (Fig. 1A). We could observe an increase of absorption starting from 910 nm, as described in the literature for H_2_O^39^. After subtracting H_2_O background, there is a flat spectrum curve remaining, with only an absolute absorption shift between 2% and 7% glycogen (Fig. 1B). In contrast, D_2_O does not show an increase of absorption within the observed wavelengths (Fig. 1C) and after subtraction of D_2_O background it showed similar curves as compared to H_2_O experiments (Fig. 1D), which indicates no specific spectrophotometric absorption of glycogen.

To examine the optoacoustic properties of glycogen, we used a preclinical imaging system designed for small animal imaging^40^. Using this setup, H_2_O and 2% glycogen in H_2_O were detected, while D_2_O and 2% glycogen in D_2_O had no specific signal increase (Fig. 1E). Next, we transferred this to a clinical imaging system^28,41^, which detected higher signal values when glycogen was added into H_2_O, especially starting at 910 nm and a pronounced peak at 980 nm (Fig. 1F). The H_2_O, D_2_O and glycogen in D_2_O showed similar curve progression. To validate the transferability of our in vitro glycogen findings into an actual muscle, we developed an ex vivo muscle phantom. Using a 3D-printed mold filled with minced meat, we started diluting it in H_2_O and successively added glycogen. We increased the glycogen content in 50% steps in relation to the calculated concentration in pure meat and imaged it using the clinical system. Our comparison shows an increasingly higher signal over several single wavelengths (SWLs), including 830 nm, 850 nm, 980 nm, 1030 nm, and 1080 nm for increasing concentrations of glycogen (Fig. 1G, H). This suggests that glycogen, although not detectable as a pure substance, offers an optoacoustic imaging target in the clinical setting due to its potentially high-water binding capacity.

### Implementation of a clinical trial to study MSOT imaging

**Fig. 2:**
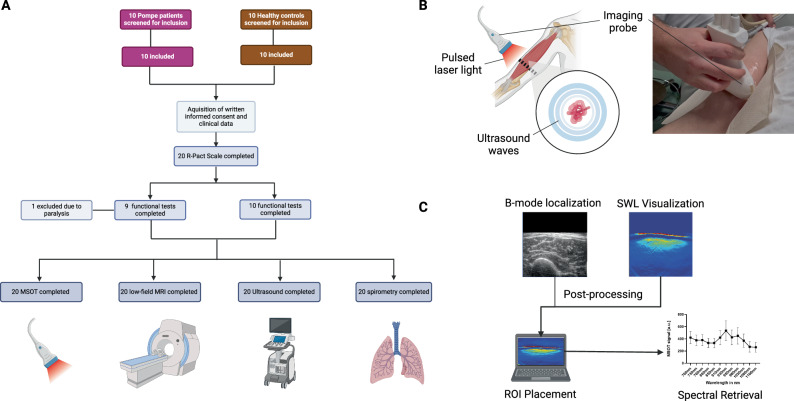
Study flowchart imaging approach and quantification of scan results. **A** Consort flowchart diagram of the study. **B** Schematic and photographic representation of MSOT imaging approach. The imaging probe emitting pulsed laser light was held onto the distal third of the upper arm, scanning the biceps muscle. **C** Localization of appropriate scan was performed on ultrasound B-mode images. These were used to post-process optoacoustic spectral information. MSOT multispectral optoacoustic tomography, MRI magnetic resonance imaging, ROI region of interest, R-Pact Rasch-built Pompe-specific activity score. This figure was created with BioRender.com released under a Creative Commons Attribution-NonCommercial-NoDerivs 4.0 International license.

As reported previously, MSOT was capable of characterizing muscular remodeling with high sensitivity in neuromuscular diseases^28,30,42^. Given our in vitro findings in glycogen models, we hypothesized that it is possible to rapidly quantify LOPD-specific muscle involvement already in less affected proximal muscle groups. To perform an in vivo study of the MSOT imaging approach, we implemented a clinical trial. After regulatory approval and prospective registration to the clinical trial register, we included a total *n* = 10 healthy volunteers (HV), which were gender and age-matched to *n* = 10 LOPD patients (Fig. 2A). Besides clinical standard assessment, all patients were imaged using MSOT (Fig. 2B) and the resulting data was processed (Fig. 2C). The mean age ± SD was 41.2 ± 14.2 years in HV compared to 40.6 ± 12.1 years in the LOPD patients’ cohort. In each group, 5 [50%] subjects were females. Eight (80%) of the LOPD patients received enzyme replacement therapy (ERT) with Alglucosidase alpha (Myozyme®, Sanofi). Four (40%) of LOPD patients needed nocturnal ventilation support. To determine the degree of disease, all HV completed the Rasch-built Pompe-specific activity scale (R-Pact), Quick Motor Function Test (QMFT), Medical Research Council (MRC), Up and Go test (TUG). 9 HV completed 6-Min Walk test (6-MWT) (one patient was not able to perform 6-MWT). 10 LOPD patients performed the R-Pact, QMFT and MRC, 9 LOPD patients performed TUG and 6-MWT (one patient was not able to perform 6-MWT and TUG due to his disease progression). Overall scores were significantly lower in LOPD patients (matched *n* = 10 HV vs*. n* = 10 LOPD patients: R-Pact: 36.0 ± 0.0 vs. 29.3 ± 10.1, *P* = 0.0156; QMFT: 64.0 ± 0.0 vs. 46.7 ± 16.1, *P* = 0.0078; MRC: 180.0 ± 0.0 vs. 149.5 ± 35.3, *P* = 0.0078; TUG: 1.7 ± 0.4 s vs. 3.7 ± 3.0 s, *P* = 0.1421; 6-MWT: 671.3 ± 116.6 m vs. 560.3 ± 87.2 m, *P* = 0.0736). The standard assessments are given in Table 1.

**Table 1:** Characteristics of LOPD patients and healthy volunteers

	HV *n* = 10	LOPD *n* = 10
Female, (%)	5 (50)	5 (50)
Male (%)	5 (50)	5 (50)
Age, years	41.2 ± 14.2	40.6 ± 12.1
Weight, kg	73.9 ± 11.9	65.0 ± 17.3
Height, cm	176.2 ± 0.1	172.3 ± 0.1
BMI (kg/m^2^)	23.6 ± 2.2	21.6 ± 4.0
Ambulatory (%)	10 (100)	9 (90)
ERT (%)	0 (0)	8 (80)
Nocturnal ventilation support	0 (0)	4 (40)
**Lung function**		
FEV1 (%)	100.8 ± 11.8	68.2 ± 23.5
FVC (%)	104.9 ± 9.7	62.6 ± 21.4
**Functional testing**		
R-PACT	36.0 ± 0.0	29.1 ± 10.4
QMFT	64 ± 0.0	46.7 ± 16.1
6MWT (m)	671.3 ± 116.6	560.3 ± 87.2
TUAG (s)	1.7 ± 0.4	3.7 ± 3.0
MRC		
UB		
Proximal	5.0 ± 0.0	4.3 ± 0.1
Medial	5.0 ± 0.0	4.5 ± 0.1
Distal	5.0 ± 0.0	4.7 ± 0.0
LW		
Proximal	5.0 ± 0.0	3.9 ± 0.1
Medial	5.0 ± 0.0	4.1 ± 0.1
Distal	5.0 ± 0.0	4.5 ± 0.1

Values are mean ± standard deviation (SD).

*LOPD* late-onset Pompe disease, *HV* healthy volunteer, *BMI* body mass index, *ERT* enzyme replacement therapy, *FEV1* forced expiratory volume, *FVC* functional vital capacity, *R-Pact* Rasch-built Pompe-specific activity score, *QMFT* quick motor function test, *6MWT* 6-min walking test, *TUAG* timed up-and-go test, *MRC* Medical Research Council score, *UB* upper body, *LB* lower body, *SD* standard deviation.

### Standard ultrasound and magnetic resonance imaging in LOPD

**Fig. 3:**
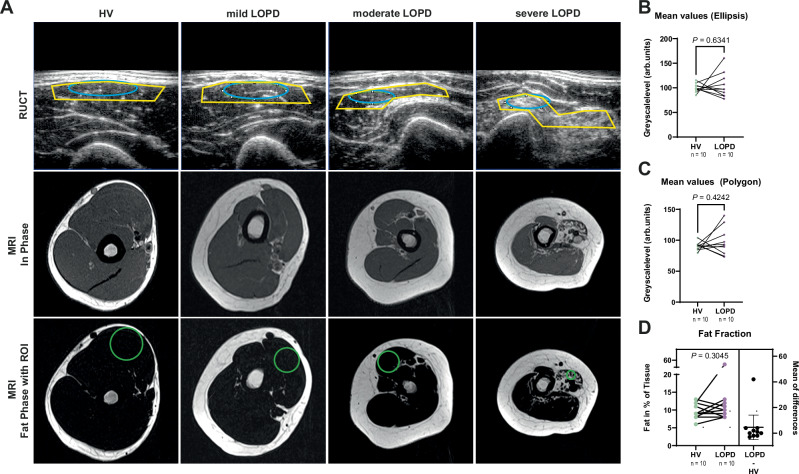
Standard muscle imaging by ultrasound and magnetic resonance imaging does not show discernable differences in biceps muscles of LOPD patients. **A** Ultrasound images (top row) and In-Phase (middle row) and Fat-Phase (bottom-row) MRI of the biceps muscle. From left to right, HV, mildly, moderately and severely affected LOPD patients. Elliptic (blue) and polygonal (yellow) ROI used in RUCT images and circular ROI used in Fat-Phase MRI for quantification. **B** Mean GSL values of matched HV vs. LOPD patients using an elliptic ROI. **C** Mean GSL values of matched HV vs. LOPD patients using a polygonal ROI. Each independent muscle region was scanned twice. Results represent 80 datasets from *n* = 40 independent biceps muscle regions (*n* = 20 HV/*n* = 20 LOPD) in *n* = 20 biologically independent subjects (*n* = 10 HV and *n* = 10 patients with LOPD). Each filled dot represents one MSOT signal per mean biceps muscle region (4 datasets from *n* = 2 independent independent muscle regions from one biologically independent subject). HV are represented with green and LOPD patients with violet dots. **D** ROIs in MRI images were manually placed in transversal slices of the right biceps brachii muscle corresponding to the position of MSOT evaluation. Results represent 20 datasets from *n* = 20 independent biceps muscle regions (*n* = 10 HV/*n* = 10 LOPD) in *n* = 20 biologically independent subjects (*n* = 10 HV and *n* = 10 patients with LOPD). Each filled circle represents the fat fraction in percent per tissue signal per mean right biceps muscle (1 dataset from *n* = 1 independent muscle region from one biologically independent subject). HV are represented with green and LOPD patients with violet dots. To display differences in fat fractions, mean values from HV were subtracted from LOPD patients. One black dot represents one calculated ratio. Confidence intervals represent 95% CI ranging from −4.957 to 14.16, effect size (*R*^2^) 0.1164, mean of differences (LOPD – HV) 4.6, SD of differences 13.36, SEM of differences 4.225. Two-tailed dependent samples *t*-tests (matched for age and sex) were used for statistical analysis. If the assumption of normal distribution was violated, a Wilcoxon signed-rank was used. *P* ≤ 0.05 was considered statistically significant. HV healthy volunteer, LOPD late-onset Pompe disease patient, ROI region of interest, RUCT reflected ultrasound computed tomography, MRI magnetic resonance imaging.

Next, we investigated whether B-Mode ultrasound (US) or magnetic resonance imaging (MRI) was capable of determining biceps muscle involvement in PD (Fig. 3A). For US, a total of *n* = 40 independent scans of the biceps muscle of HV (*n* = 20) and LOPD patients (*n* = 20) were evaluated. In HV, all 20 (100%) muscles were rated normal by an experienced and certified clinical investigator. Given the variability of the clinical phenotype, blinding was not fully feasible for all patients. By comparison, only 13 (65%) of the LOPD patients’ biceps scans were rated normal, with 7 (35%) showing an overall pathological rating (Table 2).

**Table 2:** B-mode ultrasound results of biceps muscle regions

	Ultrasound scoring	HV (*n* = 10 individuals, *n* = 20 scans)	LOPD (*n* = 10 individuals, *n* = 20 scans)
Echogenicity	Hypo-echogenic	16 (80%)	7 (35%)
	Isoechogenic	4 (20%)	4 (20%)
	Hyperechogenic	0	9 (45%)
Muscle texture	Coarse-granular	3 (15%)	1 (5%)
	Medium-granular	3 (15%)	4 (20%)
	Fine-granular	14 (70%)	15 (75%)
Distribution pattern	Focal	0	1 (5%)
	Inhomogeneous	4 (20%)	6 (30%)
	Homogeneous	16 (80%)	13 (65%)
Heckmatt scale	1	20 (100%)	18 (90%)
	2	0	2 (10%)
	3	0	0
	4	0	0
Pathological	No	20 (100%)	13 (65%)
	Yes	0	7 (35%)

*N* = 40 images (*n* = 20 HV/*n* = 20 LOPD) were evaluated for echo intensity, muscle texture, distribution pattern, Heckmatt scale and pathological rating. The investigator (J.J.) assessed echogenicity (hypo-/iso-/hyperechogenic), muscle texture (coarse-/medium-/fine-granular), distribution pattern (focal/inhomogeneous/homogeneous) and Heckmatt scale (grade 1–4: 1 = normal muscle echo, 2 = increased muscle echo while bone is still distinct, 3 = increased muscle echo and reduced bone echo, 4 = very strong muscle echo and loss of bone echo) in parallel to the examination. Additionally, the muscle was evaluated by the overall impression as healthy or pathological. Categorial variables are provided as numbers and percentages. *N* = 40 independent biceps scans (*n* = 20 HV/*n* = 20 LOPD) in *n* = 20 biologically independent subjects (*n* = 10 HV/*n* = 10 LOPD).

*HV* healthy volunteers, *LOPD* late-onset Pompe disease.

Next, a comparison of the greyscale level (GSL) of the muscles between the diseased and the healthy subjects was performed^43^. The values were retrieved for two different regions of interest. One ellipsoidal in the center of the muscle and one polygonal ROI outline the major proximal portion of the muscle. The total mean value per patient showed no significant difference both in elliptic (84.0 ± 9.5 arb.units vs. 93.4 ± 13.1 arb.units, *P* = 0.17, Fig. 3B) and polygonal ROIs (80.6 ± 7.8 arb.units vs. 90.2 ± 11.8 arb.units, *P* = 0.1, Fig. 3C).

As MRI fat fraction is supposedly one of the most sensitive methods to detect muscle involvement, all subjects underwent an MRI of the biceps muscle. Signal intensities of in-phase and fat-only images were used to estimate the fat fraction (FF) as a surrogate marker for muscle atrophy in accordance with the pathophysiology of PD. In total, *n* = 20 datasets of the respective right biceps of HV and LOPD patients were evaluated. Overall, no difference in the fat fraction as surrogate for muscle involvement was found between HV and LOPD patients (9.7 ± 2.2% vs. 14.3 ± 14.1%, *P* = 0.38, Fig. 3D). These findings are in accordance with clinical phenotype and previous studies showing a less pronounced muscle involvement in LOPD^25^.

### MSOT enables visualization of biceps muscle involvement

**Fig. 4:**
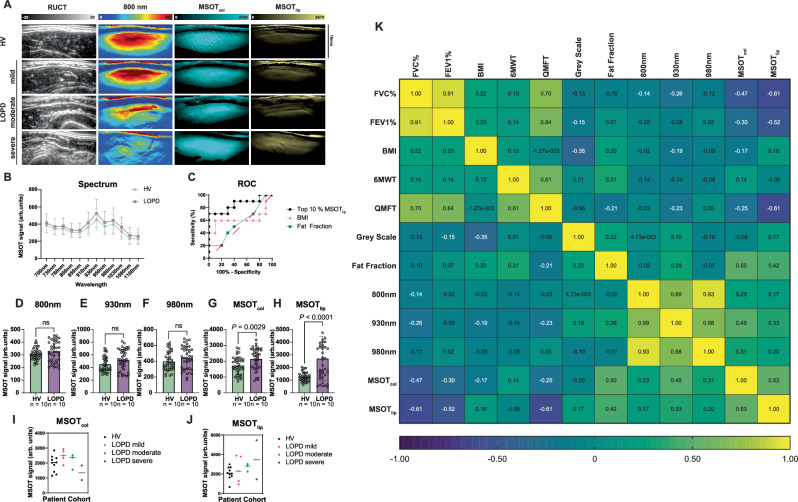
MSOT quantification in human biceps muscles. **A** From left to right: representative MSOT imaging quantification representing anatomic information (RUCT), unspecific tissue/muscle signal (SWL 800 nm), MSOT_col_ and MSOT_lip_. Disease severity of HV vs. LOPD (mildly, moderately and severely) is increasing from top to bottom cases. **B** Comparison of MSOT spectral signal values of HV and LOPD patients from 700 to 1100 nm. Each dot represents the mean of a whole proband group (HV = green, LOPD = violet), bars represent 95% CI. Results represent 80 datasets from *n* = 40 independent biceps muscle regions (*n* = 20 HV/*n* = 20 LOPD) in *n* = 20 biologically independent subjects (*n* = 10 HV and *n* = 10 patients with LOPD). **C** ROC Curve of Top 10% signals MSOT_lip_, BMI values and MRI fat fraction values to distinguish HV and LOPD muscles. *n* = 40 independent muscle regions (*n* = 20 HV/*n* = 20 LOPD) in *n* = 20 biologically independent subjects (*n* = 10 HV and *n* = 10 LOPD). Comparison of Top 10% of signal intensity for SWL 800 nm (**D**), 930 nm (**E**), 980 nm (**F**), MSOT_col_ (**G**), MSOT_lip_ (**H**) between HV and LOPD patients with individual scans as individual data points. Results represent 80 datasets from *n* = 40 independent biceps muscle regions (*n* = 20 HV/*n* = 20 LOPD) in *n* = 20 biologically independent subjects (*n* = 10 HV and *n* = 10 patients with LOPD). Each bar displays the mean of top 10% MSOT signal of the biceps muscle of a whole proband group with the error bars indicating SD (green bar/dots = HV and violet bar/dots = LOPD). MSOT signal comparison for different LOPD severity (HV = black, mild = pink, green = moderate, severe = purple) for MSOT_col_ (**I**) and MSOT_lip_ (**J**). Results represent 80 datasets from *n* = 40 independent biceps muscle regions (*n* = 20 HV/*n* = 20 LOPD) in *n* = 20 biologically independent subjects (*n* = 10 HV and *n* = 10 patients with LOPD). Each filled dot shows the mean of top 10% signal of the biceps muscle of different severity groups and HV (black dots = HV = QMFT = 64, pink dot = mild LOPD = QMFT 64–49, green dot = moderate LOPD = 48–33, purple dot = severe PD = 32–0). Statistical difference was tested with Welch’s *t*-test. **K** Correlation matrix for maximum MSOT signal intensity of SWL 800 nm, 930 nm, 980 nm and MSOT_col_ and MSOT_lip_ correlated to reference clinical parameters including FVC%, FEV1%, BMI, 6MWT, QFMT, ultrasound greyscale levels, fat fraction. Correlations are indicated in the color range from highly negative (blue) to low negative/positive (green) to highly positive (yellow). Correlations are given by Spearman correlation coefficient (rs), two-tailed test. *P* ≤ 0.05 was considered statistically significant. *n* = 20 biologically independent subjects (*n* = 10 HV/*n* = Confidence interval was 95% 10 patients with LOPD). HV healthy volunteer, LOPD late-onset Pompe disease patient, MSOT multispectral optoacoustic tomography, RUCT reflected ultrasound computed tomography, ROC receiver operating characteristic curve, FVC functional vital capacity, FEV1 forced expiratory volume, BMI body mass index, 6MWT 6-min walking test, QMFT quick motor function test.

For all subjects, MSOT imaging was completed, and data was post-processed using two independent scans of each biceps muscle for the final analysis. In total, *n* = 80 scans (*n* = 40 of HV, *n* = 40 of LOPD) were included, and signals, both SWLs and MSOT parameters (MSOT-derived lipid signal, MSOT_lip_; MSOT-derived collagen signal, MSOT_col_), were compared between groups. Figure 4A shows exemplary imaging results. Given the heterogeneity of the disease manifestation, each muscle was regarded as an individual data point. Therefore, *n* = 20 matched muscle regions were compared. The optoacoustic spectrum derived from 12 SWL showed overall higher values for PD patients compared to HV (Fig. 4B). MSOT_lip_ provided better performance to distinguish HV from LOPD than clinical parameters (body mass index, BMI) or fat fraction derived by MRI (Fig. 4C). Spectral unmixing derived higher MSOT_col_ (1727 ± 555.5 arb.units vs. 2152 ± 674.0 arb.units, *P* = 0.0029), MSOT_lip_ signals (1267 ± 356.6 arb.units vs. 2713 ± 1732 arb.units, *P* < 0.0001), while in this approach SWL (800, 930, 980 nm) signals remained unchanged (Fig. 4D–H). However, the exact measure also depends on the proportion of the signal that is quantified (Supplementary Figs. 1–4). By separating patients with regard to disease severity based on QMFT, one can observe decrease of MSOT_col_ and increasing MSOT_lip_ quantification, possibly resembling the fibro-fatty degeneration of the muscle (Fig. 4I, J). MSOT_lip_ demonstrated the strongest correlation to the other investigated clinical standard assessment (Fig. 4E).

### Proofing applicability and validity of MSOT using multicenter data

**Fig. 5:**
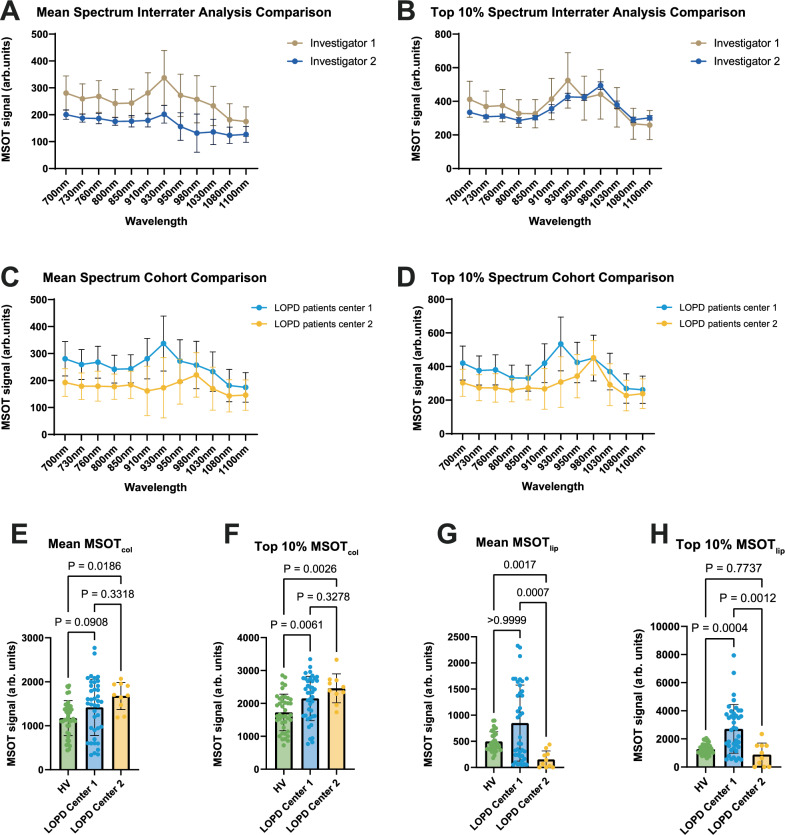
Multicenter patient cohort comparison proves the applicability and validity of MSOT approach. **A** Interrater analysis comparison of MSOT mean signals for two independent investigators (investigator 1, investigator 2) of two clinical centers (LOPD center 1, LOPD center 2) independently analyzed the same MSOT biceps muscle scans (10 datasets from *n* = 6 independent biceps muscle regions (*n* = 3 LOPD) in *n* = 3 biologically independent subjects). Data collected by center 2. **B** Interrater analysis comparison of MSOT top 10% signals for two independent investigators (investigator 1, investigator 2) of two clinical centers (LOPD center 1, LOPD center 2) independently analyzed the same MSOT biceps muscle scans (10 datasets from *n* = 6 independent biceps muscle regions (*n* = 3 LOPD) in *n* = 3 biologically independent subject). Data collected by center 2. Dual center comparison (center 1: *n* = 10 vs. center 2: *n* = 3) of mean (**C**) and top 10% (**D**) MSOT signals. Each filled blue circle displays the mean (**C**) and top 10% (**D**) of LOPD patients of center 1, each filled yellow circle displays the mean (**C**) and top 10% (**D**) of LOPD patients of center 2. Results of center 1 represent 80 datasets from *n* = 40 independent biceps muscle regions (*n* = 20 HV/*n* = 20 LOPD) in *n* = 20 biologically independent subjects (*n* = 10 HV and *n* = 10 patients with LOPD). Results of center 2 represent 10 datasets from *n* = 6 independent biceps muscle regions (*n* = 3 LOPD) in *n* = 3 biologically independent subjects. Comparison of mean MSOT_col_ (**E**), top 10% MSOT_col_ (**F**), mean MSOT_lip_ (**G**), and top 10% MSOT_lip_ (**H**) between HV and LOPD patients of both centers. Green bar representing HV consists of 40 datasets from *n* = 20 independent biceps muscle regions of *n* = 10 biologically independent subjects. Blue bar representing LOPD Center 1 of 40 datasets from *n* = 20 independent biceps muscle regions of *n* = 10 biologically independent subjects, yellow bar representing center 2 consists of 10 datasets from *n* = 6 independent biceps muscle regions (*n* = 3 LOPD) of *n* = 3 biologically independent subjects. Ordinary one-way ANOVA was used for statistical analysis. If the assumption of normal distribution was violated, a Kruskal–Wallis test was used. Box plots are defined with a minimum at the 25th percentile, a maximum at the 75th percentile, center at the median value and whiskers at the minimal and maximal data points of each subgroup. MSOT multispectral optoacoustic tomography, HV healthy volunteer, LOPD late-onset Pompe disease patient.

For better inter-device, -center and -examiner comparability, a similar investigation using an identical imaging approach on LOPD patients was conducted at a second center. This center produced *n* = 3 datasets from *n* = 3 individual LOPD patients, including duplicate scans of the right and left biceps muscle using the same imaging device. First, both centers independently analyzed the second center data and retrieved nearly identical results (Fig. 5A, B). Next, we compared the patient cohorts (*n* = 10 vs. *n* = 3 LOPD patients) and compared the spectra (Fig. 5C, D). Most likely these are influenced by heterogeneity of the disease. When using spectral unmixed MSOT_col_, we observed similar increased higher values in LOPD patients in both datasets (Fig. 5E). For MSOT_lip,_ we found larger differences in both centers (Fig. 5E).

### MSOT requires minimal scanning times

The investigation time for MRI depends on several factors, including the specific region of the body, patient-related considerations such as disabilities, patient cooperation, movement artefacts and repetition of imaging sequences. For the right biceps muscle scan time for T1 sequence requires 07:28 min, for T2 sequence 05:28 min. Additional time is needed to localize and optimize the scanning region, resulting in a minimal total scan time of 13:32 min.

In contrast, MSOT exhibits significantly shorter scan times. On average, one MSOT scan takes approximately 10 s. For the purpose of our study, we took two scans per biceps muscle This notable difference in investigation time highlights the advantage of MSOT in clinical use.

## Discussion

The findings presented here provide a rationale for a novel, non-invasive, radiation-free, and easy-applicable imaging modality to visualize disease-specific muscle patterns in LOPD patients. From a clinical perspective, we demonstrate the feasibility of MSOT to detect tissue remodeling caused by glycogen and its related higher water content. In the clinical imaging setup, glycogen in H_2_O has higher MSOT signals and a pronounced peak at 980 nm compared to pure H_2_O. Studying muscle-mimicking phantoms, we found similar optoacoustic signal behavior correlating to the concentration of glycogen. Considering these results, we hypothesize that the current clinical optoacoustic system most likely visualizes glycogen-bound water. Taking this into consideration, we retrieved increased lipid and collagen signals as a sign of muscle degeneration in clinical subjects—also likely affected by the high glycogen-bound water in the muscle tissue. In contrast to our MSOT imaging findings, clinical routine US or MRI techniques were not feasible to visualize similar features in LOPD patients. Moreover, MSOT imaging is easily transferable to a second clinical center providing comparable imaging results. This unpins the utility and potential for MSOT for future non-invasive treatment monitoring in PD patients.

Currently, the first-line treatment in PD (IOPD and LOPD) is enzyme replacement therapy (ERT). In order to increase uptake, it is glycosylated with mannose-6-phosphate (M6P), which binds to cation-independent mannose-6-phosphate receptors (CIMPR) on cell membranes^44–46^. This allows affected tissue cells to take up the exogenous enzyme and increase their capability to degrade glycogen, subsequently clearing the excessive glycogen storages^11,47^. However, the affection of endocytic and autophagic pathways and the development of GAA-antibodies have a significant impact on trafficking and processing of ERT and subsequent therapeutic response^48^. As a consequence, the majority of patients show improvement or stabilization of respiratory and muscular functions, with more prominent effects at earlier stages of disease^49,50^. Laboratory biomarkers, including the assessment of GAA activity in dried blood spots or the analysis of Glc4 and Hex4, are applicable but not widely available or standardized across different centers^21–23^. Common tools to monitor PD patients are function-related, rely on active cooperation, may show a learning effect, and thus may not be sufficiently objective enough^51–53^. Moreover, therapeutic monitoring is currently becoming even more important because, in addition to new ERTs^47,54^, genetic interventions for PD patients are also on the horizon^55,56^. Second, early identification of LOPD patients in newborn screening programs may require more precise phenotyping^57^.

Imaging technologies may offer the advantage of directly assessing changes in muscle structure and composition. The quantification of intramuscular fat or fat fraction (FF) has been shown to provide a solid correlation with muscle function and clinical outcomes in adults^24,25,58–60^. Less consistent results, as demonstrated in our study, facilitated the development of more sophisticated muscle MRI sequences, including diffusion tensor imaging^26^ or glycogen spectroscopy^61,62^. Even without the presence of significantly increased fatty infiltration, these techniques can depict structural changes^26^. However, MRI may have several disadvantages, such as high costs, limited availability, lack of (inter-center) protocol standardization, and long examination time coupled with strict positioning of the patient. With progress in standardization and further clinical translation^63,64^ as shown in this study, MSOT may enable easy-applicable imaging phenotyping of PD patients. The capability of MSOT has already been explored in gastroenterology^41,65–68^, rheumatology^69^, cardiovascular^70^ and cancer medicine^71^ or more specifically in muscle applications^42^ such as Duchenne muscular dystrophy^28^ and spinal muscular atrophy^30^. In this study, we assume that we were able to depict and quantify the muscle remodeling with localized glycogen accumulation, subsequently leading to fibro-fatty replacement and muscle atrophy. The spectral unmixed parameters, MSOT_lip_ and MSOT_col_, support our hypothesis that MSOT is capable of detecting such processes in the affected muscle.

In conclusion, MSOT holds great potential to become a sensitive imaging technology for diagnosing, phenotyping and monitoring patients with LOPD. With the increasing importance and availability of gene therapy, early treatment is vital. The findings of this work suggest the implementation of MSOT imaging into the comprehensive and complex care of these rare disease patients and could possibly reduce other more invasive procedures in the future. The next step should be a follow-up study including pediatric patients (both with IOPD and LOPD).

Nevertheless, this study has several limitations to consider. It is constrained by a small and heterogeneous sample size, which is attributed to the rarity of PD patients and the diversity in its clinical manifestations. Furthermore, MSOT is influenced by optical absorbers such as melanin, making it suitable primarily for individuals with lighter skin color and holds a maximum penetration depth of approximately 2.5 cm restricting MSOT application in certain cases and regions of the body. Further work is needed to develop a PD-specific methodology to evaluate disease-specific muscle involvement at different stages of the disease that relate to clinical findings. Moreover, optoacoustic imaging systems are still a novel imaging approach that have to be further improved and standardized^64^. Unmixing algorithms, reconstruction technique and analysis have to be enhanced to improve the quality of data^64,72^.

## Methods

### Study design and subjects

A prospective, monocentric clinical study was conducted after receiving approval from the local ethics committee of the University Hospital Erlangen (UHE), Germany (reference: 21-238_1-B) and registration at clinicaltrials.gov (ID NCT05083806). This trial was performed according to the Declaration of Helsinki, and all subjects provided written informed consent. All investigations were performed at a single visit per participant. Inclusion criteria were confirmed Pompe disease (PD) independent from current therapy with an age over 18 years. Exclusion criteria were pregnancy, tattoo in skin area to be examined, contraindications for MSOT, MRI and for healthy volunteers (HV) any signs of myopathy. All participants were investigated between May 17, 2022, and March 30, 2023. A compensation of 300 Euro/participant was granted. The gender of LOPD patients and their matched controls was self-reported. The mean age ± SD was 41.2 ± 14.2 years in HV compared to 40.6 ± 12.1 years in the LOPD patients’ cohort. In each group, 5 [50%] subjects were self-reported females.

For the inter-device, -center and -examiner comparison, the second site received an approval from the local ethics committee of the University Medical Center Göttingen (UMG), Germany (reference2/7/22). The inclusion and exclusion criteria followed the original approval of the University Hospital Erlangen.

### Phantom development and imaging

#### Spectrophotometer imaging

Absorption spectrum was measured every 10 nm in the range of 680–980 nm using the spectrophotometer SpectraMaxM2e (Molecular Devises, San Jose, USA). Six samples (H_2_O, 2% glycogen in H_2_O, 7% glycogen in H_2_O, D_2_O, 2% glycogen in D_2_O, and 7% glycogen in D_2_O) were measured separately.

#### In vitro phantom imaging studies

For in vitro imaging experiments, a phantom was custom-built by dissolving 2% agarose (Biozym LE Agarose, Biozym Scientific GmbH, Hessisch Oldendorf, Germany) in heated D_2_O (Euriso-Top, St-Aubin Cedex, France). The warm fluid was filled into a cylindrical mold leaving two wells, each of 3 mm in diameter. After cooling down, the phantom solidified, leaving two separate holes within the phantom. Four samples: H_2_O, D_2_O, 2% glycogen (from bovine liver ≥85%, Sigma-Aldrich, St Louis, USA) in H_2_O, and 2% glycogen in D_2_O were each mixed with 1.5% agarose at 37 °C. Each phantom was filled with two different samples, either with H_2_O and glycogen in H_2_O or D_2_O and glycogen in D_2_O. Before injecting the warm fluid into the phantom holes, the phantom was placed on ice. This way, the warm and fluid samples cooled down and solidified quickly as they were injected. Next, imaging was performed from 660 nm to 1300 nm in 5 nm steps using a preclinical MSOT inVision Echo system (iThera Scientific, Munich, Germany). This system is also capable of acquiring interleaved US images for the coregistration of imaging data. Data was analyzed using viewMSOT software (version 4.1, iThera Medical GmbH, Munich, Germany). A region of interest (ROI) was drawn around the sample inclusions using the US image for local guidance. Mean signal intensity in the ROI at each wavelength was quantified and then plotted into an optoacoustic spectrum. We transferred this setup to a clinical MSOT Acuity Echo system (iThera Medical, Munich, Germany). An identical phantom was established using the same materials but filled into a box-shaped mold. Thus, we improved the contact area for the handheld probe. The four samples (H_2_O, D_2_O, 2% glycogen in H_2_O, and 2% glycogen in D_2_O) were processed and injected according to the steps above. Thereafter, the phantom was imaged from 660 nm to 1300 nm in 10 nm steps. The same examiner analyzed the data and drew the ROI as described above.

#### Ex vivo muscle mimicking phantom

To develop an ex vivo muscle-mimicking phantom, we used ground beef as a tissue substitute. Similar to the phantom described before, a custom-built phantom was established from 2% agarose (Biozym LE Agarose, Biozym Scientific GmbH, Hessisch Oldendorf, Germany) dissolved in distilled water, which was filled in a custom, 3D-printed mold. The mold was developed using Autodesk Fusion 360 (V2.0.14567, Autodesk GmbH, München, Germany) and a 3D printer (Form 2, Formlabs. Inc, Somerville, MA, USA) utilizing White Resin V4 (Formlabs. Inc, Somerville, MA, USA)^65^. The phantom main body measured 96*46*40 mm, which covered and sealed a recess of 76 × 26 × 20 mm. This remaining recess was subsequently filled with ground beef and glycogen at increasing concentrations. The initial weight of minced meat was 20 g (= 100%), which was diluted in 20 ml of H_2_O. We assumed that the glycogen content of minced meat is equal to 2%, which equals 0.4 g (= 100%) glycogen in 20 g of meat. Next, glycogen was gradually added into H_2_O, starting from 0.2 g (= 50%) up to 1.2 g (= 300%). Added to the 20 g minced meat, which we assumed included 0.4 g glycogen, this added up to a maximum glycogen content of 1.6 g, which was equivalent to 400% of the initial glycogen concentration of pure minced meat. For all experiments, the MSOT imaging probe (MSOT Acuity CE, iThera Medical, München, Germany) was secured, fixed in a bracket, and coupled to the agarose phantom using transparent ultrasound gel (Aquasonic Clear, MDSS GmbH, Hannover, Germany).

### Study flow

The patients with diagnosed PD were compared to sex- and age-matched HV. All study participants underwent clinical standard assessments, ultrasound, MSOT and MRI imaging. For clinical standard assessment, all subjects completed a PD-specific questionnaire (R-PAct-Scale), motor function tests as well as a lung function testing. A co-registered Reflected-Ultrasound Computed Tomography (RUCT) provides US-like (B-mode) images and allows for guidance during MSOT imaging. MRI was performed of the right biceps in accordance with the previous marking. At the second MSOT site (UMG), the patients with PD received the MSOT imaging at the same anatomical localization following the UHE protocol.

### Clinical standard assessments

The Rasch-built Pompe-specific activity (R-PAct) scale is a patient-based questionnaire to specifically quantify the impact of PD on daily life and social participation^53^. Briefly, it is composed of 18 items of increasing difficulty (from “comb hair” to “running”) assessed on a rating scale ranging from (0) “unable to perform” to (1) “able to perform, but with difficulty” to (2) “able to perform with no difficulty”. The scale provides external validity, reliability and good discriminating ability. The Quick Motor Function Test (QMFT) comprised 16 items, requiring each subject to complete each item to their best ability and were rated on a scale from 0 (no muscle contraction) to 4 (normal movement), resulting in a maximum score of 64 points^51^. QMFT is sensitive for proximal muscle strength and differences in disease severity. As non-specific functional tests, we used MRC scale, timed-up-and-go-test and 6 min walking test. Muscle strength according to the MRC scale was evaluated bilaterally in the proximal regions of the upper and lower body (shoulder and hip flexion, extension, abduction, adduction, internal and external rotation), as well as in the medium (elbow and knee flexion, extension), and distal regions (hand, finger, feet and toe flexion, extension, abduction, adduction) of the body. A total score was obtained by adding up scores between 0 (no muscle contraction) to 5 (normal strength) of all 18 assessed muscle functions bilaterally, leading to a maximum of 180 points. For TUAG (Timed Up-and-Go Test), participants were seated on a stationary chair and timed from standing up to completing a single step^73^. The 6-min-walk test (6MWT) measures the distances covered in 6 min; the use of walking aids as needed was permitted^74^.

### Lung function

Conventional spirometry was completed in seated position by all subjects to quantify lung function. Parameters of interest, such as VC (vital capacity) and FEV1 (forced expiratory volume in 1 s), were assessed considering age, sex and weight. Each patient underwent a minimum of three measurements, and an average value was calculated.

### B-mode ultrasound

All B-mode ultrasound images were acquired and analyzed by a single DEGUM-certified sonographer (J.J., German Society for Ultrasound in Medicine (DEGUM), level III sonographer) using the Mindray, Zonare ZS 3 (Zonare Medical System Inc, Mountain View). All muscles were evaluated for muscle texture (coarse-, medium-, fine-granuled), echogenicity (hyper-, hypo-, echogenic), distribution pattern (in-, homogeneous, focal) and Heckmatt scale (echogenicity of muscle and bone; grade 1–4: 1 = normal muscle echo, 2 = increased muscle echo while bone echo is still distinct, 3 = increased muscle echo and reduced bone echo, 4 = very strong muscle echo and complete loss of bone echo)^30,75,76^.

### Magnetic resonance imaging acquisition and analysis

To derive the fat fraction, all participants underwent an MRI (MAGNETOM Free.Max, 0.55 T, Siemens Healthineers, Erlangen, Germany) of their right upper arm. A dedicated 6-channel surface coil was used (Siemens Healthineers, Germany). A transversal T1 turbo spin echo (TSE) Dixon sequence and a transversal T2 TSE Short-TI Inversion Recovery (STIR) were acquired. For MRI imaging analysis, manually drawn ROIs were placed in the biceps brachii muscle corresponding to the position of MSOT evaluation. Signal intensities of in-phase and fat-only images were used to estimate the fat fraction (FF). T2 TSE STIR images were read by a board-certificated radiologist with 10 years of experience in musculoskeletal imaging for the presence of intramuscular edema.

### Multispectral optoacoustic tomography

Imaging was performed by two MSOT-experienced examiners (A.L.W., R.R.) at UHE and two MSOT-experienced examiners (S.M., J.Z.) at UMG using at each site a different hybrid MSOT/RUCT (Reflected ultrasound computed tomography) imaging system (MSOT Acuity Echo, iThera Medical GmbH, Munich, Germany). As described before^28,30,41,42,66,77,78^, a handheld 2D probe (center frequency: 4 MHz, field of view: 40 × 40 mm, 256 transducer elements, spatial resolution: 150 µm) was positioned at a 90° angle, and transparent ultrasound gel was used for coupling. Laser was set to a single wavelength (SWL) starting from 700 to 1210 nm (700 nm, 730 nm, 760 nm, 800 nm, 850 nm, 910 nm, 930 nm, 950 nm, 980 nm, 1030 nm, 1080 nm, 1100 nm, 1210 nm, Hb and HbO_2_ spectrum) with a repetition rate of 25 Hz. Negative pixels were set to 0. Images required minimal motion of examiners, which was assisted by a motion bar provided by the software. A minimum of two images were taken per muscle. All participants wore safety goggles during the examination, adverse events were accordingly documented.

### MSOT data analysis

MSOT Imaging data was transferred to a workstation and iLabs software (iThera Medical GmbH, Munich, Germany) was used for image analyses. Briefly, a polygonal region of interest (ROI) was defined and drawn centrally within the biceps muscle according to its B-mode US image. The ROI was placed directly under the muscle fascia, excluding any visible vessels. The size of the ROIs in the HV data was determined by the 800 nm optoacoustic signal and included only the high-intensity area to prevent false negative and underrated data. The PD patients’ ROI size was determined by matching the area of the ROI to the one used with the matched HV (Max area Difference = 0.72 mm², Min area Difference = 0.02 mm², mean [SD] = 0.26 mm² [0.18]). Signal intensities of the above-named SWL were recorded as well as a spectral analysis to detect the MSOT parameters Hb, HbO_2_, MSOT_collagen_, and MSOT_lipid_ was performed.

### Quantitative ultrasound greyscale scoring

The iLabs software (version 1.3.16, iThera Medical GmbH, Munich, Germany) was used to extract the MSOT/RUCT images as.png files. Specifically, only the RUCT images were chosen for the greyscale (GSL) analysis. As described before^43^, the analysis was conducted using Fiji software, a distribution of the open-source ImageJ software (V2.1.0/1.53c). To facilitate this, polygonal ROIs were positioned beneath the muscle fascia. Afterward, the content within the ROIs was utilized to examine the GSL, wherein standardized mean, minimum, and maximum GSL values were quantified in arbitrary units. For further statistical analysis, only the mean values of GSL were used to enhance intermodal comparability.

### Statistical analysis

Continuous variables are given as means with SDs and categorical variables as numbers with percentages. A nonparametric Mann–Whitney test was used for unpaired comparisons. A nonparametric Wilcoxon test was used to assess differences in HV and paired PD patients. Adjusted *P*-values are reported. Prism 10, version 10.1.0 (GraphPad Software) was used for all statistical analyses. *P* < 0.05 was considered to indicate statistically significant difference in all analyses.

### Reporting summary

Further information on research design is available in the Nature Portfolio Reporting Summary linked to this article.

## Supplementary information

Supplementary Information

Peer Review File

Reporting Summary

Study protocol (Center 1, updated)

## Source data

Source Data

## Data Availability

The raw (individual, identifiable patient) data are protected and are not available due to data privacy laws. Data sharing requests will be considered on a case-by-case basis. The processed pseudonymized imaging data can be accessed upon request and within the framework of legal regulations from the corresponding author (equivalent purposes to those for which the patients grant their consent to use the data). Access is granted directly after publication for 36 months. The contact is ki-forschung@uk-erlangen.de, and response to request will be provided within 4–6 weeks. The data will be available for 3 months. The remaining data of this study are provided in the Supplementary Information and Source Data file. The study protocols and the statistical analysis plan are provided with this manuscript in the Supplementary information file. Source data are provided with this paper.

## References

[1] Kishnani, P. S. et al. Pompe disease diagnosis and management guideline. *Genet. Med.* **8**, 267–288 (2006). 16702877 10.1097/01.gim.0000218152.87434.f3 PMC3110959

[2] Kroos, M. et al. Update of the Pompe disease mutation database with 107 sequence variants and a format for severity rating. *Hum. Mutat.* **29**, E13–E26 (2008). 18425781 10.1002/humu.20745

[3] Kohler, L., Puertollano, R. & Raben, N. Pompe disease: from basic science to therapy. *Neurotherapeutics* **15**, 928–942 (2018). 30117059 10.1007/s13311-018-0655-y PMC6277280

[4] Pompe, J. C. Over idiopathische hypertrophie van het hart. *Ned. Tijdschr. Geneeskd.* **76**, 304–311 (1932).

[5] Hers, H. G. α-Glucosidase deficiency in generalized glycogen-storage disease (Pompe’s disease). *Biochem. J.* **86**, 11–16 (1963). 13954110 10.1042/bj0860011 PMC1201703

[6] van den Hout, H. M. et al. The natural course of infantile Pompe’s disease: 20 original cases compared with 133 cases from the literature. *Pediatrics* **112**, 332–340 (2003). 12897283 10.1542/peds.112.2.332

[7] van der Ploeg, A. T. & Reuser, A. J. Pompe’s disease. *Lancet* **372**, 1342–1353 (2008). 18929906 10.1016/S0140-6736(08)61555-X

[8] Kishnani, P. S. & Howell, R. R. Pompe disease in infants and children. *J. Pediatr.* **144**, S35–S43 (2004). 15126982 10.1016/j.jpeds.2004.01.053

[9] Cupler, E. J. et al. Consensus treatment recommendations for late-onset Pompe disease. *Muscle Nerve* **45**, 319–333 (2012). 22173792 10.1002/mus.22329 PMC3534745

[10] Preisler, N. et al. Late-onset Pompe disease is prevalent in unclassified limb-girdle muscular dystrophies. *Mol. Genet. Metab.* **110**, 287–289 (2013). 24011652 10.1016/j.ymgme.2013.08.005

[11] van der Ploeg, A. T. et al. A randomized study of alglucosidase alfa in late-onset Pompe’s disease. *N. Engl. J. Med.* **362**, 1396–1406 (2010). 20393176 10.1056/NEJMoa0909859

[12] Khan, A. A. et al. Higher dosing of alglucosidase alfa improves outcomes in children with Pompe disease: a clinical study and review of the literature. *Genet. Med.* **22**, 898–907 (2020). 31904026 10.1038/s41436-019-0738-0 PMC7469631

[13] Schoser, B. et al. Survival and long-term outcomes in late-onset Pompe disease following alglucosidase alfa treatment: a systematic review and meta-analysis. *J. Neurol.* **264**, 621–630 (2017). 27372449 10.1007/s00415-016-8219-8

[14] Schoser, B. et al. Safety and efficacy of cipaglucosidase alfa plus miglustat versus alglucosidase alfa plus placebo in late-onset Pompe disease (PROPEL): an international, randomised, double-blind, parallel-group, phase 3 trial. *Lancet Neurol.* **20**, 1027–1037 (2021). 34800400 10.1016/S1474-4422(21)00331-8

[15] Bolano-Diaz, C. & Diaz-Manera, J. Therapeutic options for the management of Pompe disease: current challenges and clinical evidence in therapeutics and clinical risk management. *Ther. Clin. Risk Manag.* **18**, 1099–1115 (2022). 36536827 10.2147/TCRM.S334232 PMC9759116

[16] Case, L. E., Beckemeyer, A. A. & Kishnani, P. S. Infantile Pompe disease on ERT: update on clinical presentation, musculoskeletal management, and exercise considerations. *Am. J. Med. Genet. C Semin. Med. Genet.* **160C**, 69–79 (2012). 22252989 10.1002/ajmg.c.31321

[17] van der Ploeg, A. T. et al. European consensus for starting and stopping enzyme replacement therapy in adult patients with Pompe disease: a 10-year experience. *Eur. J. Neurol.* **24**, 768-e31 (2017). 28477382 10.1111/ene.13285

[18] Tarnopolsky, M. et al. Pompe disease: diagnosis and management. Evidence-based guidelines from a Canadian expert panel. *Can. J. Neurol. Sci.* **43**, 472–485 (2016). 27055517 10.1017/cjn.2016.37

[19] Llerena Junior, J. C. et al. Guidelines for the diagnosis, treatment and clinical monitoring of patients with juvenile and adult Pompe disease. *Arq. Neuropsiquiatr.* **74**, 166–176 (2016). 26690841 10.1590/0004-282X20150194

[20] MENA Pompe Working Group. Diagnosis and treatment of late-onset Pompe disease in the Middle East and North Africa region: consensus recommendations from an expert group. *BMC Neurol.* **15**, 205 (2015). 26471939 10.1186/s12883-015-0412-3 PMC4608291

[21] Manwaring, V. et al. Urine analysis of glucose tetrasaccharide by HPLC; a useful marker for the investigation of patients with Pompe and other glycogen storage diseases. *J. Inherit. Metab. Dis.* **35**, 311–316 (2012). 21687968 10.1007/s10545-011-9360-2

[22] Young, S. P. et al. Long-term monitoring of patients with infantile-onset Pompe disease on enzyme replacement therapy using a urinary glucose tetrasaccharide biomarker. *Genet. Med.* **11**, 536–541 (2009). 19521244 10.1097/GIM.0b013e3181a87867

[23] An, Y. et al. Glucose tetrasaccharide as a biomarker for monitoring the therapeutic response to enzyme replacement therapy for Pompe disease. *Mol. Genet. Metab.* **85**, 247–254 (2005). 15886040 10.1016/j.ymgme.2005.03.010

[24] Figueroa-Bonaparte, S. et al. Quantitative muscle MRI to follow up late onset Pompe patients: a prospective study. *Sci. Rep.* **8**, 10898 (2018). 30022036 10.1038/s41598-018-29170-7 PMC6052002

[25] Figueroa-Bonaparte, S. et al. Muscle MRI findings in childhood/adult onset Pompe disease correlate with muscle function. *PLoS ONE* **11**, e0163493 (2016). 27711114 10.1371/journal.pone.0163493 PMC5053479

[26] Rehmann, R. et al. Diffusion tensor imaging reveals changes in non-fat infiltrated muscles in late onset Pompe disease. *Muscle Nerve* **62**, 541–549 (2020). 32654203 10.1002/mus.27021

[27] Gruhn, K. M. et al. Muscle imaging data in late-onset Pompe disease reveal a correlation between the pre-existing degree of lipomatous muscle alterations and the efficacy of long-term enzyme replacement therapy. *Mol. Genet. Metab. Rep.* **3**, 58–64 (2015). 26937398 10.1016/j.ymgmr.2015.03.010 PMC4750633

[28] Regensburger, A. P. et al. Detection of collagens by multispectral optoacoustic tomography as an imaging biomarker for Duchenne muscular dystrophy. *Nat. Med.* **25**, 1905–1915 (2019). 31792454 10.1038/s41591-019-0669-y

[29] Regensburger, A. P., Wagner, A. L., Claussen, J., Waldner, M. J. & Knieling, F. Shedding light on pediatric diseases: multispectral optoacoustic tomography at the doorway to clinical applications. *Mol. Cell Pediatr.* **7**, 3 (2020). 32130546 10.1186/s40348-020-00095-4 PMC7056767

[30] Regensburger, A. P. et al. Multispectral optoacoustic tomography for non-invasive disease phenotyping in pediatric spinal muscular atrophy patients. *Photoacoustics* **25**, 100315 (2022). 34849338 10.1016/j.pacs.2021.100315 PMC8607197

[31] Träger, A. P. et al. Hybrid ultrasound and single wavelength optoacoustic imaging reveals muscle degeneration in peripheral artery disease. *Photoacoustics* **35**, 100579 (2023). 10.1016/j.pacs.2023.100579 PMC10835356 38312805

[32] Ntziachristos, V. & Razansky, D. Molecular imaging by means of multispectral optoacoustic tomography (MSOT). *Chem. Rev.* **110**, 2783–2794 (2010). 20387910 10.1021/cr9002566

[33] Buehler, A., Kacprowicz, M., Taruttis, A. & Ntziachristos, V. Real-time handheld multispectral optoacoustic imaging. *Opt. Lett.* **38**, 1404–1406 (2013). 23632499 10.1364/OL.38.001404

[34] Manohar, S. & Razansky, D. Photoacoustics: a historical review. *Adv. Opt. Photon.* **8**, 586–617 (2016). 10.1364/AOP.8.000586

[35] Schellenberg, M. W. & Hunt, H. K. Hand-held optoacoustic imaging: a review. *Photoacoustics* **11**, 14–27 (2018). 30073147 10.1016/j.pacs.2018.07.001 PMC6068331

[36] Steinberg, I. et al. Photoacoustic clinical imaging. *Photoacoustics* **14**, 77–98 (2019). 31293884 10.1016/j.pacs.2019.05.001 PMC6595011

[37] Regensburger, A. P., Brown, E., Kronke, G., Waldner, M. J. & Knieling, F. Optoacoustic imaging in inflammation. *Biomedicines* **9**, 483 (2021). 10.3390/biomedicines9050483 PMC8145174 33924983

[38] Wilson, K., Homan, K. & Emelianov, S. Biomedical photoacoustics beyond thermal expansion using triggered nanodroplet vaporization for contrast-enhanced imaging. *Nat. Commun.* **3**, 618 (2012). 22233628 10.1038/ncomms1627

[39] Weber, J., Beard, P. C. & Bohndiek, S. E. Contrast agents for molecular photoacoustic imaging. *Nat. Methods* **13**, 639–650 (2016). 27467727 10.1038/nmeth.3929

[40] Razansky, D., Buehler, A. & Ntziachristos, V. Volumetric real-time multispectral optoacoustic tomography of biomarkers. *Nat. Protoc.* **6**, 1121–1129 (2011). 21738125 10.1038/nprot.2011.351

[41] Knieling, F. et al. Multispectral optoacoustic tomography for assessment of Crohn’s disease activity. *N. Engl. J. Med.* **376**, 1292–1294 (2017). 28355498 10.1056/NEJMc1612455

[42] Wagner, A. L. et al. Precision of handheld multispectral optoacoustic tomography for muscle imaging. *Photoacoustics* **21**, 100220 (2021). 33318928 10.1016/j.pacs.2020.100220 PMC7723806

[43] Danko, V. et al. Hybrid reflected-ultrasound computed tomography versus B-mode-ultrasound for muscle scoring in spinal muscular atrophy. *J. Neuroimaging*. **33**, 393–403 (2023). 10.1111/jon.13081 36627228

[44] Raben, N. et al. Enzyme replacement therapy in the mouse model of Pompe disease. *Mol. Genet. Metab.* **80**, 159–169 (2003). 14567965 10.1016/j.ymgme.2003.08.022

[45] McVie-Wylie, A. J. et al. Biochemical and pharmacological characterization of different recombinant acid alpha-glucosidase preparations evaluated for the treatment of Pompe disease. *Mol. Genet. Metab.* **94**, 448–455 (2008). 18538603 10.1016/j.ymgme.2008.04.009 PMC2774491

[46] Cardone, M. et al. Abnormal mannose-6-phosphate receptor trafficking impairs recombinant alpha-glucosidase uptake in Pompe disease fibroblasts. *Pathogenetics* **1**, 6 (2008). 19046416 10.1186/1755-8417-1-6 PMC2635360

[47] Kishnani, P. S. et al. Efficacy and safety of avalglucosidase alfa in patients with late-onset Pompe disease after 97 weeks: a phase 3 randomized clinical trial. *JAMA Neurol.* **80**, 558–567 (2023). 37036722 10.1001/jamaneurol.2023.0552 PMC10087094

[48] Raben, N., Roberts, A. & Plotz, P. H. Role of autophagy in the pathogenesis of Pompe disease. *Acta Myol.* **26**, 45–48 (2007). 17915569 PMC2949326

[49] Chien, Y. H., Hwu, W. L. & Lee, N. C. Pompe disease: early diagnosis and early treatment make a difference. *Pediatr. Neonatol.* **54**, 219–227 (2013). 23632029 10.1016/j.pedneo.2013.03.009

[50] Yang, C. F. et al. Very early treatment for infantile-onset Pompe disease contributes to better outcomes. *J. Pediatr.* **169**, 174–180.e171 (2016). 26685070 10.1016/j.jpeds.2015.10.078

[51] van Capelle, C. I. et al. The quick motor function test: a new tool to rate clinical severity and motor function in Pompe patients. *J. Inherit. Metab. Dis.* **35**, 317–323 (2012). 21912959 10.1007/s10545-011-9388-3 PMC3278629

[52] Harfouche, M. et al. Use of the patient-reported outcomes measurement information system (PROMIS(R)) to assess late-onset Pompe disease severity. *J. Patient Rep. Outcomes* **4**, 83 (2020). 33034771 10.1186/s41687-020-00245-2 PMC7547055

[53] van der Beek, N. A., Hagemans, M. L., van der Ploeg, A. T., van Doorn, P. A. & Merkies, I. S. The Rasch-built Pompe-specific activity (R-PAct) scale. *Neuromuscul. Disord.* **23**, 256–264 (2013). 23273871 10.1016/j.nmd.2012.10.024

[54] Diaz-Manera, J. et al. Safety and efficacy of avalglucosidase alfa versus alglucosidase alfa in patients with late-onset Pompe disease (COMET): a phase 3, randomised, multicentre trial. *Lancet Neurol.* **20**, 1012–1026 (2021). 34800399 10.1016/S1474-4422(21)00241-6

[55] Salabarria, S. M. et al. Advancements in AAV-mediated gene therapy for Pompe disease. *J. Neuromuscul. Dis.* **7**, 15–31 (2020). 31796685 10.3233/JND-190426 PMC7029369

[56] Byrne, B. J. et al. Pompe disease gene therapy. *Hum. Mol. Genet.* **20**, R61–R68 (2011). 21518733 10.1093/hmg/ddr174 PMC3095055

[57] Rairikar, M. V. et al. Insight into the phenotype of infants with Pompe disease identified by newborn screening with the common c.-32-13T > G “late-onset” *GAA* variant. *Mol. Genet. Metab.* **122**, 99–107 (2017). 28951071 10.1016/j.ymgme.2017.09.008 PMC5722675

[58] Fernandes, S. A. et al. Quantitative whole-body magnetic resonance imaging in children with Pompe disease: clinical tools to evaluate severity of muscle disease. *JIMD Rep.* **57**, 94–101 (2021). 33473345 10.1002/jmd2.12174 PMC7802624

[59] Carlier, P. G. et al. Skeletal muscle quantitative nuclear magnetic resonance imaging follow-up of adult Pompe patients. *J. Inherit. Metab. Dis.* **38**, 565–572 (2015). 25749708 10.1007/s10545-015-9825-9 PMC4432102

[60] Lollert, A. et al. Quantification of intramuscular fat in patients with late-onset Pompe disease by conventional magnetic resonance imaging for the long-term follow-up of enzyme replacement therapy. *PLoS ONE* **13**, e0190784 (2018). 29315315 10.1371/journal.pone.0190784 PMC5760036

[61] Baligand, C. et al. ^13^C/^31^P MRS metabolic biomarkers of disease progression and response to AAV delivery of hGAA in a mouse model of Pompe disease. *Mol. Ther. Methods Clin. Dev.* **7**, 42–49 (2017). 29018835 10.1016/j.omtm.2017.09.002 PMC5626920

[62] Beha, G. et al. FP.19 Quantification of glycogen distribution in late-onset Pompe patients using 7 Tesla C13 NMR spectroscopy. *Neuromuscul. Disord.* **32**, S73 (2022). 10.1016/j.nmd.2022.07.132

[63] Bohndiek, S. Addressing photoacoustics standards. *Nat. Photonics* **13**, 298–298 (2019). 10.1038/s41566-019-0417-3

[64] Assi, H. et al. A review of a strategic roadmapping exercise to advance clinical translation of photoacoustic imaging: from current barriers to future adoption. *Photoacoustics* **32**, 100539 (2023). 37600964 10.1016/j.pacs.2023.100539 PMC10432856

[65] Paulus, L. P. et al. Contrast-enhanced multispectral optoacoustic tomography for functional assessment of the gastrointestinal tract. *Adv. Sci*. **10**, e2302562 (2023). 10.1002/advs.202302562 PMC10427354 37289088

[66] Paulus, L. P. et al. Multispectral optoacoustic tomography of the human intestine—temporal precision and the influence of postprandial gastrointestinal blood flow. *Photoacoustics* **30**, 100457 (2023). 36824387 10.1016/j.pacs.2023.100457 PMC9942118

[67] Waldner, M. J. et al. Multispectral optoacoustic tomography in Crohn’s disease: noninvasive imaging of disease activity. *Gastroenterology* **151**, 238–240 (2016). 27269244 10.1053/j.gastro.2016.05.047

[68] Regensburger, A. P. et al. Multispectral optoacoustic tomography enables assessment of disease activity in paediatric inflammatory bowel disease. *Photoacoustics* **35**, 100578 (2024). 38144890 10.1016/j.pacs.2023.100578 PMC10746560

[69] Tascilar, K. et al. Non-invasive metabolic profiling of inflammation in joints and entheses by multispectral optoacoustic tomography. *Rheumatology* **62**, 841–849 (2023). 35699479 10.1093/rheumatology/keac346

[70] Gunther, J. S. et al. Targeting muscular hemoglobin content for classification of peripheral arterial disease by noninvasive multispectral optoacoustic tomography. *JACC Cardiovasc. Imaging* **16**, 719–721 (2023). 36752442 10.1016/j.jcmg.2022.11.010

[71] Stoffels, I. et al. Metastatic status of sentinel lymph nodes in melanoma determined noninvasively with multispectral optoacoustic imaging. *Sci. Transl. Med.* **7**, 317ra199 (2015). 26659573 10.1126/scitranslmed.aad1278

[72] Grohl, J., Schellenberg, M., Dreher, K. & Maier-Hein, L. Deep learning for biomedical photoacoustic imaging: a review. *Photoacoustics* **22**, 100241 (2021). 33717977 10.1016/j.pacs.2021.100241 PMC7932894

[73] Podsiadlo, D. & Richardson, S. The timed “Up & Go”: a test of basic functional mobility for frail elderly persons. *J. Am. Geriatr. Soc.* **39**, 142–148 (1991). 1991946 10.1111/j.1532-5415.1991.tb01616.x

[74] ATS Committee on Proficiency Standards for Clinical Pulmonary Function Laboratories. ATS statement: guidelines for the six-minute walk test. *Am. J. Respir. Crit. Care Med*. **166**, 111–117 (2002). 10.1164/ajrccm.166.1.at1102 12091180

[75] Heckmatt, J., Rodillo, E., Doherty, M., Willson, K. & Leeman, S. Quantitative sonography of muscle. *J. Child Neurol.* **4**, S101–S106 (1989). 2681371 10.1177/0883073889004001S15

[76] Heckmatt, J. Z., Leeman, S. & Dubowitz, V. Ultrasound imaging in the diagnosis of muscle disease. *J. Pediatr.* **101**, 656–660 (1982). 7131136 10.1016/S0022-3476(82)80286-2

[77] Waldner, M. J. et al. Multispectral optoacoustic tomography in Crohn’s disease: non-invasive imaging of disease activity. *Gastroenterology* **151**, 238–240 (2016). 10.1053/j.gastro.2016.05.047 27269244

[78] Günther, J. S. et al. Targeting muscular hemoglobin content for classification of peripheral arterial disease by noninvasive multispectral optoacoustic tomography. *JACC Cardiovasc. Imaging* **16**, 719–721 (2023). 10.1016/j.jcmg.2022.11.010 36752442

