# Supplementary material for: Non-invasive optoacoustic imaging of glycogen-storage and muscle degeneration in late-onset Pompe disease
Source: Nat Commun. 2024 Sep 8;15:7843. doi: 10.1038/s41467-024-52143-6 (PMC11381542; doi:10.1038/s41467-024-52143-6)
Supplement: Supplementary file 1 — Supplementary Information [file 41467_2024_52143_MOESM1_ESM.pdf]

Supplementary Information

Title: Non-invasive optoacoustic imaging of glycogen-storage and muscle degeneration in Late-onset Pompe disease

Authors: Lina Tan[1,2], Jana Zschüntzsch[3], Stefanie Meyer[3], Alica Stobbe[3], Hannah Bruex[3], Adrian P. Regensburger[1,2], Merle Claßen[1,2], Frauke Alves[4,5], Jörg Jüngert[1], Ulrich Rother[6], Yi Li[6], Vera Danko[1,2], Werner Lang[6], Matthias Türk[7], Sandy Schmidt[8], Matthias Vorgerd[9,10], Lara Schlaffke[9], Joachim Woelfle[1], Andreas Hahn[11], Alexander Mensch[12], Martin Winterholler[13], Regina Trollmann[1,14], Rafael Heiß[8], Alexandra L. Wagner[1,2,15,16], Roman Raming[1,2,16], Ferdinand Knieling[1,2,16,17,*]

[1]Department of Pediatrics and Adolescent Medicine; University Hospital Erlangen; Friedrich-Alexander-Universität (FAU) Erlangen-Nürnberg; Erlangen, 91054; Germany.

[2]Translational Pediatrics, Department of Pediatrics and Adolescent Medicine, University Hospital Erlangen; Friedrich-Alexander-Universität (FAU) Erlangen-Nürnberg; Erlangen, 91054; Germany.

[3]Neuromuscular Disease Research; Clinic for Neurology; University Medical Center Göttingen (UMG); Göttingen, 37075; Germany

[4]Translational Molecular Imaging, Max-Planck Institute for Multidisciplinary Sciences (MPI-NAT), City Campus, Göttingen, 37075, Germany

[5]Clinic for Haematology and Medical Oncology, Institute of Diagnostic and Interventional Radiology, University Medical Center Göttingen (UMG), Göttingen, 37075; Germany.

[6]Department of Vascular Surgery, University Hospital Erlangen, Friedrich-Alexander-Universität (FAU) Erlangen-Nürnberg; Erlangen, 91054; Germany.

[7]Department of Neurology, University Hospital Erlangen, Friedrich-Alexander-Universität (FAU) Erlangen-Nürnberg; Erlangen, 91054; Germany.

[8]Institute of Radiology, University Hospital Erlangen; Friedrich-Alexander-Universität (FAU) Erlangen-Nürnberg; Erlangen, 91054; Germany.

[9]Department of Neurology, BG-University Hospital Bergmannsheil; Ruhr-University Bochum; 44789 Bochum, Germany

[10]Heimer Institute for Muscle Research; BG-University Hospital Bergmannsheil; 44789 Bochum, Germany.

[11]Department of Child Neurology; Justus-Liebig-Universität Giessen; 35385 Giessen, Germany.

[12]Department of Neurology; Martin-Luther-Universität Halle-Wittenberg; 06120 Halle (Saale), Germany.

[13]Sana Krankenhaus Rummelsberg; 90489 Nuremberg/Schwarzenbruck, Germany

[14]Center for Social Pediatrics, University Hospital Erlangen: Friedrich-Alexander-Universität (FAU) Erlangen-Nürnberg; Erlangen, 91054; Germany.

[15]Department of Pediatric Neurology; Center for Chronically Sick Children; Charité Berlin; 13353 Berlin, Germany.

[16]These authors contributed equally

[17]Lead contact

*Correspondence: ferdinand.knieling@uk-erlangen.de (F.K.)

Content

CONTENT 2

1. SUPPLEMENTARY FIGURES 3

2. STUDY PROTOCOL (INITIAL VERSION) 12

3. STUDY PROTOCOL (AMENDED VERSION) 40

4. STUDY PROTOCOL (EXTERNAL COHORT) 70

5. STATISTICAL ANALYSIS PLAN (SAP) 98

1. Supplementary Figures

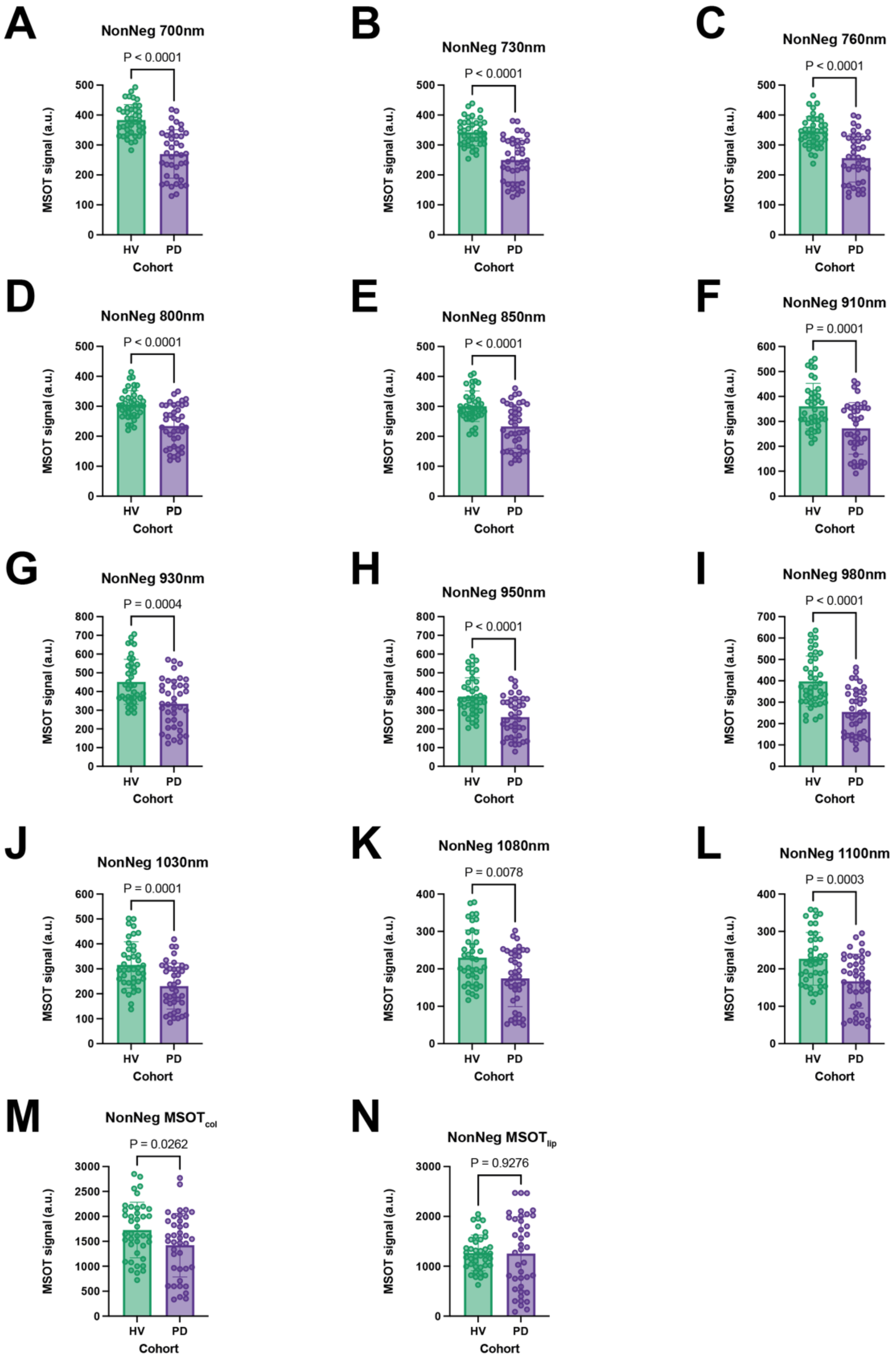
A
NonNeg 700nm
P < 0.0001
B
NonNeg 730nm
P < 0.0001
C
NonNeg 760nm
P < 0.0001
D
NonNeg 800nm
P < 0.0001
E
NonNeg 850nm
P < 0.0001
F
NonNeg 910nm
P = 0.0001
G
NonNeg 930nm
P = 0.0004
H
NonNeg 950nm
P < 0.0001
I
NonNeg 980nm
P < 0.0001
J
NonNeg 1030nm
P = 0.0001
K
NonNeg 1080nm
P = 0.0078
L
NonNeg 1100nm
P = 0.0003
M
NonNeg $MSOT_{col}$
P = 0.0262
N
NonNeg $MSOT_{lip}$
P = 0.9276
MSOT signal (a.u.)
HV
PD
Cohort

Figure S1. MSOT Quantification of biceps muscle using non-negative signals. Comparison of signal intensity for single wavelength 700 nm (**A**), 730 nm (**B**), 760nm (**C**), 800nm (**D**), 850nm (**E**), 910nm (**F**), 930nm (**G**), 950nm (**H**), 980nm (**I**), 1030nm (**J**), 1080nm (**K**), 1100nm (**L**), $MSOT_{col}$ (**M**) and $MOST_{lip}$ (**N**) between HV and PD. MSOT = Multispectral Optoacoustic Tomography, HV = healthy volunteers, PD = Pompe disease patients, Spec C = Spectrum Collagen = MSOT- derived collagen signal ($MSOT_{col}$), Spec L = Spectrum Lipid = MSOT- derived lipid signal ($MSOT_{lip}$). Results represent 80 data sets from n = 40 independent biceps muscle regions (n = 20 HV/n = 20 LOPD) in n = 20 biologically independent subjects (n = 10 HV and n = 10 patients with LOPD). Each bar displays the mean of top 10% MSOT signal of the biceps muscle of a whole proband group with the error bars indicating SD (green bar/dots = HV and violet bar/dots = LOPD). Statistical difference was tested with Welch's t test.

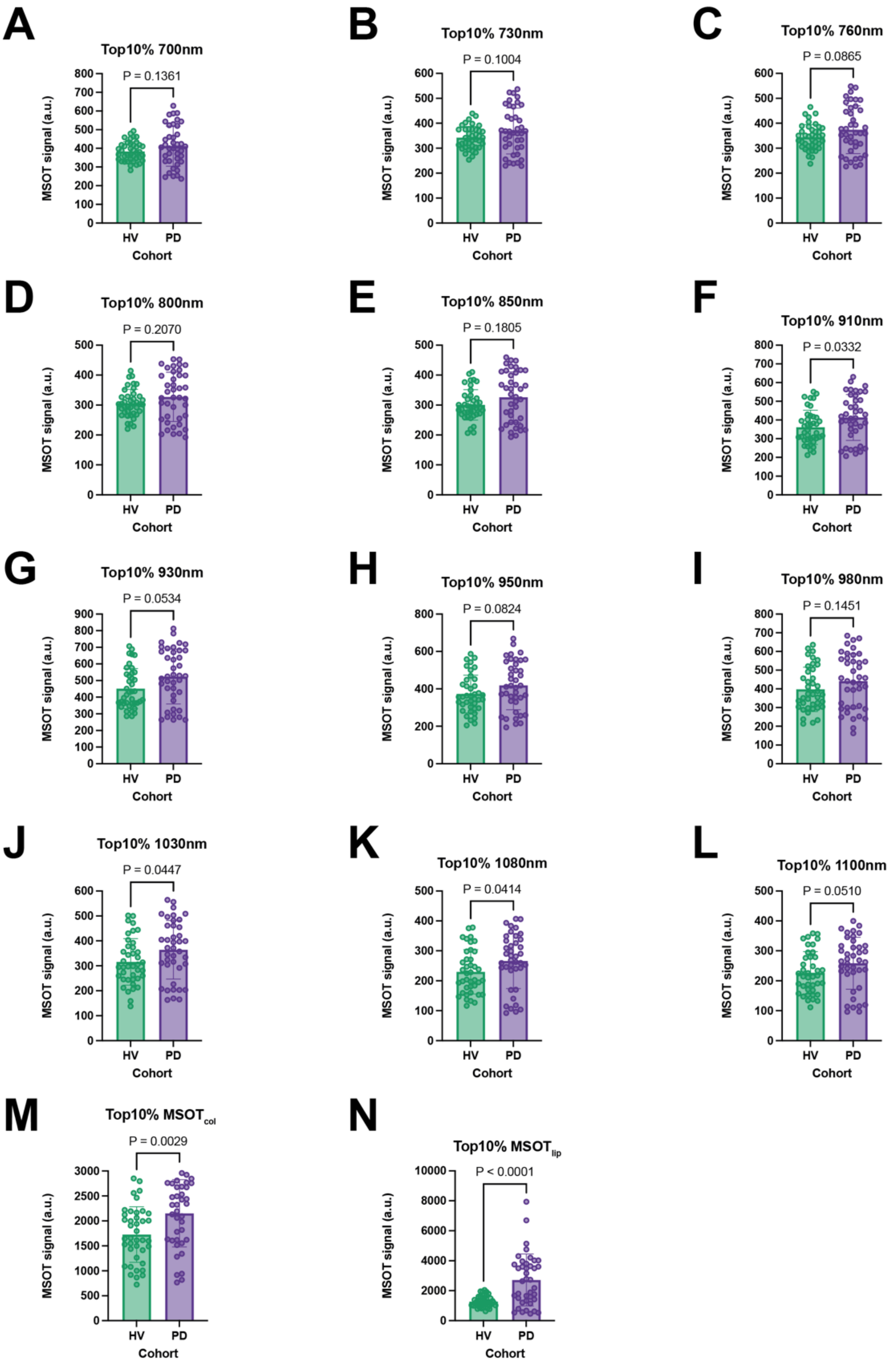
A
Top10% 700nm
P = 0.1361
B
Top10% 730nm
P = 0.1004
C
Top10% 760nm
P = 0.0865
D
Top10% 800nm
P = 0.2070
E
Top10% 850nm
P = 0.1805
F
Top10% 910nm
P = 0.0332
G
Top10% 930nm
P = 0.0534
H
Top10% 950nm
P = 0.0824
I
Top10% 980nm
P = 0.1451
J
Top10% 1030nm
P = 0.0447
K
Top10% 1080nm
P = 0.0414
L
Top10% 1100nm
P = 0.0510
M
Top10% MSOT$_{col}$
P = 0.0029
N
Top10% MSOT$_{lip}$
P < 0.0001
MSOT signal (a.u.)
HV
PD
Cohort

Figure S2. **MSOT Quantification of biceps muscle using top 10% of total signals.** Comparison of signal intensity for single wavelength 700 nm (**A**), 730 nm (**B**), 760nm (**C**), 800nm (**D**), 850nm (**E**), 910nm (**F**), 930nm (**G**), 950nm (**H**), 980nm (**I**), 1030nm (**J**), 1080nm (**K**), 1100nm (**L**), $MSOT_{col}$ (**M**) and $MOST_{lip}$ (**N**) between HV and PD. MSOT = Multispectral Optoacoustic Tomography, HV = healthy volunteers, PD = Pompe disease patients, Spec C = Spectrum Collagen = MSOT- derived collagen signal ($MSOT_{col}$), Spec L = Spectrum Lipid = MSOT- derived lipid signal ($MSOT_{lip}$). Results represent 80 data sets from n = 40 independent biceps muscle regions (n = 20 HV/n = 20 LOPD) in n = 20 biologically independent subjects (n = 10 HV and n = 10 patients with LOPD). Each bar displays the mean of top 10% MSOT signal of the biceps muscle of a whole proband group with the error bars indicating SD (green bar/dots = HV and violet bar/dots = LOPD). Statistical difference was tested with Welch's t test.

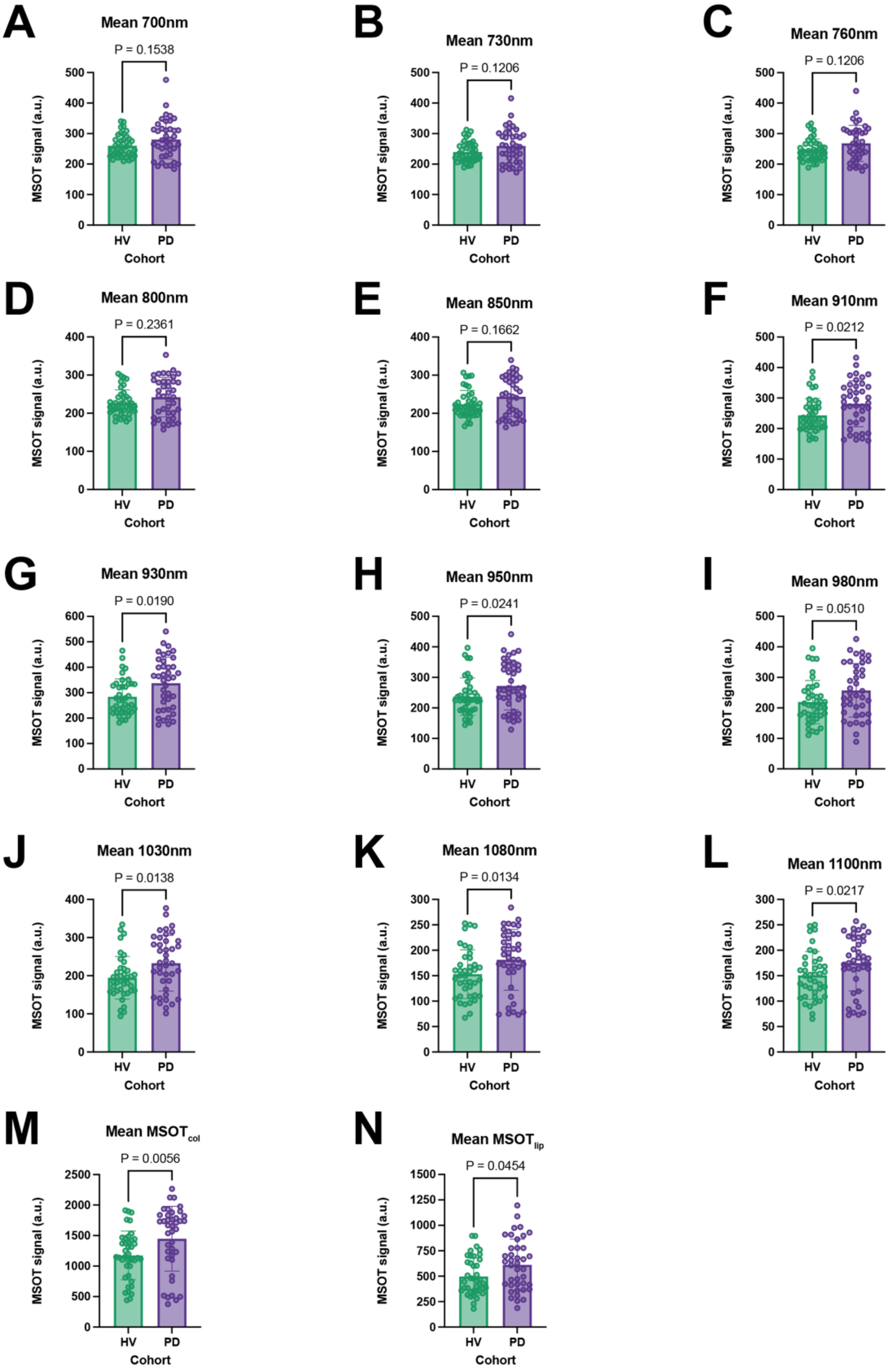
A
Mean 700nm
P = 0.1538
B
Mean 730nm
P = 0.1206
C
Mean 760nm
P = 0.1206
D
Mean 800nm
P = 0.2361
E
Mean 850nm
P = 0.1662
F
Mean 910nm
P = 0.0212
G
Mean 930nm
P = 0.0190
H
Mean 950nm
P = 0.0241
I
Mean 980nm
P = 0.0510
J
Mean 1030nm
P = 0.0138
K
Mean 1080nm
P = 0.0134
L
Mean 1100nm
P = 0.0217
M
Mean MSOT$_{col}$
P = 0.0056
N
Mean MSOT$_{lip}$
P = 0.0454
MSOT signal (a.u.)
HV
PD
Cohort

Figure S3. **MSOT Quantification of biceps muscle using mean signals.** Comparison of signal intensity for single wavelength 700 nm (**A**), 730 nm (**B**), 760nm (**C**), 800nm (**D**), 850nm (**E**), 910nm (**F**), 930nm (**G**), 950nm (**H**), 980nm (**I**), 1030nm (**J**), 1080nm (**K**), 1100nm (**L**), $MSOT_{col}$ (**M**) and $MOST_{lip}$ (**N**) between HV and PD. MSOT = Multispectral Optoacoustic Tomography, HV = healthy volunteers, PD = Pompe disease patients, Spec C = Spectrum Collagen = MSOT- derived collagen signal ($MSOT_{col}$), Spec L = Spectrum Lipid = MSOT-derived lipid signal ($MSOT_{lip}$). Results represent 80 data sets from n = 40 independent biceps muscle regions (n = 20 HV/n = 20 LOPD) in n = 20 biologically independent subjects (n = 10 HV and n = 10 patients with LOPD). Each bar displays the mean of top 10% MSOT signal of the biceps muscle of a whole proband group with the error bars indicating SD (green bar/dots = HV and violet bar/dots = LOPD). Statistical difference was tested with Welch's t test.

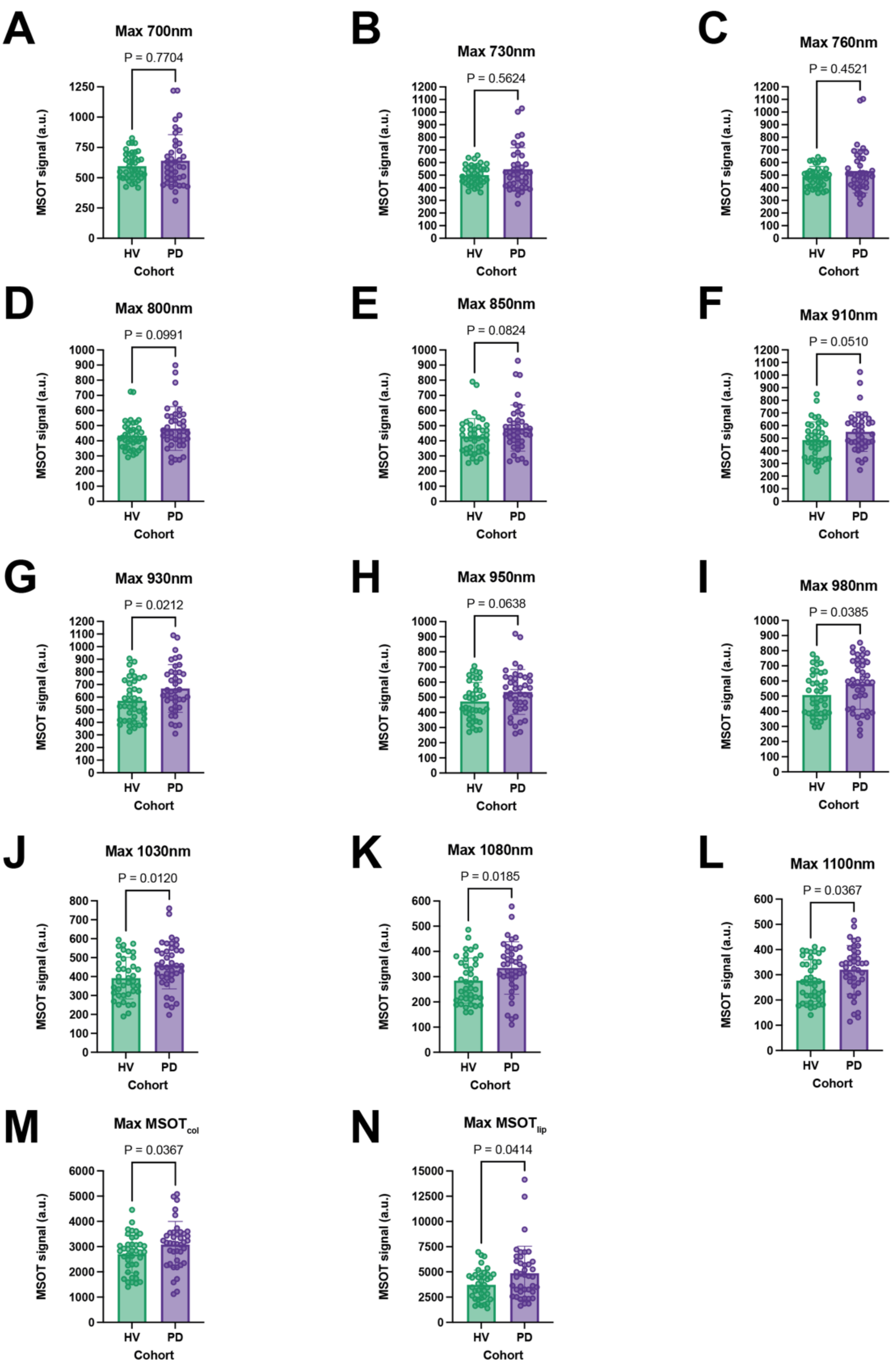
A
Max 700nm
P = 0.7704
B
Max 730nm
P = 0.5624
C
Max 760nm
P = 0.4521
D
Max 800nm
P = 0.0991
E
Max 850nm
P = 0.0824
F
Max 910nm
P = 0.0510
G
Max 930nm
P = 0.0212
H
Max 950nm
P = 0.0638
I
Max 980nm
P = 0.0385
J
Max 1030nm
P = 0.0120
K
Max 1080nm
P = 0.0185
L
Max 1100nm
P = 0.0367
M
Max $MSOT_{col}$
P = 0.0367
N
Max $MSOT_{lip}$
P = 0.0414
MSOT signal (a.u.)
HV
PD
Cohort

Figure S4. **MSOT Quantification of biceps muscle using maximum signals.** Comparison of signal intensity for single wavelength 700 nm (**A**), 730 nm (**B**), 760nm (**C**), 800nm (**D**), 850nm (**E**), 910nm (**F**), 930nm (**G**), 950nm (**H**), 980nm (**I**), 1030nm (**J**), 1080nm (**K**), 1100nm (**L**), $MSOT_{col}$ (**M**) and $MOST_{lip}$ (**N**) between HV and PD. MSOT = Multispectral Optoacoustic Tomography, HV = healthy volunteers, PD = Pompe disease patients, Spec C = Spectrum Collagen = MSOT- derived collagen signal ($MSOT_{col}$), Spec L = Spectrum Lipid = MSOT-derived lipid signal ($MSOT_{lip}$). Results represent 80 data sets from n = 40 independent biceps muscle regions (n = 20 HV/n = 20 LOPD) in n = 20 biologically independent subjects (n = 10 HV and n = 10 patients with LOPD). Each bar displays the mean of top 10% MSOT signal of the biceps muscle of a whole proband group with the error bars indicating SD (green bar/dots = HV and violet bar/dots = LOPD). Statistical difference was tested with Welch's t test.

2. Study Protocol (Initial Version)

Study protocol

SPOT_PD

Multi**sp**ectral **O**ptoacoustic **T**omography for Translational Molecular Imaging in **P**ompe **D**isease

Content is confidential.

1. Table of contents

1. Table of contents 2
2. Study titel, version number, version date 3
3. Project summary 4
4. Project summary (German) 5
5. Responsabilities 6
6. Scientific background 7
7. Study objective 12
8. Endpoint parameters 14
9. Study design 18
10. Study population 20
11. Study flow 21
12. Benefit-risk-assessment 24
13. Biometrics 25
14. Data management and protection 26
15. Biomaterial handling 26
16. Individual paticipant insurance 27
17. Signatures 27

Content is confidential.

2. Study titel, version number, version date

Study title

Multispectral Optoacoustic Tomography for Translational Molecular Imaging in Pompe Disease

Version number

Version 1.0

Version date

11.06.2021

Protocol versions

Date	Version	Status	Changes
03.01.2020	1.0	Outline	
11.06.2021	1.1	Final	

Content is confidential.

3. Project summary

In patients with Pompe disease (PD) a progressive abnormal lysosomal glycogen storage in muscle tissue leads to impaired muscle function and to degeneration of muscle fibers. Children and adults with PD present with limb-girdle muscular weakness, diaphragm weakness and impaired breathing ability. Further, patients with classic infantile PD suffer from hypertrophic cardiomyopathy. To date, the muscle pathology and the extent of the disease can be assessed using invasive techniques (e.g., muscle biopsies) or imaging (e.g., MRI). These techniques are time consuming, and especially in young patients, require anesthesia, which increases the acute risk of respiratory failure.

Multispectral optoacoustic tomography (MSOT) allows the detection of specific endogenous chromophores like collagen, myoglobin or hemoglobin by using a non-invasive approach comparable to conventional ultrasound. Instead of sound waves, MSOT illuminates tissue with near-infrared light of transient energy, which is absorbed and results in thermo-elastic expansion of certain molecules. This expansion generates ultrasound waves that are detected by the same device. Multispectral illumination and unmixing then allows the precise localisation and quantification of muscle-specific subcellular structures. MSOT has already been demonstrated the potential to visualize the muscular structure and the clinical extent of muscular disease in patients with Duchenne muscle dystrophy and differentiates those patients from healthy volunteers.

The aim of the study is to establish glycogen as a novel PD-specific imaging target using MSOT-imaging. Furthermore, we will apply MSOT-imaging to identify a PD-specific muscle pathology-signature by quantification of already established targets (collagen, myoglobin, hemoglobin, glycogen if applicable). This signature will aid in differentiating PD from other muscular pathologies and healthy volunteers and will ultimately serve as a potential non-invasive monitoring biomarker.

Content is confidential.

4. Project summary (German)

Bei Patienten mit Morbus Pompe (PD) führt eine fortschreitende abnorme lysosomale Glykogenspeicherung im Muskelgewebe zu einer Beeinträchtigung der Muskelfunktion und zur Degeneration von Muskelfasern. Kinder und Erwachsene mit Morbus Pompe zeigen eine Schwäche der Gliedergürtelmuskulatur, eine Zwerchfellschwäche und eine Beeinträchtigung der Atemfunktion. Außerdem leiden Patienten mit klassischer infantiler PD an einer hypertrophen Kardiomyopathie. Bislang können die Muskelpathologie und das Ausmaß der Erkrankung mit invasiven Techniken (z. B. Muskelbiopsien) oder bildgebenden Verfahren (z. B. MRT) beurteilt werden. Diese Techniken sind zeitaufwendig und erfordern insbesondere bei jungen Patienten eine Anästhesie, was das akute Risiko eines Atemversagens erhöht.

Die multispektrale optoakustische Tomographie (MSOT) ermöglicht den Nachweis spezifischer körpereigener Chromophore wie Kollagen, Myoglobin oder Hämoglobin durch einen nicht-invasiven Ansatz, der mit konventionellem Ultraschall vergleichbar ist. Anstelle von Schallwellen beleuchtet MSOT das Gewebe mit Nahinfrarotlicht von transienter Energie, das absorbiert wird und zu einer thermoelastischen Ausdehnung bestimmter Moleküle führt. Diese Ausdehnung erzeugt Ultraschallwellen, die von demselben Gerät detektiert werden. Die multispektrale Beleuchtung und Entmischung ermöglichen dann die präzise Lokalisierung und Quantifizierung von muskelspezifischen subzellulären Strukturen. MSOT hat bereits das Potential gezeigt, die Muskelstruktur und das klinische Ausmaß der Muskelerkrankung bei Patienten mit Duchenne-Muskeldystrophie sichtbar zu machen und diese Patienten von gesunden Freiwilligen zu unterscheiden.

Ziel der Studie ist es, Glykogen als neuartiges PD-spezifisches Bildgebungsziel mittels MSOT-Bildgebung zu etablieren. Darüber hinaus werden wir MSOT anwenden, um eine PD-spezifische Muskelpathologie-Signatur durch Quantifizierung bereits etablierter Targets (Kollagen, Myoglobin, Hämoglobin, Glykogen, falls zutreffend) zu identifizieren. Diese Signatur wird helfen, PD von anderen muskulären Pathologien und gesunden Probanden zu unterscheiden und wird letztendlich möglicherweise als potenzieller nicht-invasiver Überwachungs-Biomarker dienen.

Content is confidential.

5. Responsabilities

Study director

Dr. Ferdinand Knieling

Pediatric Experimental and Translational Imaging Laboratory (PETI_Lab)

Department of Pediatrics
University Hospital Erlangen
Loschgestr. 15
91054 Erlangen
Germany
Tel. 09131 8533118
Mail: ferdinand.knieling@uk-erlangen.de

Prof. Dr. Regina Trollmann

Head of the Department of Neuropediatrics

Department of Pediatrics
University Hospital Erlangen
Loschgestr. 15
91054 Erlangen
Germany
Tel.: 09131 85 33753
Mail: regina.trollmann@uk-erlangen.de

Sponsoring

Investigator-initated trial, funding by Sanofi Genzyme

6. Scientific background

Pompe disease (PD) is a rare, autosomal-recessive disorder caused by deficiency of the lysosomal acid alpha-glucosidase enzyme (GAA), leading to generalized build-up of glycogen, especially in the heart, muscle, liver and nervous system (Pompe, 1932, Hers, 1963). Among the glycogen storage diseases, PD is the only one with a defect in lysosomal metabolism.

PD is considered as a progressive disease with variation by age of onset, severity of organ involvement and degree of myopathy. This great phenotypic variability has led to the creation of types based on the age of onset and degree of organ involvement. They all have in common, that symptoms of affected patients are expected to worsen over time if left untreated. The classification is generally based on the age of onset as infantile (infantile onset Pompe disease, IOPD) when it presents during the first 12 months of life and late-onset (LOPD) when first symptoms appear after 12 months of age. If cardiomyopathy is present, IODP is generally referred to as classic Pompe disease (however there may be variably classification in the literature with the infantile or childhood forms). Clinically, infants with classic PD present during the first few months of life with rapidly progressive disease characterized by prominent hypertrophic cardiomyopathy, hepatomegaly, hypotonia, generalized muscle weakness, macroglossia, feeding difficulties and respiratory insufficiency. Mortality rate is high by one year of age if untreated (Kishnani 2004, 2006). Patients with non-classic PD will usually present within the first year of life with motor developmental delay and weakness, but without clinically relevant cardiac involvement. The rate of clinical progression is slower in these children and without treatment, death will usually occur in childhood as a result of respiratory insufficiency (Hers 1963, Slonim 2000). LODP include childhood and adult-onset PD. These patients generally present with slowly progressive limb girdle type weakness and respiratory insufficiency without significant cardiomyopathy (Cupler 2012, Preisler 2013). The diagnosis of PD is usually established by the typical clinical presentation, followed by confirmation of GAA deficiency in dried blood spots, e.g. through new-born screening (Bodamer, 2017). Further (confirmatory) methods include GAA activity measurement in lymphocytes, muscle or skin fibroblasts, as well as GAA mutation testing (Van den Hout, 2003, Case, 2012). All of them are invasive techniques. Early identification is important as it will likely significantly improve the outcome for all patients with PD as treatment can be initiated earlier. Treating the underlying cause of PD involves the replacement of the missing enzyme GAA via enzyme replacement therapy (ERT) with alglucosidase alfa (recombinant human GAA, rhGAA). Currently, this is the only specific treatment approved for PD. In classical IOPD, treatment significantly lengthens survival and improves motor development and respiratory and cardiac function (Kishnani, 2007). The sooner ERT begins, the better are the results (Chien, 2013). With ERT being one

very important aspect of care, patients will also need a multidisciplinary approach to ensure that all aspects of the disease are addressed.

Regardless of age of onset and severity, all patients with PD should be monitored prospectively (Kishnani, 2006, Bembi, 2008). However, there is lack of standardization across centers. A variety of clinical evaluations and tests are currently used for monitoring Pompe's disease, which may include laboratory tests including CK, AST, ALT, and LDH, cardiologic tests including electrocardiogram and echocardiogram and respiratory tests including sleep studies and breathing tests to measure lung capacity. To quantify muscle involvement electromyography is an option as well as clinical tests including 6 minutes walking test or timed to up and go test. Muscle MRI of affected patients often show fatty degeneration of muscles. One study showed that muscle MRI correlates with muscle function in patients with adult-onset Pompe disease (Figueroa-Bonaparte, 2016). Another study suggested that muscle imaging data in late-onset Pompe disease reveal a correlation between the pre-existing degree of lipomatous muscle alterations and the efficacy of long-term enzyme replacement therapy (Gruhn, 2015). For small children, however, there is always a need for sedation for MRI's, limiting its use. Therefore, ultrasound is another option to examine children's muscles.

At the moment there are no prospective biomarkers available to detect muscle degeneration at an early age and/or to follow up disease progression or ERT-treated patients. Within the last years our multidisciplinary research team (Medical Department 1, Department of Pediatrics and Adolescent Medicine, University Hospital Erlangen) published a novel non-invasive imaging modality to be able to detect subcellular tissue composition in vivo. Multi-spectral optoacoustic tomography (MSOT), an imaging technology comparable to ultrasound, allows quantitative imaging in patients of all ages (including the non-sedated child).

Content is confidential.

For MSOT, similar to a conventional ultrasound, an ultrasound transducer is positioned on the patient's skin. Instead of sound waves, MSOT illuminates tissue with light of transient energy, typically near-infrared laser light pulses, which are absorbed by the tissue, resulting in thermo-elastic expansion. This expansion gives rise to ultrasound waves that are detected by the same device. Studies have already shown that MSOT-based assessment of hemoglobin levels in the intestinal wall has the potential for Assessment of Crohn's Disease Activity (Knieling 2017, Waldner 2016). With a newly configurated device (Acuity Echo, iThera Medical GmbH, Munich, custom-built platform) an extended spectrum of laser light can be used, allowing the detection of not only hemoglobin but also further biomarkers like collagen or lipid. In our recent study, we were able to show the molecular composition of muscles in Duchenne muscle dystrophy via MSOT suggesting non-invasive measured collagen content as a novel biomarker for disease severity (Regensburger, 2019). In this study we want to establish glycogen as a novel PD-specific imaging target using MSOT-imaging.

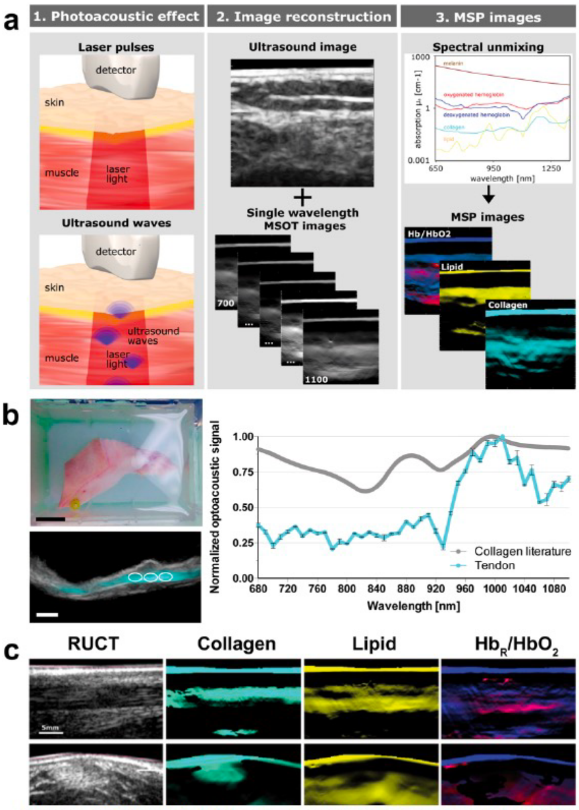

Figure 1 - MSOT principle (a), experimental preliminary work (b), and first time representation of collagen in vivo (c)

Content is confidential.

Figures 1 and 2 show exemplary MSOT images. In the light of the progression and the sometimes severe course of PD, this method would for the first time allow a non-invasive and quantitative determination of the molecular composition of muscle tissue. In this first pilot study in patients with PD, it will now be investigated whether the differences in the muscle composition of healthy volunteers and PD patients can be quantified and whether this could simultaneously be used as marker during ERT-therapy. In the future, this could generate a completely new, non-invasive method to evaluate endogenous biomarkers for therapy response.

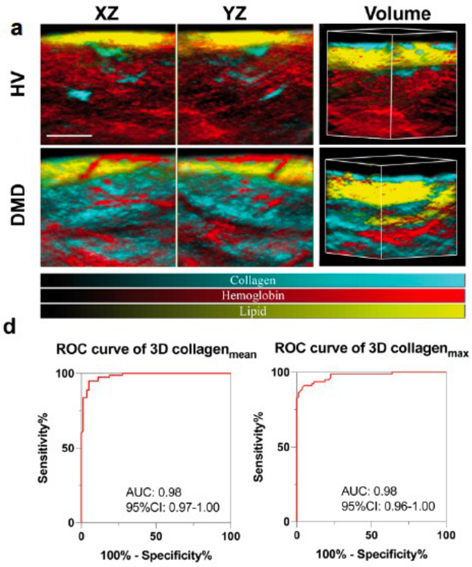

Figure 2 - Feasibility of 3D MSOT image (a) and diagnostic quality (b).

Literature

Pompe JC. Over idiopatische hypertrophie van het hart. Ned Tijdschr Geneeskd. 1932; 76; 304-12.

Hers HG. Alpha-Glucsidase deficiency in generalized glycogen storage diease (Pompe's disease), Biochem J. 1963, 86, 11-6.

Kishnani PS, Howell RR. Pompe disease in infants and children. J Pediatr. 2004;144(Suppl 5):S35-43.

Kishnani PS, Hwu WL, Mandel H, Nicolino M, Yong F, Corzo D. A retrospective, multinational, multicenter study on the natural history of infantile-onset Pompe disease. J Pediatr. 2006;148(5): 671-6.

Hers HG. Alpha-Glucosidase deficiency in generalized glycogen storage disease (Pompe's disease) Biochem J. 1963;86:11–16.

Slonim AE, Bulone L, Ritz S, Goldberg T, et al. Identification of two subtypes of infantile acid maltase deficiency. J Pediatr. 2000;137:283–285.

Cupler EJ, Consensus treatment recommendations for late-onset Pompe disease. Muscle Nerve. 2012, 45:319-33.

Preisler N. Late-onset Pompe disease is prevalent in unclassified limb-girdl muscular dystrophies. Mol Genet Metab. 2013, 110:287-9.

Bodamer OA. Newborn Screening for Pompe Disease. Pediatrics, 2017. Jul; 140 (Suppl 1) S4-S13

Van den Hout HM. The natural course of infantile Pompe's disease: 20 original cases compared with 133 cases from the literature. Pediatrics. 2003, 112.332-40.
Case LE. Infantile Pompe disease on ERT. Update on clinical presentation, musculoskeletal management and exercise considerations. Am J Med Genet C Semin Med Genet. 2012. 160C: 69-79.

Content is confidential.

Lumyzyme (alglucosidase alfa) for injection, for intravenous use (prescribing information). Cambridge, MA: Genzyme Corporation; 2014.

Myozyme (alglucosidase alfa) for injection, for intravenous use (prescribing information). Cambridge, MA: Genzyme Corporation; 2014.

Kishani PS, Recombinant human Recombinant human acid [alpha]-glucosidase: major clinical benefits in infantile-onset Pompe disease. Neurology, 2007, Jan 9; 68 82); 99-109.

Chien YH, Hwu WL, Lee NC. Pompe disease: early diagnosis and early treatment make a difference. Pediatr Neonatol. 2013;54(4):219–227

Kishnani PS, Steiner RD, Bali D,et al. Pompe disease diagnosis and management guideline [published correction appears in Genet Med. 2006;8(6):382]. Genet Med. 2006;8(5):267–288

Bembi B, Cerini E, Danesino C,et al. Management and treatment of glycogenosis type II. Neurology. 2008;71(23 suppl 2):S12–S36

Figueroa-Bonaparte S, Muscle MRI Findings in Childhood/Adult Onset Pompe Disease Correlate with Muscle Function, PLoS One. 2016; 11(10): e0163493.

Gruhn KM, Muscle imaging data in late-onset Pompe disease reveal a correlation between the pre-existing degree of lipomatous muscle alterations and the efficacy of long-term enzyme replacement therapy. Mol Genet Metab Rep. 2015 Jun; 3: 58–64.

Knieling F. Multispectral Optoacoustic Tomography for Assessment of Crohn's Disease Activity. N Engl J Med. 2017 Mar 30;376(13):1292-1294.

Knieling F. Light and sound - emerging imaging techniques for inflammatory bowel disease. World J Gastroenterol. 2016 Jul 7;22(25):5642-54.

Waldner MJ, Knieling F. et al Multispectral Optoacoustic Tomography in Crohn's Disease: Noninvasive Imaging of Disease Activity. Gastroenterology. 2016 Aug;151(2):238-40.

Regensburger A. et al. Detection of collagens by multispectral optoacoustic tomography as an imaging biomarker for Duchenne muscular dystrophy. Nat Med. 2019 Dec 11 14:9809-9821.

Content is confidential.

7. Study objective

Primary study objective:

Comparison of the optoacoustic spectrum determined by MSOT in patients with PD compared to patients with DMD and healthy volunteers, generating a new biomarker for disease monitoring in PD.

Secondary study objectives:

- Comparison of the quantitative glycogen signal fraction determined by MSOT in patients with PD compared to to patients with DMD and healthy volunteers
- Comparison of the quantitative lipid signal fraction determined by MSOT in patients with PD compared to to patients with DMD and healthy volunteers
- Comparison of the quantitative fraction of collagen signal determined by MSOT in patients with PD compared to to patients with DMD and healthy volunteers
- Comparison of the quantitative fraction of hemo-/myoglobin signal determined by MSOT in patients with PD compared to to patients with DMD and healthy volunteers
- Comparison of the quantitative fraction of oxygenated/deoxygenated hemoglobin determined by MSOT in patients with PD compared to to patients with DMD and healthy volunteers

- Correlation of glycogen content determined with MSOT with disease duration/patient age
- Correlation of lipid content determined with MSOT with disease duration/patient age
- Correlation of collagen determined by MSOT with disease duration/patient age
- Correlation of haemoglobin/myoglobin content determined by MSOT with duration of disease/patient age
- Correlation of oxygenated/deoxygenated hemoglobin determined by MSOT with duration of disease / patient age

- Correlation of glycogen content determined with MSOT with R-Pact scale
- Correlation of lipid content determined with MSOT with R-PAct scale
- Correlation of collagen determined by MSOT with R-PAct scale
- Correlation of haemoglobin/myoglobin content determined by MSOT with R-PAct scale
- Correlation of oxygenated/deoxygenated hemoglobin determined by MSOT with R-PAct scale

Content is confidential.

- Correlation of glycogen content determined with MSOT with age-related functional muscle tests (Hammersmith Infant Neurological Examination (HINE)/The Children's Hospital of Philadelphia Infant Test of Neuromuscular Disorders (CHOP Intend)/expanded Hammersmith functional motor scale (HFMSE)/ Revised Upper Limb Module (RULM)/6-Minute-Walk Test (6-MWT)/Time-to-go-up-and-go-test/MRC Muscle Strength Grades)
- Correlation of lipid determined with MSOT with age-dependent functional muscle tests (HINE/CHOP Intend/HFMSE/RULM /6-MWT/Time-to-get-up-and-go/MRC)
- Correlation of collagen determined with MSOT with age-dependent functional muscle tests (HINE/CHOP Intend/HFMSE/RULM/6-MWT/Time-to-get-up-and-go/MRC)
- Correlation of hemo-/myoglobin content determined with MSOT with age-dependent functional muscle tests (HINE/CHOP Intend/HFMSE/RULM/6-MWT/Time-to-get-up-and-go/MRC)
- Correlation of oxygenated/deoxygenated hemoglobin determined with MSOT with age-related functional muscle tests (HINE/CHOP Intend/ HFMSE/RULM /6-MWT/Time-to-get-up-and-go/MRC)

- Correlation of glycogen content determined with MSOT with B-mode ultrasound (Heckmatt scale/Echogenity/Gray Scale Level)
- Correlation of lipid determined with MSOT with B-mode ultrasound (Heckmatt scale/Echogenity/Gray Scale Level)
- Correlation of collagen determined with B-mode ultrasound (Heckmatt scale/Echogenity/Gray Scale Level)
- Correlation of hemo-/myoglobin content determined with MSOT with B-mode ultrasound (Heckmatt scale/Echogenity/Gray Scale Level)
- Correlation of oxygenated/deoxygenated hemoglobin determined with B-mode ultrasound (Heckmatt scale/Echogenity/Gray Scale Level)

- Correlation of glycogen content determined with MSOT with respiratory function tests (Spirometry)
- Correlation of lipid content determined with MSOT with respiratory function tests (Spirometry)
- Correlation of collagen determined by MSOT with respiratory function tests (Spirometry)
- Correlation of haemoglobin/myoglobin content determined by MSOT with respiratory function tests (Spirometry)

Content is confidential.

- Correlation of oxygenated/deoxygenated hemoglobin determined by MSOT with respiratory function tests (Spirometry)

- Measurement of signal differences in right / left comparison

Hypotheses:

- The optoacoustic spectrum of muscles of patients with PD is different compared to patients with DMD and healthy volunteers
- The optoacoustic spectrum of the liver of patients with PD is different compared to patients with DMD and healthy volunteers
- The quantitative fraction of glycogen signal in muscles determined by MSOT differs in patients with PD compared to patients with DMD and healthy volunteers
- The quantitative fraction of lipid signal in muscles determined by MSOT differs in patients with PD compared patients with DMD and healthy volunteers
- The quantitative fraction of collagen signal in muscles determined by MSOT differs in patients with PD compared to patients with DMD and healthy volunteers
- The quantitative fraction of hemo-/myoglobin in muscles determined by MSOT differs in patients with PD compared to patients with DMD and healthy volunteers
- The quantitative fraction of oxygenated/deoxygenated hemoglobin in muscles determined by MSOT differs in patients with PD compared to patients with DMD and healthy volunteers
- There is a correlation between MSOT derived glycogen signal and clinical status of patients with PD
- There are no side differences in patients with PD and patients with DMD as well as healthy volunteers

Study type

Since no data exists so far to support the hypothesis of this study, it is an explorative study / pilot study.

8. Endpoint parameters

All measurements with MSOT are performed over the paraspinal musculature, Musculus trapezius as well as proximal and distal limb muscles in a right-left comparison (leg proximal: Musculus quadriceps, distal: Musculus triceps surae; arm proximal: Musculus biceps, distal: Forearm flexors) in healthy subjects compared to patients with PD and patients with DMD. Additionally, we will perform measurements of the liver with MSOT.

Content is confidential.

Primary endpoint:
Optoacoustic Absorption Spectrum of Muscle and liver in PD.
This target is measured non-invasively by MSOT.

Secondary endpoints:
Quantitative glycogen signal (in arbitrary units)
Quantitative lipid signal (in arbitrary units)
Quantitative collagen signal (in arbitrary units)
Quantitative hemo/myoglobin signal (in arbitrary units)
Muscle oxygenation (in %)
These target values are measured non-invasively by MSOT.

B-mode ultrasound
- Heckmatt scale
- Echogenitiy
- Gray Scale Level

R-PaAt scale
- Summed raw score

Respiratory function test
- FVC
- FEV1

Correlation of glycogen detected by MSOT with clinical scores for determining muscle strength consisting of:
Patients <2 years and patients > 2 years with inability to sit:

- Hammersmith Infant Neurological Examination (HINE)/The Children's Hospital of Philadelphia Infant Test of Neuromuscular Disorders (CHOP Intend)

at age >2 years and ability to sit in a wheelchair additionally:

- Revised Upper Limb Module (RULM)

Patients >2 years and sitting ability
- Expanded Hammersmith functional motor scale (HFMSE)/ Revised Upper Limb Module (RULM)

in case of ability to walk additionally:

Content is confidential.

- 6-minute walking test
- Time-to-get-up-and-go-test

Patients >3 years and walking ability

- Expanded Hammersmith functional motor scale (HFMSE)/ Revised Upper Limb Module (RULM)
- 6-minute walking test
- Time-to-get-up-and-go-test

All age groups

- MRC Muscle Strength Grades

<2 years	> 2 years with inability to sit	> 2 years with ability to sit in a wheelchair	> 2 years and ability to sit	> 3 years and ability to walk
HINE Section 2	HINE Section 2	HINE Section 2		
CHOP Intend	CHOP Intend	CHOP Intend		
		(HFMSE)	HFMSE	HFMSE
		RULM	RULM	RULM
				6MWT
				TTGUAG
MRC	MRC	MRC	MRC	MRC

These target values are clinically determined at presentation (electronic patient record, physician letters):

Age

Sex

Weight

Skin color

Ethnic Background

Disease duration

Current medication

Results of last biopsy (if available)

Results of last MRI (if available)

Study Parameters	PD	HV	DMD
MSOT	X	X	X
-'Glycogen'	X	X	X
-Lipid	X	X	X
-Collagen	X	X	X
-Hemoglobin oxygenated	X	X	X
-Hemoglobin deoxygenated	X	X	X
Ultrasound	X	X	X
-Heckmatt	X	X	X
-Echogenity	X	X	X
-Muscle texture	X	X	X
-Grey scale level	X	X	X
R-Pact scale (≥16y)	X	X	
Respiratory function test	X	X	
-FVC	X	X	
-FEV1	X	X	
Muscle tests	X	X	
-HINE	X	X	
-CHOP Intend	X	X	
-HFMSE	X	X	
-RULM	X	X	
-6-MWT	X	X	
-Time-to-get-up-and-go	X	X	
-MRC	X	X	
**Patient History**			
Confirmed Disease	X<br>Type:<br>Crim:	X	X
Diagnostics:	X		X
-Genetics	X		X
-Dried Blood Spot	X		
-Urinary Glc 4	X		
-Lymphozytes	X		
Muscle biopsy	X		X
Neurological tests:	X		X
-EMG	X		X
-MRI	X		X
-Sleep Study	X		X
Lab values	X		X
-CK	X		X
-Liver enzymes	X		X
Sitting ability	X		X
Walking ability	X		X
Ventilation	X		X
Medication			

Content is confidential.

9. Study design

Monocentric/Multicentric

This is a multicentric study with matched collectives (age, gender)/

Study arms: intervention/control

Interventions are not planned. A comparison is made between patients with PD, patients with DMD and healthy volunteers. The study procedure is identical for PD patients and healthy volunteers. DMD patients receive MSOT only. Healthy volunteers are used as controls (no muscular pathology, no excessive intramuscular lipids/collagen, no excessive glykogen), DMD patients as pathologic controls (muscular pathology, intramuscular lipids/collagen, no excessive glycogen) versus PD patients (muscular pathology, intramuscular lipids/collagen, excessive glycogen).

Randomization

Randomization is not planned. The allocation to the groups is based on the known diagnosis (PD, other muscular pathology) and controls (subjects).

Blinding

Blinding for the examination is not possible due to the possible clear clinical appearance. Blinding takes place during the measurement and and evaluation of the data. Blinding of the patients/controls is not necessary.

Content is confidential.

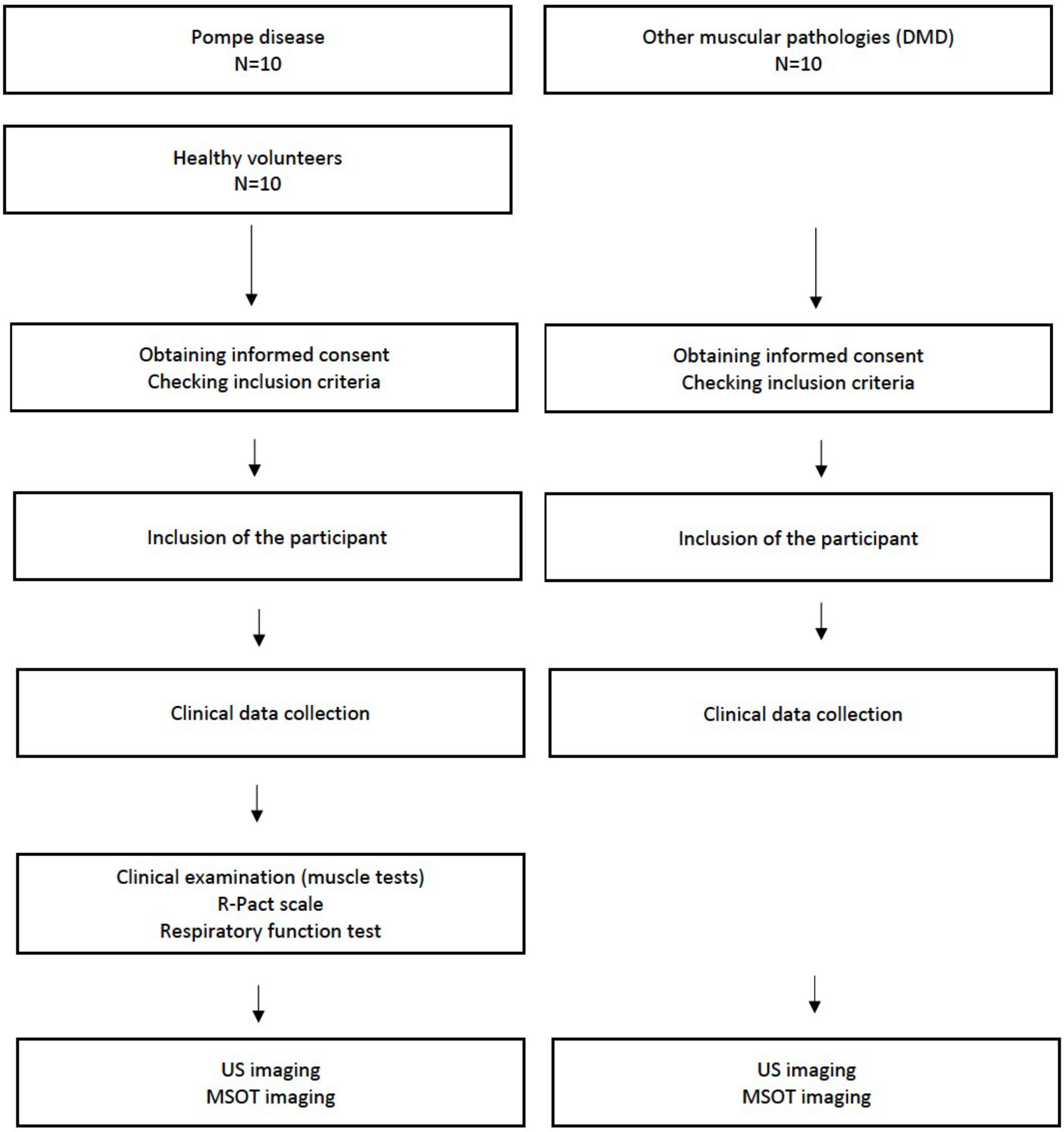

Content is confidential.

10. Study population

Inclusion and exclusion criteria

Inclusion criteria:
PD patients (IOPD and LOPD)

- Confirmed diagnosis of Pompe disease
- From birth
- Independent from current therapy

DMD patients

- Genetically confirmed DMD diagnosis
- From birth
- Independent from current therapy

Healthy controls

- From birth, matched (age, gender) to PD collective

Exclusion criteria

PD patients

- Pregnancy
- Tattoo on skin to be examined

DMD patients

- Pregnancy
- Tattoo on skin to be examined

Healthy controls

- Anamnestic of other signs of myopathy or liver disease
- Pregnancy
- Tattoo on skin to be examined

Patient/control number

As this is a pilot study, an exact case number calculation is not possible. It is planned to study a total of 10 patients with PD, 10 patients with DMD and 10 healthy controls.

Content is confidential.

Recruitment routes and measures

Patients (and parents) are informed about the possibility of participating in the study in the context of an elective presentation at the Clinic for Pediatrics and Adolescent Medicine (Neuropediatrics) and the Clinic for Neurology at the University Hospital in Erlangen, Germany as well as the the Clinic for Pediatrics and Adolescent Medicine (Neuropediatrics) in Gießen, Germany. Additional recruitment options include the Clinic Rummelsberg, Schwarzenbruck, Germany, the German Society for muscular diseases (DGM), the treatNMD network and the international Pompe Association. If the patient is willing to participate, he/she will be fully informed about the aims and methods (especially about the scientific/explorative character of the study), benefit and risk and revocability of the study participation. Patients in childhood and adolescence will also be informed and educated about the study and its procedure according to their age.

Healthy volunteers are recruited in the outpatient department of our Clinic for Pediatrics and Adolescent Medicine as well as the Clinic for Neurology at the University Hospital in Erlangen, Germany.

Acutely ill or unstable patients are not recruited. In the preliminary phase, volunteers are parallelised with the PD collective in terms of age and sex.

11. Study flow

Procedure for informing about and obtaining consent

Patients or test persons can only be included in the study after a written consent has been given. The written declaration of consent requires oral and written information of the patients/test persons as well as their parents or legal guardians about goals and methods (incl. scientific-explorative character of the study), benefit and risk as well as revocation of participation in the study. Children and adolescents are informed by means of age-appropriate, comprehensible patient information sheets. By giving their written consent, the patients/test persons and their parents/guardians declare that they agree to the collection and storage of study-relevant data and their verification by monitoring or authorities. The study participant must be clearly informed that the declaration of consent can be withdrawn at any time and without any disadvantage. Furthermore, all study participants/test persons and parents/guardians are informed that this study is a purely scientific study without any current diagnostic or therapeutic benefit.

The original of the declaration of consent will be kept in the study folder at the place of study. The patient/control and the parents/guardian receive a copy of the patient information and

declaration of consent. The patient information and the consent form are attached to this protocol.

Measurements

After informing the patient / control and parent / guardian and obtaining consent, clinical scores are collected to assess muscle strength according to age (Hammersmith Infant Neurological Examination (HINE)/ expanded Hammersmith functional motor scale (HFMSE)/ The Children's Hospital of Philadelphia Infant Test of Neuromuscular Disorders (CHOP Intend)/ Revised upper Limb Module (RULM)/6-minute walking test (6-MWT)/ Time-to-get-up-and-go-test/ MRC Muscle test). Additionally, R-Pact scale will be collected and respiratory function tests will be performed. Muscle tests, R-PAct scale and respiratory function test will not be performed n the DMD patient group, as these patients do primarily serve as a reference for the MSOT imaging.

Subsequently, MSOT imaging is performed on 6 anatomical regions in all study participants: Paraspinal muscles, Musculus trapezius, upper/lower arm and upper/lower leg on predefined muscle groups. (Leg proximal: Musculus quandriceps, distal: Musculus triceps surae; arm proximal: Musculus biceps, distal: Forearm flexors). The examination is analogous to sonography over the corresponding skin layers without further invasive procedures. The anatomical region can be localized by means of built-in B-image sonography; the corresponding optoacoustic signals can then be conducted. The duration per anatomical region is limited to 5 minutes; this corresponds to a maximum of 10 minutes for the parasinal muscles and Musculus trapezius, a maximum of 20 minutes for both upper extremities and 20 minutes for both lower extremities and 10 minutes for the examination of the liver. Patients can remain in a relaxed posture during the examination, without the need for breathing maneuvers or similar assistance.

Recording of target parameters

- Determination of routine data (duration of disease, current medication)
- Clinical evaluation of muscle strength by HINE/HFMSE/CHOP Intend/RULM/6-MWT/ Time-to-get-up-and-go/ MRC muscle test
- Assessment of R-Pact scale
- Performance of respiratory function test
- Non-invasive in-vivo measurement of glycogen, lipid, collagen, myo/hemoglobin content and oxygenation by MSOT

Content is confidential.

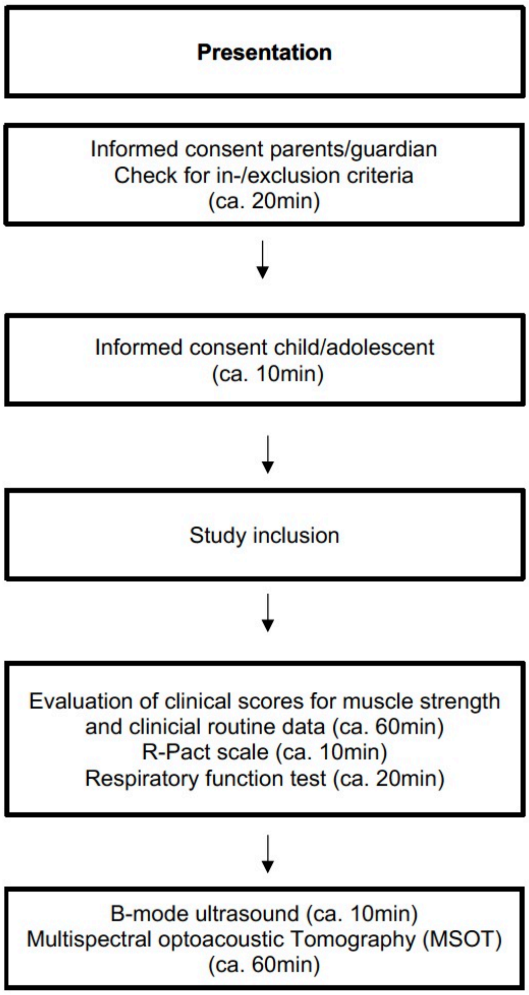

Time schedule and study duration for the individual patient/control

For the individual patient, the duration of the study participation is 190 minutes. Approximately 30 minutes are spent on education for study participants and parents/guardians, 60 minutes on clinical (routine) testing of muscle strength, 10 minutes on R-Pact scale, 20min on respiratory function test, 10 minutes for B-mode ultrasound and 60 minutes for the MSOT examination. For DMD patients, the total allocated time is 100min (consent, imaging).

Total duration of study

Depending on the number of patients, the expected total duration of the study up to the inclusion of the last patient is 8 months.

12. Benefit-risk-assessment

All study-related risks (related to MSOT)

Based on the classification criteria for medical devices (Directive 93/42/EEC, Annex IX), the iThera Medical optoacoustic system complies with Class IIa laser systems:

- Active diagnostic device
- Non-invasive
- Temporary use (<60 min)

The used system has CE-certification (TÜV Süd, 02.05.2021, type designation according to imprint: MSOT Acuity Echo). A conformity assessment procedure to expand the use of the system is not intended or planned by the manufacturer at this time. It is therefore a purely scientific pilot study. There is no dependency relationship with the manufacturer; all diagnostic and analytical procedures are available to the study directors on site. The cooperation with the company will be regulated in a separate contract drawn up by the legal department before the start of the study.

Compliance with energy limits

The laser safety and maximum permitted radiation dose for irradiation with laser pulses is regulated in the ANSI and IEC 60825 laser standards. The MSOT system complies with these standards and remains below the MPE (maximum permissible exposure) limits for skin irradiation and is therefore considered safe.

Temperature increases in tissue

Optoacoustic imaging does not result in a significant increase in tissue temperature. The absorption of a laser pulse in tissue results in a local transient temperature increase of a few millikelvin. Depending on the duration of the examination and the patient's skin type, there is typically a temperature increase in the range of less than one degree Kelvin.

Histological changes in tissue

Histological changes in the target tissue and surrounding structures are neither expected nor have they been observed in previous preclinical and clinical studies.

Slight, reversible redness or warming is only to be expected in sensitive skin.

Content is confidential.

Such impairment of the patient can be noticed by the test person or physician at any time; the examination can then be interrupted or discontinued. In any case, no irreversible damage is to be expected.

In principle, the near-infrared light used in the MSOT can lead to retinal damage if the eye is irradiated. To prevent this, the participants and examiners will wear appropriate laser safety glasses during the examination.

Since the data obtained will not be used to interpret findings, there is no risk of possible misdiagnosis or incorrect display of data in this exploratory pilot study.

There are no other risks within the scope of this study, nor have they been described based on our own preliminary data.

13. Biometrics

Exploratory study: Explanation of the statistical methodology, justification of the chosen case number

Case number calculation

As this is a pilot study and no information is available on the expected differences between the different groups, no case number calculation was carried out. The number of cases given represents an estimate or is within reasonable limits for a pilot study.

Statistical Methodology

Continuous variables are given as mean values with standard deviation, categorical variables as numbers with percentages where appropriate. The MSOT parameters are compared using a two-sided, unpaired t-test with equal deviations. If the standard deviation is not equal, Welch correction is applied where appropriate. ROC (Receiver Operator Characteristics) analyses between healthy and sick persons are also planned. Genetics serves as the gold standard. Correlations are indicated by the Pearson coefficient. All statistical tests will be double-sided and a p-value of <0.05 is considered statistically significant. All analyses are performed with GraphPad Prism (version 7.00 or later, GraphPad Software, La Jolla, CA, USA), RStudio (version 1.1.456 or later, RStudio Inc., Boston, MA, USA) or IBM SPSS Statistics (version 24 or later, IBM Corp., Armonk, NY, USA).

Content is confidential.

14. Data management and protection

All raw data, such as patient records, represent source documents. Their availability is ensured for routine monitoring. The participation of the individual patients or test persons in the study is documented; the study director maintains a separate list to identify the all screened and participating patients. This list contained the names and date of birth as well as the examination date and pseudonymization abbreviations of the patients and test persons. The study director is responsible for the quality of data collection and storage. The data storage (total data) is carried out on computers or specially designed network drives of the University Hospital Erlangen. The raw imaging data (no patient-related data) is stored on specially designated servers of the company iThera Medical GmbH.

Pseudonymisation

Prior to a scientific analysis of the materials and data of this study, all information will be pseudonymised in accordance with the guidelines of the Federal Data Protection Act.

Data transfer

Data sharing in this study is intended solely for the MSOT raw data. The company iThera Medical GmbH will work with this data to ensure adequate recording quality and to develop algorithms for evaluation. The data will only be transferred pseudonymized on encrypted physical drives. The data will not be used for a later approval of the prototype used. The cooperation will be explicitly regulated by the legal department before the study starts (a supplementary agreement to the service contract can be found in the appendix).

The study results can be published anonymously, but it will not be possible to draw conclusions about the identity of the participating persons. The data will be kept for 10 years and destroyed afterwards.

Revocation, data deletion

If the declaration of consent is revoked, data collected up to this point can be taken into account. The patient has the right to demand their destruction, provided that legal provisions do not conflict with the destruction.

15. Biomaterial handling

No biomaterials are obtained.

16. Individual paticipant insurance

The participants of the study are insured through the group contract of the CCS Erlangen.

17. Signatures

Dr. med. Ferdinand Knieling
Study director

Prof. Dr. med. Regina Trollmann
Study director

Prof. Dr. med. Joachim Wölfle
Clinic director

3. Study Protocol (Amended Version)

Study protocol

SPOT_PD

Multi**sp**ectral **O**ptoacoustic **T**omography for Translational Molecular Imaging in **P**ompe **D**isease

Content is confidential.

1. Table of contents

1. Table of contents 2
2. Study titel, version number, version date 3
3. Project summary 4
4. Project summary (German) 5
5. Responsabilities 6
6. Scientific background 7
7. Study objective 12
8. Endpoint parameters 15
9. Study design 18
10. Study population 20
11. Study flow 21
12. Benefit-risk-assessment 24
13. Biometrics 27
14. Data management and protection 27
15. Biomaterial handling 28
16. Individual paticipant insurance 28
17. Signatures 29

Content is confidential.

2. Study titel, version number, version date

Study title

Multispectral Optoacoustic Tomography for Translational Molecular Imaging in Pompe Disease

Version number

Version 1.3

Version date

30.05.2023

Protocol versions

Date	Version	Status	Changes
03.01.2020	1.0	Outline	
11.06.2021	1.1	Final	
05.08.2021	1.2	Revision	Minor arm excluded
02.03.2022	1.3	Amendment	MRI, UGAP included
30.05.2023	1.4	Amendment	DMD arm canceled

Content is confidential.

3. Project summary

In patients with Pompe disease (PD) a progressive abnormal lysosomal glycogen storage in muscle tissue leads to impaired muscle function and to degeneration of muscle fibers. Children and adults with PD present with limb-girdle muscular weakness, diaphragm weakness and impaired breathing ability. Further, patients with classic infantile PD suffer from hypertrophic cardiomyopathy. To date, the muscle pathology and the extent of the disease can be assessed using invasive techniques (e.g., muscle biopsies) or imaging (e.g., MRI). These techniques are time consuming, and especially in young patients, require anesthesia, which increases the acute risk of respiratory failure.

Multispectral optoacoustic tomography (MSOT) allows the detection of specific endogenous chromophores like collagen, myoglobin or hemoglobin by using a non-invasive approach comparable to conventional ultrasound. Instead of sound waves, MSOT illuminates tissue with near-infrared light of transient energy, which is absorbed and results in thermo-elastic expansion of certain molecules. This expansion generates ultrasound waves that are detected by the same device. Multispectral illumination and unmixing then allows the precise localisation and quantification of muscle-specific subcellular structures. MSOT has already been demonstrated the potential to visualize the muscular structure and the clinical extent of muscular disease in patients with Duchenne muscle dystrophy and differentiates those patients from healthy volunteers.

The aim of the study is to establish glycogen as a novel PD-specific imaging target using MSOT-imaging. Furthermore, we will apply MSOT-imaging to identify a PD-specific muscle pathology-signature by quantification of already established targets (collagen, myoglobin, hemoglobin, glycogen if applicable). This signature will aid in differentiating PD from other muscular pathologies and healthy volunteers and will ultimately serve as a potential non-invasive monitoring biomarker.

Content is confidential.

4. Project summary (German)

Bei Patienten mit Morbus Pompe (PD) führt eine fortschreitende abnorme lysosomale Glykogenspeicherung im Muskelgewebe zu einer Beeinträchtigung der Muskelfunktion und zur Degeneration von Muskelfasern. Kinder und Erwachsene mit Morbus Pompe zeigen eine Schwäche der Gliedergürtelmuskulatur, eine Zwerchfellschwäche und eine Beeinträchtigung der Atemfunktion. Außerdem leiden Patienten mit klassischer infantiler PD an einer hypertrophen Kardiomyopathie. Bislang können die Muskelpathologie und das Ausmaß der Erkrankung mit invasiven Techniken (z. B. Muskelbiopsien) oder bildgebenden Verfahren (z. B. MRT) beurteilt werden. Diese Techniken sind zeitaufwendig und erfordern insbesondere bei jungen Patienten eine Anästhesie, was das akute Risiko eines Atemversagens erhöht.

Die multispektrale optoakustische Tomographie (MSOT) ermöglicht den Nachweis spezifischer körpereigener Chromophore wie Kollagen, Myoglobin oder Hämoglobin durch einen nicht-invasiven Ansatz, der mit konventionellem Ultraschall vergleichbar ist. Anstelle von Schallwellen beleuchtet MSOT das Gewebe mit Nahinfrarotlicht von transienter Energie, das absorbiert wird und zu einer thermoelastischen Ausdehnung bestimmter Moleküle führt. Diese Ausdehnung erzeugt Ultraschallwellen, die von demselben Gerät detektiert werden. Die multispektrale Beleuchtung und Entmischung ermöglichen dann die präzise Lokalisierung und Quantifizierung von muskelspezifischen subzellulären Strukturen. MSOT hat bereits das Potential gezeigt, die Muskelstruktur und das klinische Ausmaß der Muskelerkrankung bei Patienten mit Duchenne-Muskeldystrophie sichtbar zu machen und diese Patienten von gesunden Freiwilligen zu unterscheiden.

Ziel der Studie ist es, Glykogen als neuartiges PD-spezifisches Bildgebungsziel mittels MSOT-Bildgebung zu etablieren. Darüber hinaus werden wir MSOT anwenden, um eine PD-spezifische Muskelpathologie-Signatur durch Quantifizierung bereits etablierter Targets (Kollagen, Myoglobin, Hämoglobin, Glykogen, falls zutreffend) zu identifizieren. Diese Signatur wird helfen, PD von anderen muskulären Pathologien und gesunden Probanden zu unterscheiden und wird letztendlich möglicherweise als potenzieller nicht-invasiver Überwachungs-Biomarker dienen.

Content is confidential.

5. Responsabilities

Study director

Dr. Ferdinand Knieling

Pediatric Experimental and Translational Imaging Laboratory (PETI_Lab)

Department of Pediatrics
University Hospital Erlangen
Loschgestr. 15
91054 Erlangen
Germany
Tel. 09131 8533118
Mail: ferdinand.knieling@uk-erlangen.de

Prof. Dr. Regina Trollmann

Head of the Department of Neuropediatrics

Department of Pediatrics
University Hospital Erlangen
Loschgestr. 15
91054 Erlangen
Germany
Tel.: 09131 85 33753
Mail: regina.trollmann@uk-erlangen.de

Sponsoring

Investigator-initated trial, funding by Sanofi Genzyme

6. Scientific background

Pompe disease (PD) is a rare, autosomal-recessive disorder caused by deficiency of the lysosomal acid alpha-glucosidase enzyme (GAA), leading to generalized build-up of glycogen, especially in the heart, muscle, liver and nervous system (Pompe, 1932, Hers, 1963). Among the glycogen storage diseases, PD is the only one with a defect in lysosomal metabolism.

PD is considered as a progressive disease with variation by age of onset, severity of organ involvement and degree of myopathy. This great phenotypic variability has led to the creation of types based on the age of onset and degree of organ involvement. They all have in common, that symptoms of affected patients are expected to worsen over time if left untreated. The classification is generally based on the age of onset as infantile (infantile onset Pompe disease, IOPD) when it presents during the first 12 months of life and late-onset (LOPD) when first symptoms appear after 12 months of age. If cardiomyopathy is present, IODP is generally referred to as classic Pompe disease (however there may be variably classification in the literature with the infantile or childhood forms). Clinically, infants with classic PD present during the first few months of life with rapidly progressive disease characterized by prominent hypertrophic cardiomyopathy, hepatomegaly, hypotonia, generalized muscle weakness, macroglossia, feeding difficulties and respiratory insufficiency. Mortality rate is high by one year of age if untreated (Kishnani 2004, 2006). Patients with non-classic PD will usually present within the first year of life with motor developmental delay and weakness, but without clinically relevant cardiac involvement. The rate of clinical progression is slower in these children and without treatment, death will usually occur in childhood as a result of respiratory insufficiency (Hers 1963, Slonim 2000). LODP include childhood and adult-onset PD. These patients generally present with slowly progressive limb girdle type weakness and respiratory insufficiency without significant cardiomyopathy (Cupler 2012, Preisler 2013). The diagnosis of PD is usually established by the typical clinical presentation, followed by confirmation of GAA deficiency in dried blood spots, e.g. through new-born screening (Bodamer, 2017). Further (confirmatory) methods include GAA activity measurement in lymphocytes, muscle or skin fibroblasts, as well as GAA mutation testing (Van den Hout, 2003, Case, 2012). All of them are invasive techniques. Early identification is important as it will likely significantly improve the outcome for all patients with PD as treatment can be initiated earlier. Treating the underlying cause of PD involves the replacement of the missing enzyme GAA via enzyme replacement therapy (ERT) with alglucosidase alfa (recombinant human GAA, rhGAA). Currently, this is the only specific treatment approved for PD. In classical IOPD, treatment significantly lengthens survival and improves motor development and respiratory and cardiac function (Kishnani, 2007). The sooner ERT begins, the better are the results (Chien, 2013). With ERT being one

Content is confidential.

very important aspect of care, patients will also need a multidisciplinary approach to ensure that all aspects of the disease are addressed.

Regardless of age of onset and severity, all patients with PD should be monitored prospectively (Kishnani, 2006, Bembi, 2008). However, there is lack of standardization across centers. A variety of clinical evaluations and tests are currently used for monitoring Pompe's disease, which may include laboratory tests including CK, AST, ALT, and LDH, cardiologic tests including electrocardiogram and echocardiogram and respiratory tests including sleep studies and breathing tests to measure lung capacity. To quantify muscle involvement electromyography is an option as well as clinical tests including 6 minutes walking test or timed to up and go test. Muscle MRI of affected patients often show fatty degeneration of muscles. One study showed that muscle MRI correlates with muscle function in patients with adult-onset Pompe disease (Figueroa-Bonaparte, 2016). Another study suggested that muscle imaging data in late-onset Pompe disease reveal a correlation between the pre-existing degree of lipomatous muscle alterations and the efficacy of long-term enzyme replacement therapy (Gruhn, 2015). For small children, however, there is always a need for sedation for MRI's, limiting its use. Therefore, ultrasound is another option to examine children's muscles.

At the moment there are no prospective biomarkers available to detect muscle degeneration at an early age and/or to follow up disease progression or ERT-treated patients. Within the last years our multidisciplinary research team (Medical Department 1, Department of Pediatrics and Adolescent Medicine, University Hospital Erlangen) published a novel non-invasive imaging modality to be able to detect subcellular tissue composition in vivo. Multi-spectral optoacoustic tomography (MSOT), an imaging technology comparable to ultrasound, allows quantitative imaging in patients of all ages (including the non-sedated child).

Content is confidential.

For MSOT, similar to a conventional ultrasound, an ultrasound transducer is positioned on the patient's skin. Instead of sound waves, MSOT illuminates tissue with light of transient energy, typically near-infrared laser light pulses, which are absorbed by the tissue, resulting in thermo-elastic expansion. This expansion gives rise to ultrasound waves that are detected by the same device. Studies have already shown that MSOT-based assessment of hemoglobin levels in the intestinal wall has the potential for Assessment of Crohn's Disease Activity (Knieling 2017, Waldner 2016). With a newly configurated device (Acuity Echo, iThera Medical GmbH, Munich, custom-built platform) an extended spectrum of laser light can be used, allowing the detection of not only hemoglobin but also further biomarkers like collagen or lipid. In our recent study, we were able to show the molecular composition of muscles in Duchenne muscle dystrophy via MSOT suggesting non-invasive measured collagen content as a novel biomarker for disease severity (Regensburger, 2019). In this study we want to establish glycogen as a novel PD-specific imaging target using MSOT-imaging.

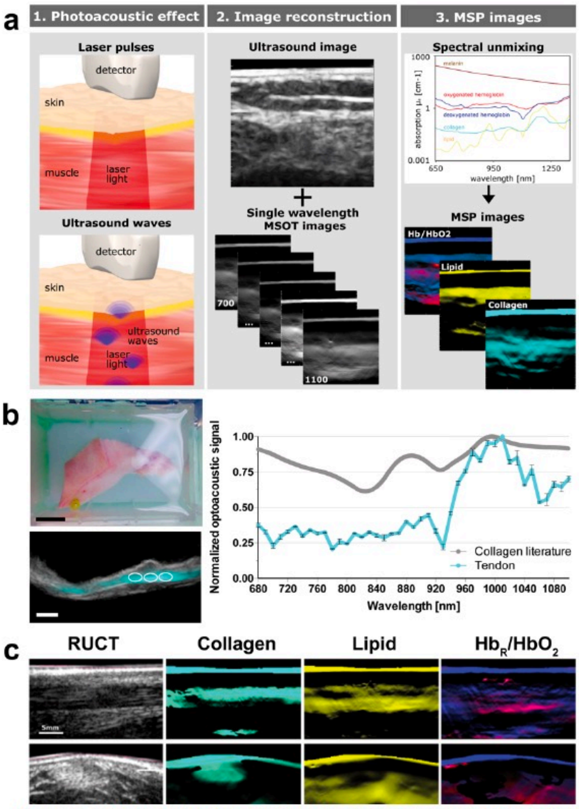

Figure 1 - MSOT principle (a), experimental preliminary work (b), and first time representation of collagen in vivo (c)

Figures 1 and 2 show exemplary MSOT images. In the light of the progression and the sometimes severe course of PD, this method would for the first time allow a non-invasive and quantitative determination of the molecular composition of muscle tissue. In this first pilot study in patients with PD, it will now be investigated whether the differences in the muscle composition of healthy volunteers and PD patients can be quantified and whether this could simultaneously be used as marker during ERT-therapy. In the future, this could generate a completely new, non-invasive method to evaluate endogenous biomarkers for therapy response.

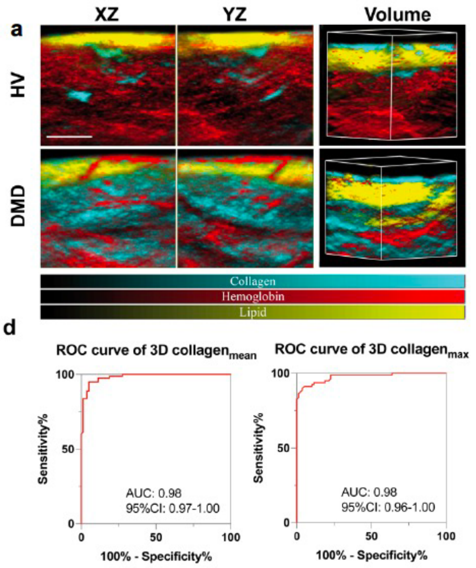

Figure 2 - Feasibility of 3D MSOT image (a) and diagnostic quality (b).

Literature

Pompe JC. Over idiopatische hypertrophie van het hart. Ned Tijdschr Geneeskd. 1932; 76; 304-12.

Hers HG. Alpha-Glucsidase deficiency in generalized glycogen storage diease (Pompe's disease), Biochem J. 1963, 86, 11-6.

Kishnani PS, Howell RR. Pompe disease in infants and children. J Pediatr. 2004;144(Suppl 5):S35-43.

Kishnani PS, Hwu WL, Mandel H, Nicolino M, Yong F, Corzo D. A retrospective, multinational, multicenter study on the natural history of infantile-onset Pompe disease. J Pediatr. 2006;148(5): 671-6.

Hers HG. Alpha-Glucosidase deficiency in generalized glycogen storage disease (Pompe's disease) Biochem J. 1963;86:11–16.

Slonim AE, Bulone L, Ritz S, Goldberg T, et al. Identification of two subtypes of infantile acid maltase deficiency. J Pediatr. 2000;137:283–285.

Cupler EJ, Consensus treatment recommendations for late-onset Pompe disease. Muscle Nerve. 2012, 45:319-33.

Preisler N. Late-onset Pompe disease is prevalent in unclassified limb-girdl muscular dystrophies. Mol Genet Metab. 2013, 110:287-9.

Bodamer OA. Newborn Screening for Pompe Disease. Pediatrics, 2017. Jul; 140 (Suppl 1) S4-S13

Van den Hout HM. The natural course of infantile Pompe's disease: 20 original cases compared with 133 cases from the literature. Pediatrics. 2003, 112.332-40.
Case LE. Infantile Pompe disease on ERT. Update on clinical presentation, musculoskeletal management and exercise considerations. Am J Med Genet C Semin Med Genet. 2012. 160C: 69-79.

Content is confidential.

Lumyzyme (alglucosidase alfa) for injection, for intravenous use (prescribing information). Cambridge, MA: Genzyme Corporation; 2014.

Myozyme (alglucosidase alfa) for injection, for intravenous use (prescribing information). Cambridge, MA: Genzyme Corporation; 2014.

Kishani PS, Recombinant human Recombinant human acid [alpha]-glucosidase: major clinical benefits in infantile-onset Pompe disease. Neurology, 2007, Jan 9; 68 82); 99-109.

Chien YH, Hwu WL, Lee NC. Pompe disease: early diagnosis and early treatment make a difference. Pediatr Neonatol. 2013;54(4):219–227

Kishnani PS, Steiner RD, Bali D,et al. Pompe disease diagnosis and management guideline [published correction appears in Genet Med. 2006;8(6):382]. Genet Med. 2006;8(5):267–288

Bembi B, Cerini E, Danesino C,et al. Management and treatment of glycogenosis type II. Neurology. 2008;71(23 suppl 2):S12–S36

Figueroa-Bonaparte S, Muscle MRI Findings in Childhood/Adult Onset Pompe Disease Correlate with Muscle Function, PLoS One. 2016; 11(10): e0163493.

Gruhn KM, Muscle imaging data in late-onset Pompe disease reveal a correlation between the pre-existing degree of lipomatous muscle alterations and the efficacy of long-term enzyme replacement therapy. Mol Genet Metab Rep. 2015 Jun; 3: 58–64.

Knieling F. Multispectral Optoacoustic Tomography for Assessment of Crohn's Disease Activity. N Engl J Med. 2017 Mar 30;376(13):1292-1294.

Knieling F. Light and sound - emerging imaging techniques for inflammatory bowel disease. World J Gastroenterol. 2016 Jul 7;22(25):5642-54.

Waldner MJ, Knieling F. et al Multispectral Optoacoustic Tomography in Crohn's Disease: Noninvasive Imaging of Disease Activity. Gastroenterology. 2016 Aug;151(2):238-40.

Regensburger A. et al. Detection of collagens by multispectral optoacoustic tomography as an imaging biomarker for Duchenne muscular dystrophy. Nat Med. 2019 Dec 11 14:9809-9821.

Content is confidential.

7. Study objective

Primary study objective:

Comparison of the optoacoustic spectrum determined by MSOT in patients with PD compared to healthy volunteers, generating a new biomarker for disease monitoring in PD.

Secondary study objectives:

- Comparison of the quantitative glycogen signal fraction determined by MSOT in patients with PD compared to healthy volunteers
- Comparison of the quantitative lipid signal fraction determined by MSOT in patients with PD compared to healthy volunteers
- Comparison of the quantitative fraction of collagen signal determined by MSOT in patients with PD compared to healthy volunteers
- Comparison of the quantitative fraction of hemo-/myoglobin signal determined by MSOT in patients with PD compared to healthy volunteers
- Comparison of the quantitative fraction of oxygenated/deoxygenated hemoglobin determined by MSOT in patients with PD compared to healthy volunteers

- Correlation of glycogen content determined with MSOT with disease duration/patient age
- Correlation of lipid content determined with MSOT with disease duration/patient age
- Correlation of collagen determined by MSOT with disease duration/patient age
- Correlation of haemoglobin/myoglobin content determined by MSOT with duration of disease/patient age
- Correlation of oxygenated/deoxygenated hemoglobin determined by MSOT with duration of disease / patient age

- Correlation of glycogen content determined with MSOT with R-Pact scale
- Correlation of lipid content determined with MSOT with R-PAct scale
- Correlation of collagen determined by MSOT with R-PAct scale
- Correlation of haemoglobin/myoglobin content determined by MSOT with R-PAct scale
- Correlation of oxygenated/deoxygenated hemoglobin determined by MSOT with R-PAct scale

- Correlation of glycogen content determined with MSOT with age-related functional muscle tests (Hammersmith Infant Neurological Examination (HINE)/The Children's

Content is confidential.

Hospital of Philadelphia Infant Test of Neuromuscular Disorders (CHOP Intend)/expanded Hammersmith functional motor scale (HFMSE)/ Revised Upper Limb Module (RULM)/6-Minute-Walk Test (6-MWT)/Time-to-go-up-and-go-test/MRC Muscle Strength Grades)

- Correlation of lipid determined with MSOT with age-dependent functional muscle tests (RULM /6-MWT/Time-to-get-up-and-go/MRC)
- Correlation of collagen determined with MSOT with age-dependent functional muscle tests (RULM/6-MWT/Time-to-get-up-and-go/MRC)
- Correlation of hemo-/myoglobin content determined with MSOT with age-dependent functional muscle tests (RULM/6-MWT/Time-to-get-up-and-go/MRC)
- Correlation of oxygenated/deoxygenated hemoglobin determined with MSOT with age-related functional muscle tests (RULM /6-MWT/Time-to-get-up-and-go/MRC)

- Correlation of glycogen content determined with MSOT with B-mode ultrasound (Heckmatt scale/Echogenity/Gray Scale Level/UGAP)
- Correlation of lipid determined with MSOT with B-mode ultrasound (Heckmatt scale/Echogenity/Gray Scale Level/UGAPI)
- Correlation of collagen determined with B-mode ultrasound (Heckmatt scale/Echogenity/Gray Scale Level/UGAP)
- Correlation of hemo-/myoglobin content determined with MSOT with B-mode ultrasound (Heckmatt scale/Echogenity/Gray Scale Level/UGAP)
- Correlation of oxygenated/deoxygenated hemoglobin determined with B-mode ultrasound (Heckmatt scale/Echogenity/Gray Scale Level/UGAP)

- Correlation of glycogen content determined with MSOT with respiratory function tests (Spirometry)
- Correlation of lipid content determined with MSOT with respiratory function tests (Spirometry)
- Correlation of collagen determined by MSOT with respiratory function tests (Spirometry)
- Correlation of haemoglobin/myoglobin content determined by MSOT with respiratory function tests (Spirometry)
- Correlation of oxygenated/deoxygenated hemoglobin determined by MSOT with respiratory function tests (Spirometry)

- Measurement of signal differences in right / left comparison

Content is confidential.

- Correlation of glycogen content determined with MSOT with (functional) magnetic resonance imaging parameters
- Correlation of lipid determined with MSOT with (functional) magnetic resonance imaging parameters
- Correlation of collagen determined with (functional) magnetic resonance imaging parameters
- Correlation of hemo-/myoglobin content determined with (functional) magnetic resonance imaging parameters
- Correlation of oxygenated/deoxygenated hemoglobin determined with (functional) magnetic resonance imaging parameters

Hypotheses:

- The optoacoustic spectrum of muscles of patients with PD is different compared to healthy volunteers
- The optoacoustic spectrum of the liver of patients with PD is different compared to healthy volunteers
- The quantitative fraction of glycogen signal in muscles determined by MSOT differs in patients with PD compared to healthy volunteers
- The quantitative fraction of lipid signal in muscles determined by MSOT differs in patients with PD compared to healthy volunteers
- The quantitative fraction of collagen signal in muscles determined by MSOT differs in patients with PD compared to healthy volunteers
- The quantitative fraction of hemo-/myoglobin in muscles determined by MSOT differs in patients with PD compared to healthy volunteers
- The quantitative fraction of oxygenated/deoxygenated hemoglobin in muscles determined by MSOT differs in patients with PD compared to healthy volunteers
- There is a correlation between MSOT derived glycogen signal and clinical status of patients with PD
- There are no side differences in patients with PD and healthy volunteers

Study type

Since no data exists so far to support the hypothesis of this study, it is an explorative study / pilot study.

8. Endpoint parameters

All measurements with MSOT are performed over the paraspinal musculature, Musculus trapezius as well as proximal and distal limb muscles in a right-left comparison (leg proximal: Musculus quadriceps, distal: Musculus triceps surae; arm proximal: Musculus biceps, distal: Forearm flexors) in healthy subjects compared to patients with PD.
Additionally, we will perform measurements of the liver with MSOT.

Primary endpoint:

Optoacoustic Absorption Spectrum of Muscle and liver in PD.
This target is measured non-invasively by MSOT.

Secondary endpoints:

Quantitative glycogen signal (in arbitrary units)
Quantitative lipid signal (in arbitrary units)
Quantitative collagen signal (in arbitrary units)
Quantitative hemo/myoglobin signal (in arbitrary units)
Muscle oxygenation (in %)
These target values are measured non-invasively by MSOT.

B-mode ultrasound

- Heckmatt scale
- Echogenitiy
- Gray Scale Level
- Ultrasound-Guided Attentuation Parameter (UGAP)

R-PaAt scale

- Summed raw score

Respiratory function test

- FVC
- FEV1

Functional magnetic resonance imaging of lung.

- Ventilation Defects / Non-defected
- Perfusion Defects / Non-defected
- Combined Defects / Non-defected

Content is confidential.

Magnetic resonance imaging of biceps muscle.

Correlation of glycogen detected by MSOT with clinical scores for determining muscle strength consisting of:

- Revised Upper Limb Module (RULM)
- MRC Muscle Strength Grades

in case of ability to walk additionally:

- 6-minute walking test
- Time-to-get-up-and-go-test

These target values are clinically determined at presentation (electronic patient record, physician letters):

Age
Sex
Weight
Skin color
Ethnic Background
Disease duration
Current medication
Results of last biopsy (if available)
Results of last MRI (if available)

Study Parameters	PD	HV
MSOT	X	X
-'Glycogen'	X	X
-Lipid	X	X
-Collagen	X	X
-Hemoglobin oxygenated	X	X
-Hemoglobin deoxygenated	X	X
Ultrasound	X	X
-Heckmatt	X	X
-Echogenity	X	X
-Muscle texture	X	X
-Grey scale level	X	X
-UGAP	X	X
Magnetic resonance imaging	X	X
-Ventilation defects	X	X
-Perfusion defects	X	X
-Combined defects	X	X
-Biceps muscle imaging	x	
R-Pact scale (≥16y)	X	X
Respiratory function test	X	X
-FVC	X	X
-FEV1	X	X
Muscle tests	X	X
-RULM	X	X
-6-MWT	X	X
-Time-to-get-up-and-go	X	X
-MRC	X	X

Patient History		
Confirmed Disease	X<br>Type:<br>Crim:	X
Diagnostics:	X	
-Genetics	X	
-Dried Blood Spot	X	
-Urinary Glc 4	X	
-Lymphozytes	X	
Muscle biopsy	X	
Neurological tests:	X	
-EMG	X	
-MRI	X	
-Sleep Study	X	
Lab values	X	
-CK	X	
-Liver enzymes	X	

Content is confidential.

Sitting ability<br>Walking ability	X<br>X	
Ventilation	X	
Medication		

9. Study design

Monocentric/Multicentric

This is a multicentric study with matched collectives (age, gender)/

Study arms: intervention/control

Interventions are not planned. A comparison is made between patients with PD and healthy volunteers. The study procedure is identical for PD patients and healthy volunteers. Healthy volunteers are used as controls (no muscular pathology, no excessive intramuscular lipids/collagen, no excessive glykogen) versus PD patients (muscular pathology, intramuscular lipids/collagen, excessive glycogen).

Randomization

Randomization is not planned. The allocation to the groups is based on the known diagnosis (PD, other muscular pathology) and controls (subjects).

Blinding

Blinding for the examination is not possible due to the possible clear clinical appearance. Blinding takes place during the measurement and and evaluation of the data. Blinding of the patients/controls is not necessary.

Content is confidential.

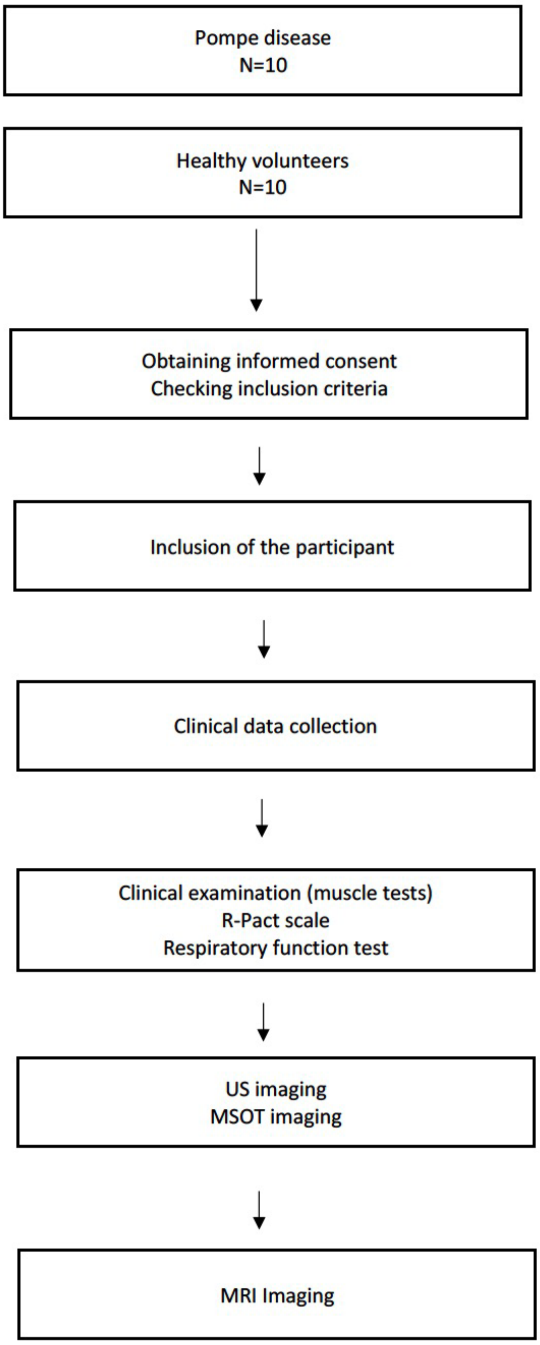
Pompe disease
N=10
Healthy volunteers
N=10
Obtaining informed consent
Checking inclusion criteria
Inclusion of the participant
Clinical data collection
Clinical examination (muscle tests)
R-Pact scale
Respiratory function test
US imaging
MSOT imaging
MRI Imaging

Study population

Inclusion and exclusion criteria

Inclusion criteria:
PD patients (IOPD and LOPD)

- Confirmed diagnosis of Pompe disease
- From 18 years of Age
- Independent from current therapy

Healthy controls

- From 18 years of Age, matched (age, gender) to PD collective

Exclusion criteria

PD patients

- Pregnancy
- Tattoo on skin to be examined

Healthy controls

- Anamnestic of other signs of myopathy or liver disease
- Pregnancy
- Tattoo on skin to be examined

Patient/control number

As this is a pilot study, an exact case number calculation is not possible. It is planned to study a total of 10 patients with PD and 10 healthy controls.

Recruitment routes and measures

Patients (and parents) are informed about the possibility of participating in the study in the context of an elective presentation at the Clinic for Pediatrics and Adolescent Medicine (Neuropediatrics) and the Clinic for Neurology at the University Hospital in Erlangen, Germany as well as the the Clinic for Pediatrics and Adolescent Medicine (Neuropediatrics) in Gießen, Germany. Additional recruitment options include the Clinic Rummelsberg, Schwarzenbruck, Germany, the German Society for muscular diseases (DGM), the treatNMD network and the international Pompe Association. If the patient is willing to participate, he/she will be fully

Content is confidential.

informed about the aims and methods (especially about the scientific/explorative character of the study), benefit and risk and revocability of the study participation. Patients in childhood and adolescence will also be informed and educated about the study and its procedure according to their age.

Healthy volunteers are recruited in the outpatient departments of the Clinic for Neurology at the University Hospital in Erlangen, Germany.

Acutely ill or unstable patients are not recruited. In the preliminary phase, volunteers are parallelised with the PD collective in terms of age and sex.

10. Study flow

Procedure for informing about and obtaining consent

Patients or test persons can only be included in the study after a written consent has been given. The written declaration of consent requires oral and written information of the patients/test persons as well as their parents or legal guardians about goals and methods (incl. scientific-explorative character of the study), benefit and risk as well as revocation of participation in the study. Children and adolescents are informed by means of age-appropriate, comprehensible patient information sheets. By giving their written consent, the patients/test persons and their parents/guardians declare that they agree to the collection and storage of study-relevant data and their verification by monitoring or authorities. The study participant must be clearly informed that the declaration of consent can be withdrawn at any time and without any disadvantage. Furthermore, all study participants/test persons and parents/guardians are informed that this study is a purely scientific study without any current diagnostic or therapeutic benefit.

The original of the declaration of consent will be kept in the study folder at the place of study. The patient/control and the parents/guardian receive a copy of the patient information and declaration of consent. The patient information and the consent form are attached to this protocol.

Measurements

After informing the patient / control and parent / guardian and obtaining consent, clinical scores are collected to assess muscle strength. R-Pact scale will be collected and respiratory function tests will be performed.

Subsequently, MSOT imaging is performed on 6 anatomical regions in all study participants: Paraspinal muscles, Musculus trapezius, upper/lower arm and upper/lower leg on predefined

Content is confidential.

muscle groups. (Leg proximal: Musculus quandriceps, distal: Musculus triceps surae; arm proximal: Musculus biceps, distal: Forearm flexors). The examination is analogous to sonography over the corresponding skin layers without further invasive procedures. The anatomical region can be localized by means of built-in B-image sonography; the corresponding optoacoustic signals can then be conducted. The duration per anatomical region is limited to 5 minutes; this corresponds to a maximum of 10 minutes for the parasinal muscles and Musculus trapezius, a maximum of 20 minutes for both upper extremities and 20 minutes for both lower extremities and 10 minutes for the examination of the liver. Patients can remain in a relaxed posture during the examination, without the need for breathing maneuvers or similar assistance.

At last, patients undergo MRI imaging of the lung and biceps muscle. Participants can be in a relaxed position during the examination. The time allotted for MRI is 30 minutes.

Recording of target parameters

- Determination of routine data (duration of disease, current medication)
- Clinical evaluation of muscle strength by 6-MWT, Time-to-get-up-and-go, MRC muscle test
- Assessment of R-Pact scale
- Performance of respiratory function test
- Non-invasive in-vivo measurement of glycogen, lipid, collagen, myo/hemoglobin content and oxygenation by MSOT

Content is confidential.

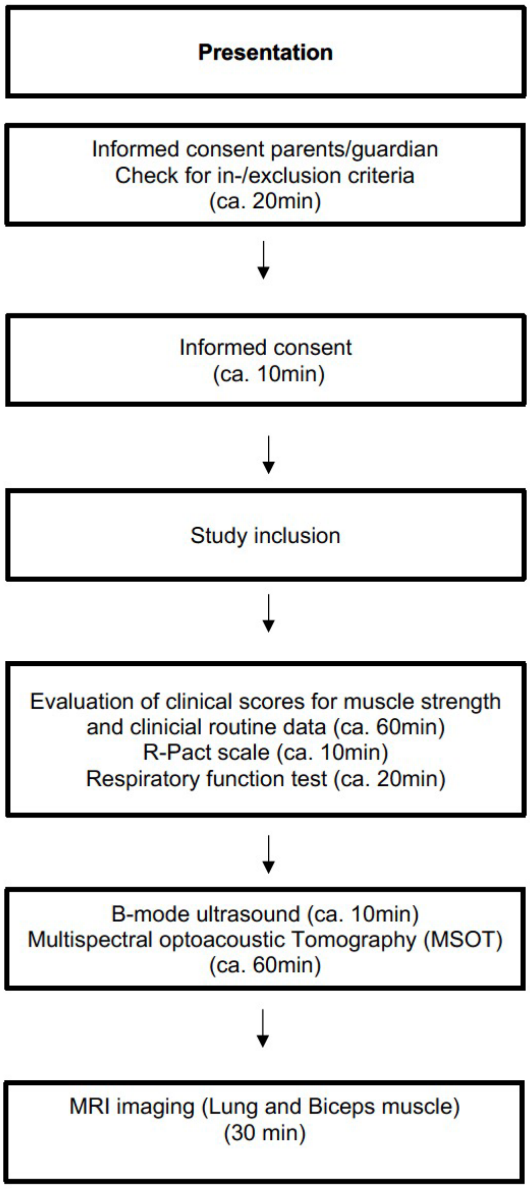

Time schedule and study duration for the individual patient/control

For the individual patient, the duration of the study participation is 220 minutes. Approximately 30 minutes are spent on education for study participants and parents/guardians, 60 minutes on clinical (routine) testing of muscle strength, 10 minutes on R-Pact scale, 20min on

Content is confidential.

respiratory function test, 10 minutes for B-mode ultrasound and 60 minutes for the MSOT examination. For MRI imaging 30 minutes are required.

Total duration of study

Depending on the number of patients, the expected total duration of the study up to the inclusion of the last patient is 8 months.

11. Benefit-risk-assessment

All study-related risks

Based on the classification criteria for medical devices (Directive 93/42/EEC, Annex IX), the iThera Medical optoacoustic system complies with Class IIa laser systems:

- Active diagnostic device
- Non-invasive
- Temporary use (<60 min)

The used system has CE-certification (TÜV Süd, 02.05.2021, type designation according to imprint: MSOT Acuity Echo). A conformity assessment procedure to expand the use of the system is not intended or planned by the manufacturer at this time. It is therefore a purely scientific pilot study. There is no dependency relationship with the manufacturer; all diagnostic and analytical procedures are available to the study directors on site. The cooperation with the company will be regulated in a separate contract drawn up by the legal department before the start of the study.

Compliance with energy limits

The laser safety and maximum permitted radiation dose for irradiation with laser pulses is regulated in the ANSI and IEC 60825 laser standards. The MSOT system complies with these standards and remains below the MPE (maximum permissible exposure) limits for skin irradiation and is therefore considered safe.

Temperature increases in tissue

Optoacoustic imaging does not result in a significant increase in tissue temperature. The absorption of a laser pulse in tissue results in a local transient temperature increase of a few millikelvin. Depending on the duration of the examination and the patient's skin type, there is typically a temperature increase in the range of less than one degree Kelvin.

Histological changes in tissue

Histological changes in the target tissue and surrounding structures are neither expected nor have they been observed in previous preclinical and clinical studies.

Slight, reversible redness or warming is only to be expected in sensitive skin.

Such impairment of the patient can be noticed by the test person or physician at any time; the examination can then be interrupted or discontinued. In any case, no irreversible damage is to be expected.

In principle, the near-infrared light used in the MSOT can lead to retinal damage if the eye is irradiated. To prevent this, the participants and examiners will wear appropriate laser safety glasses during the examination.

Since the data obtained will not be used to interpret findings, there is no risk of possible misdiagnosis or incorrect display of data in this exploratory pilot study.

There are no other risks within the scope of this study, nor have they been described based on our own preliminary data.

Magnetic resonance imaging

Unlike computed tomography, MRI does not use ionizing radiation, so no permanent side effects are to be expected.

More than 1 million MRI examinations at higher field strengths (1.5/3T) are performed annually in Germany. Provided that the general contraindications for MRI examinations are observed, no serious side effects occur. MRI is therefore one of the safest examination procedures.

The risks associated with an MRI examination emanate from the three main components of the MRI system.

Static magnetic field

The static magnetic field exerts forces and torques on ferromagnetic objects that can be so strong that the (mostly ferromagnetic) objects fly uncontrollably toward the magnet and can hit patients and staff (missile effect). The magnetic forces are proportional to the field strength B and the field change with location (dB/dz). These risks are lower with the low-field MRI system. Risks are further minimized by providing safety training to operators and excluding patients with ferromagnetic implants from the study.

The gradient system

Content is confidential.

Gradient switching can cause the appearance of magnetic phosphenes and nerve and muscle cell stimulation. Rapidly switched gradients produce high magnetic field changes per time (dB/dt) that induce voltages in the body. If a current flows through the tissue via nerve endings, for example, this can result in so-called peripheral nerve stimulation. However, the manufacturer of the gradient system guarantees compliance with the limits for gradient switching times and amplitudes recommended in the IEC 60601-2-33 guidelines. Thus, nerve stimulation effects need not be considered further in the risk assessment of this study.

Another safety-relevant effect of gradient fields is noise caused by gradient switching due to current- and field-strength-dependent Lorentz forces in the gradient tube. These unpleasant loud knocking noises occur especially during fast imaging processes where high currents flow through the gradients. Noise levels can rise up to 115dB for 1.5T tomographs (background noise: approx. 78dB). Due to the lower magnetic forces at 0.55T, we expect lower noise levels. In addition, patients always wear hearing protection during the examination, so that the noise exposure remains well below the legal limit of 99 dB.

The high-frequency system

During the MR measurement, radiofrequency (RF) fields are sent into the human body, which are partially absorbed by the tissue and can lead to an increase in body temperature. The thermoregulatory response of human tissue to RF pulses has now been studied for 50 years. For example, using conservation of energy, it has been calculated that the body temperature of lightly clothed patients with undisturbed thermoregulation at room temperature increases by up to 0.6 °C with RF exposure of 4 W/kg (63 MHz, 1.5 Tesla). The assumed specific absorption rate (SAR) of 4 W/kg body tissue corresponds to the so-called "controlled mode first level" (IEC safety guideline), which is also used as an upper limit in routine clinical imaging. The magnitude of the actual temperature rise is generally smaller because skin cooling was not considered in the calculations (worst case scenario).

The body's energy production at rest is about 1.2 W/kg - equivalent to the energy conserved when wearing a thin sweater. Most healthy people are capable of compensating for 15 times this resting energy, and only a minimal increase in core body temperature occurs. Studies at 1.5 T have shown that RF absorption in humans leads only to the expected cardiac adaptation and does not cause adverse health effects. Theoretically, a 63 kg person is even capable of emitting 1296 W to the environment through the skin by cardiac adaptation (i.e., maximum increase in blood flow) - this would correspond to a SAR of 20.6 W/kg.

The same limits are observed with the 0.55 Tesla MRI system. At 0.55 Tesla, the wavelength of the radio waves used is significantly longer, so that the spatial distribution of the energy emission is more homogeneous and thus the risks tend to be lower.

12. Biometrics

Exploratory study: Explanation of the statistical methodology, justification of the chosen case number

Case number calculation

As this is a pilot study and no information is available on the expected differences between the different groups, no case number calculation was carried out. The number of cases given represents an estimate or is within reasonable limits for a pilot study.

Statistical Methodology

Continuous variables are given as mean values with standard deviation, categorical variables as numbers with percentages where appropriate. The MSOT parameters are compared using a two-sided, unpaired t-test with equal deviations. If the standard deviation is not equal, Welch correction is applied where appropriate. ROC (Receiver Operator Characteristics) analyses between healthy and sick persons are also planned. Genetics serves as the gold standard. Correlations are indicated by the Pearson coefficient. All statistical tests will be double-sided and a p-value of <0.05 is considered statistically significant. All analyses are performed with GraphPad Prism (version 7.00 or later, GraphPad Software, La Jolla, CA, USA), RStudio (version 1.1.456 or later, RStudio Inc., Boston, MA, USA) or IBM SPSS Statistics (version 24 or later, IBM Corp., Armonk, NY, USA).

13. Data management and protection

All raw data, such as patient records, represent source documents. Their availability is ensured for routine monitoring. The participation of the individual patients or test persons in the study is documented; the study director maintains a separate list to identify the all screened and participating patients. This list contained the names and date of birth as well as the examination date and pseudonymization abbreviations of the patients and test persons. The study director is responsible for the quality of data collection and storage. The data storage (total data) is carried out on computers or specially designed network drives of the University Hospital Erlangen. The raw imaging data (no patient-related data) is stored on specially designated servers of the company iThera Medical GmbH.

Pseudonymisation

Prior to a scientific analysis of the materials and data of this study, all information will be pseudonymised in accordance with the guidelines of the Federal Data Protection Act.

Data transfer

Content is confidential.

Data sharing in this study is intended solely for the MSOT raw data. The company iThera Medical GmbH will work with this data to ensure adequate recording quality and to develop algorithms for evaluation. The data will only be transferred pseudonymized on encrypted physical drives. The data will not be used for a later approval of the prototype used. The cooperation will be explicitly regulated by the legal department before the study starts (a supplementary agreement to the service contract can be found in the appendix).

The study results can be published anonymously, but it will not be possible to draw conclusions about the identity of the participating persons. The data will be kept for 10 years and destroyed afterwards.

Revocation, data deletion

If the declaration of consent is revoked, data collected up to this point can be taken into account. The patient has the right to demand their destruction, provided that legal provisions do not conflict with the destruction.

14. Biomaterial handling

No biomaterials are obtained.

15. Individual paticipant insurance

The participants of the study are insured through the group contract of the CCS Erlangen.

16. Signatures

Dr. med. Ferdinand Knieling
Study director

Prof. Dr. med. Regina Trollmann
Study director

Prof. Dr. med. Joachim Wölfle
Clinic director

Content is confidential.

4. Study Protocol (External Cohort)

Study protocol

Title of the study:
Multispectral Optoacoustic Tomography (MSOT) for the translational molecular imaging in neuromuscular diseases

Version 2, 03.07.2023

Responsibilities and contact:

Head of clinical/scientific testing
PD Dr. Jana Zschüntzsch Oberärztin in der Klinik für Neurologie
Universitätsmedizin Göttingen Robert-Koch-Str. 40
37075 Göttingen

Vertreter:
Dr. med. Stefanie Meyer
Assistenzärztin in der Klinik für Neurologie
Universitätsmedizin Göttingen Robert-Koch-Str. 40
37075 Göttingen

Department Head:
Prof. Dr. med. M. Bahr
Direktor der Klinik für Neurologie
Universitätsmedizin Göttingen Robert-Koch-Str. 40
37075 Göttingen

Prof. Dr. G. Hasenfuß
Direktor der Klinik für Kardiologie und Pneumologie
Universitätsmedizin Göttingen
Robert-Koch-Str. 40
37075 Göttingen

Prüfzentren:
Klinik für Neurologie
Universitätsmedizin Göttingen
Robert-Koch-Str. 40
37075 Göttingen

Klinik für Kardiologie und Pneumologie
Universitätsmedizin Göttingen
Robert-Koch-Str. 40
37075 Göttingen

Translated from the original protocol

Examiners:

PD Dr. med. Jan Koch
Oberarzt der Klinik für Neurologie
Universitätsmedizin Göttingen
Robert-Koch-Str. 40
37075 Göttingen

Dr. med. Ulrike Olgemöller
Oberärztin der Klinik für Kardiologie und Pneumologie
Universitätsmedizin Göttingen
Robert-Koch-Str. 40
37075 Göttingen

Dr. med. Stefanie Glaubitz
Assistenzärztin der Klinik für Neurologie
Universitätsmedizin Göttingen
Robert-Koch-Str. 40
37075 Göttingen

Dr. med. Karsten Kummer
Facharzt der Klinik für Neurologie
Universitätsmedizin Göttingen
Robert-Koch-Str. 40
37075 Göttingen

Dr. med. Rachel Zeng
Assistenzärztin der Klinik für Neurologie
Universitätsmedizin Göttingen
Robert-Koch-Str. 40
37075 Göttingen

Dr. med. Lina Hassoun
Fachärztin der Klinik für Neurologie
Universitätsmedizin Göttingen
Robert-Koch-Str. 40
37075 Göttingen

The study leader PD Dr. med. J. Zschuntzsch selects sufficiently qualified doctors to carry out the study and documents their consent and instruction in tasks within the scope of the study in an appropriate protocol.

Translated from the original protocol

Signatures of department heads and examiners
(not included, please see original study protocol)

Translated from the original protocol

Study status
First patient in (FPI): 21.04.2023
Current status: ongoing

Translated from the original protocol

Abstract of the study

Title of the study: Multispectral Optoacoustic Tomography (MSOT) for the translational molecular imaging in neuromuscular diseases

Study Lead:
PD Dr. Jana Zschüntzsch

Project description:
The spectrum of neuromuscular diseases includes a large number of different disease entities of the skeletal muscles and the peripheral nervous system, for example inflammatory and hereditary myopathies, spinal muscular atrophy (SMA) or Pompe disease, a glycogen storage disease with muscular involvement. Although all of these diseases are generally characterised by muscle weakness with associated loss of mobility, the clinical symptoms presented can be diverse. Due to this variability in phenotypes and disease progression, diagnosis is often difficult and sometimes delayed for years. Depending on the underlying cause of the disease, different therapeutic options can be offered, meaning that a reliable diagnosis is fundamental to implementing the best possible patient care. In addition to diagnostic pitfalls, therapy monitoring for various neuromuscular diseases also poses a challenge due to a lack of non-invasive and easily accessible biomarkers. While muscle biopsy remains the gold standard for diagnosis, regular bioptic examination of the muscle structure for follow-up and therapy monitoring cannot be expected of patients due to its invasiveness and associated risks. The frequently used EMG is also an invasive method with limited informative value as a follow-up examination. MRI examinations of the musculature are expensive and time-consuming and are therefore only suitable to a limited extent for regular follow-up examinations, particularly in clinical practice. The establishment of innovative, non-invasive and reliable biomarkers for diagnostics and follow-up monitoring is therefore an important aspect in improving the care of patients with neuromuscular diseases.

Multispectral optoacoustic tomography (MSOT) allows the detection of specific endogenous chromophores such as collagen, myoglobin or haemoglobin by using a non-invasive approach comparable to conventional ultrasound. Instead of sound waves, however, MSOT illuminates the tissue with light from transient energy close to the infrared spectrum, which is absorbed and results in a thermo-elastic expansion of certain tissue molecules. This expansion leads to the generation of sound waves which are detected by the same device. The use of multispectral illumination and subsequent unmixing allows the precise localisation and quantification of muscle-specific subcellular structures. MSOT has already shown the potential to image the muscular structure and clinical expression of muscle diseases in children with Duchenne muscular dystrophy and SMA and to differentiate these patients from healthy subjects.

The aim of this study is to better differentiate the muscle structure of patients with neuromuscular diseases and to identify disease-specific parameters in the muscle. Differences to an age- and gender-matched cohort of non-myopathic subjects should be made clear. Already established target structures of MSOT imaging, such as collagen, myoglobin, haemoglobin and possibly glycogen, are to be quantified for various disease entities of the neuromuscular spectrum. In particular for patients with Pompe disease, glycogen is to be established as a novel Pompe-specific imaging marker using MSOT. The establishment of a disease-specific signature should help to facilitate the diagnosis and identification of patients with neuromuscular diseases in the future and support the differentiation between different disease modalities in this spectrum. Such a signature could represent a non-invasive biomarker in the future. Only through increased diagnostic certainty can patients be offered adequate treatment.

Translated from the original protocol

Content

1. Scientific background 7
2. Aim of the study 9
3. Endpoint parameters 12
4. Study design und flow 15
5. Recruitment of control subjects 17
6. Recrutiment of patients 18
7. Benefit-risk anaylsis 19
8. Biometrics 20
9. Ethical considerations, archiving and data protection 20
10. Literature 22
Appendix 26

Translated from the original protocol

Study:

Study title: ***Multispectral Optoacoustic Tomography (MSOT)* for the translational molecular imaging in neuromuscular diseases**

1. Scientific background

Neuromuscular diseases include a diverse spectrum of diseases of the skeletal muscle and peripheral nervous system. Both the underlying causes and the clinical symptoms and progression can be very heterogeneous. What these diseases have in common, however, is the manifestation of muscle weakness and paresis, which can lead to a loss of mobility and serious restrictions in the daily lives of those affected. With regard to the causes of the disease, a distinction can first be made between hereditary and acquired diseases.

One example of a genetic disease of the neuromuscular spectrum is spinal muscular atrophy (SMA).SMA is one of the most common autosomal recessive inherited diseases of childhood in Germany with a frequency of 1:7000 among newborns (Vill et al. 2019; Wirth et al. 2020).In 96% of SMA patients, there is a homozygous deletion in the SMN1 gene (survival motor neuron gene 1) on chromosome 5q, which leads to insufficient expression of the SMN (survival motor neuron) protein (Lefebvre et al. 1995).Deficiency of the SNM protein leads to degeneration of the motor neurones of the spinal cord and brain stem with consecutive muscle atrophy and weakness (Lefebvre et al. 1995; Dubowitz 2009). The closely related SMN2 gene can partially compensate for the loss of SMN1 by producing the SMN protein, so that the clinical phenotype can be milder in individuals with a high SMN2 copy number (Mailman et al. 2002; Butchbach 2016). According to a classification of the international SMA consortium, SMA was categorised into subtypes one to four based on the onset of the disease and milestones reached or clinical phenotype. Secondly, SMA 0 with intrauterine onset or before the 7th day of life and lethality in the first 6 months of life is also listed separately (Pearn et al. 1973; MUNSAT 1991).However, as this classification does not reflect the continuum in the overall spectrum of the disease and modified phenotypes due to access to new treatment options, a revised classification was introduced in 2020, which differentiates between "non-sitter", "sitter" and "walker" clinical phenotypes (Finkel et al. 2014; Finkel et al. 2018).The approval of the first intrathecal treatment to restore the SMN protein was an important milestone in SMA treatment (Rigo et al. 2012; Finkel et al. 2017).There are now other therapies that attempt to intervene in the course of the disease by modulating SMN2 gene splicing (Sivaramakrishnan et al. 2017) or through SMN1-AAV9 gene replacement therapy (Foust et al. 2010; Valori et al. 2010; Mendell et al. 2017).Despite the high level of attention paid to the development of therapeutic options, there are still gaps in SMA treatment in terms of monitoring disease progression and establishing suitable biomarkers (Bonati et al. 2017; Kariyawasam et al. 2019; Faravelli et al. 2020). Modern and effective therapeutic approaches in particular will mean that altered disease progression in patients with SMA will play an increasingly important role in both paediatric and adult neurology. The establishment of non-invasive, reliable and standardised biomarkers is particularly important for patients who will experience previously unknown disease progressions after receiving innovative therapies Significance for disease and therapy monitoring. The use of MSOT in children with SMA has already been investigated in an exploratory pilot study involving 10 children.The muscle structure of the study participants with SMA showed "moth-eaten" optoacoustic signal patterns compared to a uniform signal distribution in healthy subjects.The greatest differences between patients and healthy individuals were identified in the haemoglobin signal, the intensity of which correlated with the clinical phenotype in SMA patients (Regensburger et al. 2022). To our knowledge, there have been no studies on adult patients with SMA to date.

Another hereditary disease from the spectrum of neuromuscular diseases is Pompe disease (PD), a rare autosomal recessive disease caused by a deficiency of lysosomal acid alpha-glucosidase (GAA). This enzyme deficiency leads to generalised deposition of glycogen, particularly in the heart, liver, CNS and skeletal muscle (Pompe 1932, Hers 1963).It is a progressive disease with

Translated from the original protocol

variable onset, severity and organ involvement, which has led to the clinical differentiation into different disease types. In general, it is divided into an infantile (IOPD) and a late-onset (LOPD) form, with IOPD also being subdivided into classic IOPD with concomitant cardiomyopathy and non-classic IOPD without cardiac involvement (Hers 1963; Slonim et al. 2000; Kishnani and Howell 2004; Kishnani et al. 2006b). What all phenotypes have in common, however, is the progressive course of the disease in the absence of therapy. In adult neurology, LOPD plays a predominant role, which describes an onset after the age of 12 months and is characterised by slowly progressive muscle weakness with limb girdle distribution type without significant accompanying cardiomyopathy (Cupler et al. 2012; Preisler et al. 2013). The diagnosis is usually made on the basis of the clinical presentation and subsequent confirmation by detection of GAA deficiency using a dry blood card or alternatively a GAA activity measurement in lymphocytes, muscle or fibroblasts as well as genetic examinations (van den Hout et al. 2003; Case et al. 2012). All of the aforementioned diagnostic methods are invasive procedures that are associated with risks for the patient. Due to the slowly progressive course, the limb girdle distribution pattern and the onset of symptoms in adolescence or early adulthood, the differential diagnosis of other hereditary myopathies such as muscular dystrophies plays a relevant role in the diagnosis. Early identification of the disease is highly relevant, particularly in PD, as an early start to treatment is likely to lead to an improved treatment outcome. Enzyme replacement therapy with GAA (recombinant human GAA, rhGAA) has been approved as a therapy, which prolongs the survival of patients and leads to an improvement in motor and cardiac function (Kishnani et al. 2007). Regardless of the time of onset of the disease, prospective follow-up monitoring is recommended for all patients with PD (Kishnani et al. 2006a; Bembi et al. 2008). However, there is currently a lack of cross-centre standards for this.Various examination methods including laboratory parameters, cardiac and respiratory function tests and muscle-specific tests such as EMG or clinical examinations are currently used for monitoring the course of the disease.The establishment of non-invasive methods both in diagnostics and for monitoring the course of the disease and the success of therapy are of fundamental importance here.Correlations between the success of therapy and structural muscle changes have already been shown using MRI, for example.A correlation between fatty degeneration in muscle MRI and muscle function in adult-onset PD was demonstrated in a study (Figueroa-Bonaparte et al. 2016). A correlation between the pre-existing degree of muscle fatty degeneration and the subsequent effectiveness of long-term enzyme replacement therapy was also demonstrated (Gruhn et al. 2015).

In addition to the hereditary diseases SMA and PD, hereditary myopathies can also occur with other genetic changes. The genetic variants and clinical courses are very heterogeneous.During the course of the disease, changes in muscle structure and associated muscle weakness, atrophy and paresis occur, often due to genetic deficiencies in muscle structural proteins (Oldfors 2007; Carter et al. 2018; Danielsson and Häggqvist 2021).Due to the heterogeneity of these diseases, the diagnosis can often be more difficult and is sometimes only made after a significant delay before the first symptoms appear. In particular, differentiation from other myopathies, such as Pompe disease, can be problematic with similar disease characteristics and lead to delays in treatment. Using the example of Duchenne muscular dystrophy (DMD), a study has already shown that MSOT can be used to reliably differentiate between healthy test subjects and children with DMD (Regensburger et al. 2019). A correlation between the collagen content of muscle tissue measured using MSOT and the clinical phenotype of DMD patients was observed. Due to the limited treatment options for this hereditary muscular dystrophy, in which dystrophin deficiency leads to remodelling and fatty degeneration of the muscles, and the increased mortality due to accompanying cardiomyopathy and respiratory complications, DMD plays a subordinate role in adult neurology (Ryder et al. 2017). However, other muscular dystrophies, such as Becker muscular dystrophy or limb girdle dystrophies, represent relevant disease patterns. The translation of the identification of structural muscle changes using MSOT in DMD into other muscular dystrophies represents an innovative possibility for improving the diagnosis of these diseases. There are currently only limited therapeutic options for hereditary myopathies (Claeys 2020; Mendell et al. 2021). However, due to the rapidly advancing development of genetic therapies, there is hope that specific therapies will also be available for these diseases in the future, similar

Translated from the original protocol

to those for SMA. Particularly with regard to this, increased diagnostic certainty and the establishment of non-invasive, easily accessible biomarkers are of great importance.

In addition to the hereditary diseases discussed above, idiopathic inflammatory myopathies (IIM, synonym myositis) play an important role in the spectrum of neuromuscular diseases.IIMs include polymyositis (PM), dermatomyositis (DM), necrotising myopathy (NM), inclusion body myositis (IBM) and overlap syndromes (OM), including anitsynthetase syndrome (ASS) (Schmidt 2018).Depending on the specific subtype of IIM, there are heterogeneous disease courses and distribution patterns.However, all entities are characterised by muscle weakness and the development of paresis.Some IIMs are accompanied by muscle pain and possibly extramuscular manifestations such as characteristic skin involvement in DM (Cassius et al. 2019). In addition to the medical history and clinical presentation, laboratory parameters (CK, autoantibodies) and electrophysiological diagnostics (EMG) play an important role in the diagnosis. The gold standard in diagnostics is still the muscle biopsy, in which characteristic signs of muscle damage can be found, depending on the respective subtype of IIM (Schmidt 2018). In the majority of IIM, immunomodulatory therapy can lead to an improvement in symptoms; only in IBM is it generally not possible to achieve sufficient treatment success, even with immunosuppression (Keller et al. 2017).Attempts are made to monitor treatment based on the patient's subjective perception, laboratory parameters, electrophysiology and imaging diagnostics.However, in addition to the invasiveness of some of these biomarkers, objectifying treatment success in myositis treatment is often a challenge.The establishment of reliable, non-invasive biomarkers, particularly for therapy monitoring, but also for diagnosis, is therefore also necessary with regard to inflammatory myopathies.In particular, the differentiation between active muscle inflammation and already completed muscle remodelling can represent a relevant parameter here, which could possibly be mapped using the MSOT.There are already a large number of studies on the use of optoacoustic imaging techniques in the imaging of inflammation, particularly in the cardiovascular, dermatological or gastrointestinal areas (Regensburger et al. 2021), but the use of human skeletal muscle for the detection of inflammation has not yet been evaluated to any great extent.

For MSOT, an ultrasound probe is placed on the patient's skin in a similar way to conventional ultrasound. Instead of sound waves, MSOT illuminates the tissue with light from transient energy. Light pulses, typically with wavelengths close to the infrared spectrum, are absorbed by the tissue and lead to a thermo-elastic expansion of certain molecules. This expansion leads to the generation of ultrasound waves, which can be detected by the same device. Studies have already shown that MSOT-based evaluation of haemoglobin levels in the intestinal wall has the potential to assess disease activity in Crohn's disease (Waldner et al. 2016; Knieling et al. 2017). By using a newly configured device (Acuity Echo, iThera Medical GmbH, Munich, custom build platform), an extended spectrum of laser light can be used, which, in addition to the detection of haemoglobin, also allows the detection of other biomarkers such as collagen or lipids. In a study at the University of Erlangen, MSOT was used to visualise the molecular composition of muscles in Duchenne muscular dystrophy. The application in children with SMA was also investigated in a further study. The results of both studies suggest the importance of non-invasively measured collagen and haemoglobin in muscle as a possible new biomarker for the disease activity of these diseases (Regensburger et al. 2019; Regensburger et al. 2022).

In this study, the application of MSOT in adult patients with neuromuscular diseases will be investigated. Building on the promising data from the investigations of children with SMA and DMD, the aim is to evaluate whether structural differences to non-myopathic test subjects can be detected in the muscle of adult patients and whether parameters identifiable using MSOT show a correlation to clinical disease activity. The aim is to determine non-invasive, reliable and reproducible biomarkers for monitoring disease progression and treatment success.

2. Aim of the study

Translated from the original protocol

The aim of this study is to translate the promising results of the use of MSOT in the examination of skeletal muscle in juvenile patients with Duchenne muscular dystrophy and SMA to other diseases of the neuromuscular spectrum and to examine possible differences to healthy control subjects. By using this non-invasive, low-risk method, the aim is to examine whether different specific structural profiles of the skeletal muscle can be mapped for various nerve and muscle diseases in adulthood (SMA, myositis, hereditary myopathies, Pompe disease) and to what extent these allow a distinction to be made between disease spectra. Possible correlations between the occurrence of these proteins and the severity of the disease will also be identified. By evaluating the imaging capability of various markers (collagen, haemoglobin, myoglobin, glycogen), possible non-invasive biomarkers are to be detected that may in future allow conclusions to be drawn about disease activity in various diseases.

<u>Secondary study objectives:</u>

- Comparison of quantitative glycogen signalling in MSOT between SMA, myositis, hereditary myopathies, Pompe disease and non-myopathic control subjects
- Comparison of the quantitative lipid signal proportion in the MSOT between SMA, myositis, hereditary myopathies, Pompe disease and non-myopathic control subjects
- Comparison of the quantitative collagen signal proportion in the MSOT between SMA, myositis, hereditary myopathies, Pompe disease and non-myopathic control subjects
- Comparison of quantitative haemo/myoglobin signal content in MSOT between SMA, myositis, hereditary myopathies, Pompe disease and non-myopathic control subjects
- Comparison of the quantitative oxygenated/deoxygenated haemoglobin signal content in the MSOT between SMA, myositis, hereditary myopathies, Pompe disease and non-myopathic control subjects- Correlation of the glycogen content determined by MSOT with disease duration / patient age- Correlation of the lipid content determined by MSOT with disease duration / patient age
- Correlation of the collagen content determined by MSOT with disease duration / patient age
- Correlation of the haemo/myoglobin content determined by MSOT with disease duration / patient age
- Correlation of the determined oxygenated / deoxygenated haemo/myoglobin content by MSOT with disease duration / patient age
- Correlation of the glycogen content determined by MSOT with disease-specific disease activity scores (see below)- Correlation of the collagen content determined by MSOT with disease-specific disease activity scores (see below)- Correlation of the lipid content determined by MSOT with disease-specific disease activity scores (see below)
- Correlation of the haemo/myoglobin content determined by MSOT with disease-specific disease activity scores (see below)
- Correlation of the determined oxygenated/deoxygenated haemo/myoglobin content by MSOT with disease-specific disease activity scores (see below)
- Correlation of the glycogen content determined by MSOT with examination results relating to muscle function (6 minute walk test, MRC Muscle Strength Grades, Time to get up and go test)
- Correlation of the collagen content determined by MSOT with examination results regarding muscle function (6 Minute walk test, MRC Muscle Strength Grades, Time to get up and go test)
- Correlation of the lipid content determined by MSOT with examination results regarding muscle function (6 Minute walk test, MRC Muscle Strength Grades, Time to get up and go test)
- Correlation of the hemo/myoglobin content determined by MSOT with test results regarding muscle function (6 minute walk test, MRC muscle strength grades, time to get up and go test)

Translated from the original protocol

- Correlation of the oxygenated / deoxygenated hemo/myoglobin content determined by MSOT with test results regarding muscle function (6 minute walk test, MRC Muscle Strength Grades, Time to get up and go test/UGAP)
- Correlation of the glycogen content determined by MSOT with B-mode ultrasound (Heckmatt scale / echogenicity / gray scale analysis/UGAP)
- Correlation of the lipid content determined by MSOT with B-mode ultrasound (Heckmatt scale / echogenicity / gray scale analysis/UGAP)
- Correlation of the collagen content determined by MSOT with B-mode ultrasound (Heckmatt scale / echogenicity / gray scale analysis/UGAP)
- Correlation of the determined hemo/myoglobin content by MSOT with B-mode ultrasound (Heckmatt scale / echogenicity / gray scale analysis/UGAP)
- Correlation of the determined oxygenated / deoxygenated hemo / myoglobin content by MSOT with B-mode ultrasound (Heckmatt scale / echogenicity / gray scale analysis / UGAP)
- Correlation of the glycogen content determined by MSOT with lung function tests (spirometry)
- Correlation of the lipid content determined by MSOT with lung function tests (spirometry)
- Correlation of the collagen content determined by MSOT with lung function tests (spirometry)
- Correlation of the hemo/myoglobin content determined by MSOT with lung function tests (spirometry)
- Correlation of the oxygenated/deoxygenated hemo/myoglobin content determined by MSOT with lung function tests (spirometry)
- Examination of signal differences in the right/left side comparison

<u>Hypotheses:</u>
- There are differences in the optoacoustic spectrum of muscles in patients with different neuromuscular diseases between the different diseases and compared to non-myopathic control subjects
- The optoacoustic spectrum of the liver of patients with Pompe disease differs from that of patients with other NMDs and healthy volunteers
- The quantitative muscle glycogen content, as determined by MSOT, in patients with Pompe disease differs from that in patients with other NMDs
- The quantitative collagen content of the muscle, determined using MSOT, differs in patients with various neuromuscular diseases
- The quantitative lipid content of muscle, determined using MSOT, differs in patients with various neuromuscular diseases
- The quantitative hemo/myoglobin content of the muscle, as determined by MSOT, differs in patients with various neuromuscular diseases
- The quantitative oxygenated/deoxygenated hemo/myoglobin content of the muscle, as determined by MSOT, differs in patients with various neuromuscular diseases
- There is a correlation between MSOT-determined glycogen signaling and disease activity in patients with Pompe disease
- There are correlations between the lipid content and collagen content of the muscle with the disease activity in patients with NMD
- No side differences can be determined between examinations of the right and left sides of the body

Translated from the original protocol

3. Endpoint parameters

All measurements using MSOT are carried out on the paraspinal muscles as well as on the trapezius, rectus abdominus and the proximal and distal limb muscles in a right vs. left comparison (leg prox: M. quadriceps ischiocrural muscles, dist: M. triceps surae, M. tibialis anterior; arm prox.: biceps muscle, dist.: forearm flexors). A comparison is made between patients with various neuromuscular diseases and healthy control subjects. MSOT examinations of the liver are also carried out.

Primary Endpoint: Optoacoustic absorption spectrum of skeletal muscle and liver in various neuromuscular diseases. *This target variable is measured non-invasively using MSOT*

Secondary endpoints:
- Quantitative glycogen signal (in relative units / arbitrary units)
- Quantitative lipid signal (in relative units / arbitrary units)
- Quantitative collagen signal (in relative units / arbitrary units)
- Quantitative hemo-/myoglobin signal (in relative units/arbitrary units)
- Muscle oxygenation (in%)

These target variables are measured non-invasively using MSOT.

B-mode ultrasound:
- Heckmatt scale
- Echogenicity
- Gray scale analysis
- Ultrasound Guided Attenuation Parameters (UGAP)

These target variables are measured non-invasively using ultrasound.

Clinical course parameters:
- Muscle strength test (MRC total score*)
- 6 minute walk test (time in min)
- Time to get up and go test (time)
- Disease-specific scales:
- Pompe disease: R-PAct scale (sum score)
- Myositis: HAQ, PGAA, IBM-FRS, SSQ
- SMA: EQSD-5L, ECAS, ALSFRS, NMSQuest
- Hereditary myopathies: ACTIVLIM, EQ-5D**
- Pulmonary function testing
- Respiratory muscle function test (PImax p 0.1)
- Body plethysmography including total lung capacity, FVC, FEV1
- Sonography of the diaphragm
- Blood collection
- Hemoglobin (Hb)
- Creatine kinase (CK)
- HbA1c

Remarks:
* Application of the MRC sum score according to the usual clinical examination of the UMG Department of Neurology, including head flexion, arm abduction, elbow flexion, wrist extension, hip flexion, knee extension, foot elevation; Maximum total score: 65 points.
** There are no generally validated patient-reported outcome scales in Germany for hereditary myopathies; scales are therefore used for neuromuscular diseases in relation to the performance of everyday activities (ACTIVLIM, (Vandervelde et al. 2007)) as well as generally validated scales regarding health-related quality of life (EQ-5D, (EuroQol Group 1990)) was used.

Translated from the original protocol

Table 1 Overview of the clinical course parameters collected

SMA	Myositis	Hereditary Myopathy	Pompe Disease	Non-myopathic control
MRC	MRC	MRC	MRC	MRC
6MWT	6MWT	6MWT	6MWT	6MWT
Timed up and go	Timed up and go	Timed up and go	Timed up and go	Timed up and go
EQSD-5L, ECAS, ALSFRS, NMSQuest	HAQ, PGAA, IBM-FRS, SSQ	ACTIVLIM, EQ-5D	R-PAct-Skala	n.a.

<u>The following target variables are collected when the patients are presented</u> (electronic patient files, doctor's letters):

- Age
- Gender
- Weight
- Skin color
- Ethnicity
- Duration of illness
- Current medication
- If applicable, result of the last muscle biopsy (if available)
- If applicable, result of the last MRI (if available)

Translated from the original protocol

Table 2 Overview of the recorded study parameters

Study parameters	SMA	Myositis	Hereditary Myopathien	Pompe disease	Control
**MSOT**	X	X	X	X	X
- Glycogen	x	x	x	x	x
- Lipid	x	x	x	x	x
- Collagen	x	x	x	x	x
- Haemoglibin oxygenated	x	x	x	x	x
- Haemoglibin deoxygenated	x	x	x	x	x
**Ultraschall**	X	X	X	X	X
- Heckmatt scale	x	x	x	x	x
- Echogenity	x	x	x	x	x
- Muscle structure	x	x	x	x	x
- Grey scale analysis	x	x	x	x	x
- UGAP	x	x	x	x	x
**Klinische Verlaufsskala**	X	X	X	X	
**Lung function**	X	X	X	X	X
- Bodyplethysmography (incl. FVC, FEV1, PImax)	x	x	x	x	x
- Sonography oft he diaphragm	x	x	x	x	x
**Blood analyses**	X	X	X	X	X
- Haemoglobin (Hb)	x	x	x	x	x
- CK	x	x	x	x	x
- HbA1c	x	x	x	x	x
**Muscle investigations**	X	X	X	X	X
- MRC	x	x	x	x	x
- 6MWT	x	x	x	x	x
- Get up and go	x	x	x	x	x

Tabelle 3 Übersicht der festgehaltenen anamnestischen Daten

Patients Anamnesis	SMA	Myositis	Hereditary Myopathies	Pompe disease	Control
**Diagnosis**	X	X	X	X	X
**Diagnostics**	X	X	X	X	
- Genetics	x		x	x	
- Muscle biopsy	x	x	x	x	
- Muscle MRI	x	x	x	x	
- Muscle ultrasound	x	x	x	x	
- EMG	x	x	x	x	
**Laborparameter**	X	X	X	X	X
- Transaminasen	x	x	x	x	x
- Auto-Antikörper		x			
**Ability to walk**	X	X	X	X	X
**Lung disease**	X	X	X	X	X
- Ventilation therapy	x	x	x	x	x
**Medication**	X	X	X	X	X

4. Study design und flow

Due to the lack of preliminary data regarding the planned investigations and the hypotheses formulated in this study, this is an exploratory study. There are no interventions within the study.

A comparison is made between non-myopathic control subjects and patients with various neuromuscular diseases, as well as a comparison of the results of the neuromuscular diseases with each other. The study protocol is largely identical between the different disease entities; the only differences are in the collection of disease-specific parameters/scores, which only make sense to collect from a medical perspective in the case of a specific disease. There are also differences in the documented anamnestic data, since, for example, genetic findings can only be documented in genetic diseases and autoantibodies can only be found in autoimmune-mediated inflammatory diseases.

Randomization:

There is no randomization. The division into groups is based on the known neuromuscular disease. Patients with primary neuronal (SMA) and inflammatory myopathy (myositis) as well as non-inflammatory myopathies (hereditary myopathies, Pompe disease) and subjects without the presence of a neuromuscular disease are included as non-myopathic controls.

Blinding:

Blinding for the examination is not possible due to the often clearly pronounced clinical phenotype. Blinding is carried out for the evaluation (gray scale analysis) and data analysis. Blinding the study participants is neither possible nor necessary as part of the planned study design.

Course of study:

Figure 1 shows the planned course of the study. The inclusion of test subjects and patients in the study can only take place after receiving written consent to participate in the study. The written declaration of consent requires oral and written information from the test subjects and patients about the aims and methods used of this study (including information about the scientific-exploratory nature of this study), the benefits and risks of participation in the study and a possible withdrawal consent. The information is provided via patient information sheets written in understandable language. By giving written consent, the test subjects and patients agree to the collection and storage of data relevant to the study. The study participant is informed that the declaration of consent can be withdrawn at any time without resulting in any disadvantages with regard to the treatment of the underlying disease. In addition, patients are informed that no benefits in terms of diagnostics or treatment are to be expected from participation in the study. The original of the consent form remains in the study center in a suitable location; the test subjects and patients receive a copy. Only people who are directly responsible for carrying out the study have access to the study data. Patient information and informed consent can be found in the appendix of this study protocol.

Translated from the original protocol

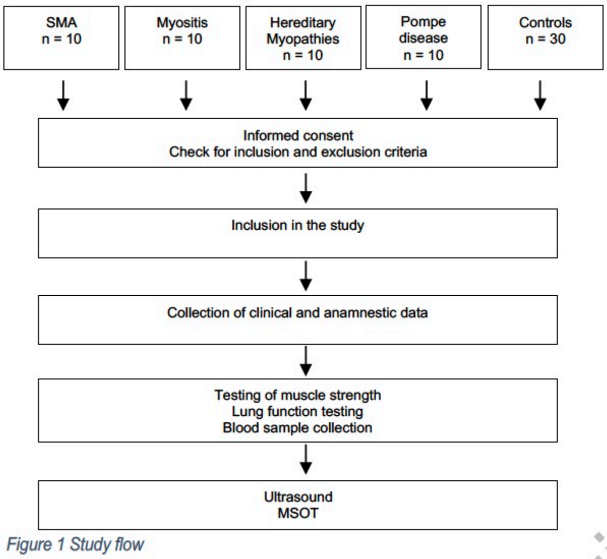

Figure 1 Study flow

After the test subjects and patients have been informed and written consent has been received, physical strength/muscle strength and associated clinical parameters (MRC, 6MWT, Timed to get up and go test) are recorded. Disease-specific scores are collected and a pulmonary function test is performed. The data mentioned on the course of the disease and preliminary findings (blood values, electrophysiology, imaging, muscle biopsy) are collected from the patient's electronic medical record or doctor's letters. A blood sample is taken to determine the hemoglobin value, the current CK and the HbA1c as parameters, which can have correlations to the muscle structure characteristics determined using MSOT and could thus contribute to influencing the MSOT data. The total amount of blood drawn will not exceed 40 ml. The lung function test is then carried out in the Pulmonology Clinic. The patients and test subjects are cared for by medical professionals who carry out these examinations daily as part of routine diagnostics.

The MSOT will then be performed in 7 anatomical regions on all study participants. These regions are the paraspinal muscles, the rectus abdominis, the upper and forearm, and the upper and lower leg. The examination is analogous to a sonographic examination of the corresponding regions over the corresponding skin areas and without invasive procedures. The anatomical region can be localized using a B-ultrasound mode built into the probe, and the corresponding optoacoustic signal can then be recorded. The duration per anatomical region is approximately 5 minutes. The duration of the examination is a maximum of 10 minutes for the paraspinal muscles trapezius and rectus abdominus muscles; a maximum of 20 minutes for both upper extremities as well as a maximum of 20 minutes for the examination of both lower extremities and 10 minutes examination time for the liver. The total examination time should therefore not exceed 60 minutes. The duration of exposure to the laser light used is significantly shorter and is approximately 1 minute per muscle region examined. The study participants lie in a relaxed position during the examination. Support of the examination by the test subjects or patients, e.g. i.S. Movement or breathing maneuvers are not necessary.

Time schedule:

For the individual study participant, the time required to participate in the study is approximately 175 minutes. Approximately 30 minutes are required for the comprehensive information,

Translated from the original protocol

explanation and consent of the test subjects and patients. The collection of clinical parameters including examination of muscle strength and filling out the questionnaires is estimated to take approximately 45 minutes. Carrying out the pulmonary function test takes approximately 20 minutes. The blood sample takes approximately 10 minutes. The B-mode ultrasound examination takes place in 10 minutes and the MSOT examination takes place in 60 minutes. The total duration of the study until the last test subject/patient is included will be approximately 36 months.

5. Recruitment of control subjects

We plan to include a maximum of 30 control subjects without underlying neuromuscular disease in this study as a non-myopathic control group. Since this is a pilot study, a sample size calculation based on preliminary statistical data is not possible in this case. Due to the innovative method, there is no sufficient data basis to extract representative test results from a healthy normal population from the literature. In particular, there is a lack of reliable studies on age and gender distribution that could be used for such an evaluation. In order to enable the planned adaptation of the test subjects and patient groups in terms of gender and age, the inclusion of a larger test subject pool (n = 30) compared to the patient groups (n = 10 each) is planned.

The recruitment of test subjects is adapted to the age and gender of the patient cohorts via the normal neurological wards as well as the neurological outpatient clinics and the day clinic of the Department of Neurology at the University Medical Center Göttingen. Patients presenting there with neurological diseases that do not fall into the spectrum of neuromuscular diseases can be considered as test subjects for this study; this includes, for example, patients with transient ischemic attacks (TIA), epilepsy, dizziness syndromes or headache syndromes. Acutely ill or unstable patients will not be considered for inclusion in this study. Persons with unclear neuromuscular symptoms will also be excluded from recruitment.
In order to obtain a control group that is optimally adapted to the included patient cohort in terms of age and gender, neuromuscularly healthy test subjects should also be recruited from among students, employees and visitors to the University and the University Medical Center Göttingen. The test subjects will be made aware of the study using a notice (see Appendix IX) in university buildings and university medicine.

Inclusion criteria:

- No presence of an underlying neuromuscular disease or corresponding symptoms
- ≥ 18 years
- Capable of giving consent to participate in the study

Exclusion criteria:

- Presence of an unclear neuromuscular disease or symptoms
- Presence of liver disease
- <18 years
- Restrictions on the ability to consent to participation in the study
- Pregnancy
- Tattoos on the skin in the examination area

Translated from the original protocol

As part of the study information, the test subjects are given the opportunity to document in writing whether they want to be informed about findings collected as part of the study examinations (clinical examination, blood sampling, lung function examination, muscle ultrasound). From our point of view, the possibility of receiving the results of the study examinations provides additional benefit for the participating test subjects, which contributes to increased motivation with regard to study participation and an optimization of the risk-benefit ratio for the test subjects. The test subjects can separately choose whether they only want to be informed about any suspicious

incidental findings detected during the study.

6. Recrutiment of patients

We plan to include 10 patients with SMA, 10 patients with myositis, 10 patients with hereditary myopathy and 10 patients with Pompe disease in this study. Since this is a pilot study, it is not possible to calculate the number of study participants based on preliminary statistical data. In estimating a number of 10 patients per disease group, we rely on the comparable studies from Erlangen examining juvenile patients with Duchenne muscular dystrophy or SMA.

The patients were recruited via the special outpatient clinic for neuromuscular diseases, the day clinic and the wards of the Department of Neurology at the University Medical Center Göttingen. The patients are specifically approached about taking part in the study at regular check-up appointments in the clinic. Patients are given sufficient time to ask any questions they may have about understanding the study process and the goals of the study. Acutely ill or unstable patients will not be included in the study, and only patients who are fully capable of providing informed consent and consent to participation in the study will be included in the study.

Inclusion criteria:

- Confirmed diagnosis of one of the neuromuscular diseases evaluated in the study
- ≥ 18 years
- Capable of giving consent to participate in the study

Exclusion criteria:

- Presence of unclear neuromuscular disease or a neuromuscular disease that is not to be investigated in this study
- <18 years
- Restrictions on the ability to consent to participation in the study
- Pregnancy
- Tattoos on the skin in the examination area

As part of the study information, patients are given the opportunity to document in writing whether they want to be informed about findings collected as part of the study examinations (clinical examination, blood sample, lung function test, muscle ultrasound). Patients can also choose whether they only want to be informed about any suspicious incidental findings detected during the study.

Translated from the original protocol

7. Benefit-risk anaylsis

Study-related risk (related to MSOT):
Based on the classification criteria for medical devices (Directive 93/42/EEC, Annex IX), the iThera Medical optoaucistic system is considered a Class IIa laser system:
- Active diagnostic device
- Non-invasive
- Temporary use (< 60 minutes)

A CE certification for this research device (current type designation according to imprint: Acuity Echo) is available. The information brochure for the device from the manufacturer, which contains the CE certification, is included in the appendix of this study protocol (Appendix V). There is no dependency relationship with the manufacturer; All diagnostic and analytical procedures are available to the study directors of the study center. The cooperation with the manufacturing company is recorded in a separate contract by the legal department before the study begins.

Compliance with energy limits:
Laser safety and maximum permitted radiation for irradiation with laser pulses is determined by the ANSI and IEC 60825 laser standards. The MSOT system adheres to these standards and remains below the MPE (maximum permissible exposure) limits for skin irradiation and is therefore considered safe.

Temperature change in tissues:
Optoacoustic imaging does not lead to a significant increase in tissue temperature. The absorption of the laser pulses in the tissue leads to a temporary local temperature increase of a few millikelvin. Depending on the duration of the examination and the patient's skin type, there is typically a temperature increase of less than one degree Kelvin.

Histological changes in the tissue:
Histological tissue changes in the target tissue or the surrounding structures are neither expected nor have they been observed in preclinical or clinical studies in the past. A slight, reversible warming or redness of the skin may occur in sensitive skin. Such a change can be noticed by the test subject or the examiner at any time, which can lead to the examination being interrupted or terminated. In any case, no irreversible damage is to be expected from this.

In principle, the near-infrared light used in MSOT can cause damage to the retina if the radiation hits the eye. To prevent this, the study participants and examiners wear suitable laser safety glasses during the examination.

Since the data collected is not used to interpret the findings, there is no risk of influence on the diagnosis (misdiagnosis) or incorrect presentation of the data in this exploratory pilot study.

There are no further risks in this study and were not identified in previous studies (Regensburger et al. 2019, 2021).

Translated from the original protocol

8. Biometrics

This study has an exploratory study design.

Case number calculation/Power calculation:

Since this is a pilot study and there is currently no information about the expected differences between the different groups, no statistical sample size calculation could be carried out. The given number of study participants is based on a study on MSOT imaging of muscles that was carried out at the University of Erlangen, in which the muscle tissue of patients with Duchenne muscular dystrophy and children with SMA was examined. Here the number of cases was sufficient to detect a difference to healthy test subjects. (Regensburger et al. 2019, Regensburger et al. 2021).

Statistical methods:

Continuous variables are reported as means with standard deviations; Categorical variables are reported as percentages when appropriate. The MSOT parameters are compared with each other using a two-tailed unpaired t-test (with Welch correction if necessary) or multifactorial ANOVA (MANOVA). ROC (Receiver Operator Characteristics) analyzes between disease groups are also planned. Correlations are examined using Pearson coefficients. All statistical tests are two-sided and set at a significance level of $p < 0.05$. All analyzes are carried out using GraphPad Prism (version 7.00 or later, GraphPad Software, La Jolla, CA, USA), RStudio (version 1.1.456 or later, RStudio Inc., Boston, MA, USA) or IBM SPSS Statistics (version 24 or current, IBM Corp., Armonk, NY, USA).

9. Ethical considerations, archiving and data protection

The study will be conducted in accordance with the current version of the Declaration of Helsinki. The detailed study protocol will be submitted to the ethics committee of the University Medical Centre Göttingen before the start of the study.

Patients will be informed about the study verbally and in writing before the start of the study. Their consent is documented by signing the consent form. The patient's consent to participate in the study is voluntary. Consent can be withdrawn at any time without giving reasons and without any disadvantages for further medical care.

The names of the patients and all other confidential information are subject to medical confidentiality, the provisions of the Lower Saxony Data Protection Act (NDSG) and the General Data Protection Regulation (EU GDPR). The data collected as part of the clinical study will be stored and analysed in pseudonymised form at the neurology clinic. During pseudonymisation, the patient's identification features are encrypted using a number or letter code; the pseudonymisation key is generated and stored at the neurology clinic's test centre.Both the pseudonymisation key and the data collected as part of this study can only be viewed by persons entrusted with conducting the study (investigators and study assistants).These persons are subject to a duty of confidentiality and are obliged to maintain data protection.Third parties are not granted access to original documents. The raw data of the MSOT imaging (no patient-related data) will be stored on specifically defined servers of iThera Medical GmbH. Forwarding of data in this study is exclusively intended for the raw data of the MSOT.
Forma iThera Medical GmbH will work with this data to ensure adequate recording quality and to develop analysis algorithms. The data will only be transmitted pseudonymised on encrypted physical data carriers. The co-operation will be specifically regulated by the legal department before the start of the study.

The data will be stored at the trial centre for at least ten years after completion or discontinuation

Translated from the original protocol

of the trial; in the event of revocation by the patient, all data collected up to that point and not yet anonymised will be deleted.

Patients and test subjects have the opportunity to document in writing on the consent form whether they wish to be informed about the study results. Information about incidental findings that may be detected during clinical testing, blood sampling, pulmonary function testing and muscle ultrasound will only be provided if the patients have explicitly agreed to be informed about this. A corresponding reference and the option to document the corresponding preferences in writing can be found in the patient information, the subject information and the consent forms.

Translated from the original protocol

10. Literature

Bembi B, Cerini E, Danesino C, Donati MA, Gasperini S, Morandi L, Musumeci O, Parenti G, Ravaglia S, Seidita F, et al. (2008): Management and treatment of glycogenosis type II. Neurology 71, S12-36

Bonati U, Holiga Š, Hellbach N, Risterucci C, Bergauer T, Tang W, Hafner P, Thoeni A, Bieri O, Gerlach I, et al. (2017): Longitudinal characterization of biomarkers for spinal muscular atrophy. Ann Clin Transl Neurol 4, 292– 304

Butchbach MER (2016): Copy Number Variations in the Survival Motor Neuron Genes: Implications for Spinal Muscular Atrophy and Other Neurodegenerative Diseases. Front Mol Biosci 3, 7

Carter JC, Sheehan DW, Prochoroff A, Birnkrant DJ (2018): Muscular Dystrophies. Clin Chest Med 39, 377–389 Case LE, Beckemeyer AA, Kishnani PS (2012): Infantile Pompe disease on ERT: update on clinical presentation, musculoskeletal management, and exercise considerations. Am J Med Genet C Semin Med Genet 160C, 69–79 Cassius C, Le Buanec H, Bouaziz J-D, Amode R (2019): Biomarkers in Adult Dermatomyositis: Tools to Help the Diagnosis and Predict the Clinical Outcome. J Immunol Res 2019

Claeys KG (2020): Congenital myopathies: an update. Dev Med Child Neurol 62, 297–302

Cupler EJ, Berger KI, Leshner RT, Wolfe GI, Han JJ, Barohn RJ, Kissel JT, AANEM Consensus Committee on Late- onset Pompe Disease (2012): Consensus treatment recommendations for late-onset Pompe disease. Muscle Nerve 45, 319–333

Danielsson O, Häggqvist B (2021): Skeletal muscle immunohistochemistry of acquired and hereditary myopathies. Curr Opin Rheumatol 33, 529–536

Dubowitz V (2009): Ramblings in the history of spinal muscular atrophy. Neuromuscul Disord 19, 69–73 EuroQol Group (1990): EuroQol--a new facility for the measurement of health-related quality of life. Health Policy 16, 199–208

Faravelli I, Meneri M, Saccomanno D, Velardo D, Abati E, Gagliardi D, Parente V, Petrozzi L, Ronchi D, Stocchetti N, et al. (2020): Nusinersen treatment and cerebrospinal fluid neurofilaments: An explorative study on Spinal Muscular Atrophy type 3 patients. J Cell Mol Med 24, 3034–3039

Figueroa-Bonaparte S, Segovia S, Llauger J, Belmonte I, Pedrosa I, Alejaldre A, Mayos M, Suárez-Cuartín G, Gallardo E, Illa I, et al. (2016): Muscle MRI Findings in Childhood/Adult Onset Pompe Disease Correlate with Muscle Function. PLOS ONE 11, e0163493

Finkel RS, McDermott MP, Kaufmann P, Darras BT, Chung WK, Sproule DM, Kang PB, Foley AR, Yang ML, Martens WB, et al. (2014): Observational study of spinal muscular atrophy type I and implications for clinical trials. Neurology 83, 810–817

Finkel RS, Mercuri E, Darras BT, Connolly AM, Kuntz NL, Kirschner J, Chiriboga CA, Saito K, Servais L, Tizzano E, et al. (2017): Nusinersen versus Sham Control in Infantile-Onset Spinal Muscular Atrophy. N Engl J Med 377, 1723–1732

Finkel RS, Mercuri E, Meyer OH, Simonds AK, Schroth MK, Graham RJ, Kirschner J, Iannaccone ST, Crawford TO, Woods S, et al. (2018): Diagnosis and management of spinal muscular atrophy: Part 2: Pulmonary and acute care; medications, supplements and immunizations; other organ systems; and ethics. Neuromuscul Disord 28, 197–207

Foust KD, Wang X, McGovern VL, Braun L, Bevan AK, Haidet AM, Le TT, Morales PR, Rich MM, Burghes AHM, Kaspar BK (2010): Rescue of the spinal muscular atrophy phenotype in a mouse model by early postnatal delivery of SMN. Nat Biotechnol 28, 271–274

Gruhn KM, Heyer CM, Güttsches A-K, Rehmann R, Nicolas V, Schmidt-Wilcke T, Tegenthoff M, Vorgerd M, Kley RA (2015): Muscle imaging data in late-onset Pompe disease reveal a correlation between the pre-existing degree of lipomatous muscle alterations and the efficacy of long-term enzyme replacement therapy. Mol Genet Metab Rep 3, 58–64

Hers HG (1963): α-Glucosidase deficiency in generalized glycogen-storage disease (Pompe's disease). Biochem J
86, 11–16

Kariyawasam DST, D'Silva A, Lin C, Ryan MM, Farrar MA (2019): Biomarkers and the Development of a
Personalized Medicine Approach in Spinal Muscular Atrophy. Front Neurol 10, 898

Translated from the original protocol

Keller CW, Schmidt J, Lünemann JD (2017): Immune and myodegenerative pathomechanisms in inclusion body myositis. Ann Clin Transl Neurol 4, 422–445

Kishnani PS, Howell RR (2004): Pompe disease in infants and children. J Pediatr 144, S35-43

Kishnani PS, Hwu W-L, Mandel H, Nicolino M, Yong F, Corzo D, Infantile-Onset Pompe Disease Natural History Study Group (2006a): A retrospective, multinational, multicenter study on the natural history of infantile-onset Pompe disease. J Pediatr 148, 671–676

Kishnani PS, Steiner RD, Bali D, Berger K, Byrne BJ, Case L, Crowley JF, Downs S, Howell RR, Kravitz RM, et al. (2006b): Pompe disease diagnosis and management guideline. Genet Med 8, 267–288

Kishnani PS, Corzo D, Nicolino M, Byrne B, Mandel H, Hwu WL, Leslie N, Levine J, Spencer C, McDonald M, et al. (2007): Recombinant human acid [alpha]-glucosidase: major clinical benefits in infantile-onset Pompe disease. Neurology 68, 99–109

Knieling F, Neufert C, Hartmann A, Claussen J, Urich A, Egger C, Vetter M, Fischer S, Pfeifer L, Hagel A, et al.

(2017): Multispectral Optoacoustic Tomography for Assessment of Crohn's Disease Activity. N Engl J Med 376, 1292–1294

Lefebvre S, Bürglen L, Reboullet S, Clermont O, Burlet P, Viollet L, Benichou B, Cruaud C, Millasseau P, Zeviani M (1995): Identification and characterization of a spinal muscular atrophy-determining gene. Cell 80, 155–165 Mailman MD, Heinz JW, Papp AC, Snyder PJ, Sedra MS, Wirth B, Burghes AHM, Prior TW (2002): Molecular analysis of spinal muscular atrophy and modification of the phenotype by SMN2. Genet Med 4, 20–26 Mendell JR, Al-Zaidy S, Shell R, Arnold WD, Rodino-Klapac LR, Prior TW, Lowes L, Alfano L, Berry K, Church K, et al. (2017): Single-Dose Gene-Replacement Therapy for Spinal Muscular Atrophy. N Engl J Med 377, 1713–1722 Mendell JR, Al-Zaidy SA, Rodino-Klapac LR, Goodspeed K, Gray SJ, Kay CN, Boye SL, Boye SE, George LA, Salabarria S, et al. (2021): Current Clinical Applications of In Vivo Gene Therapy with AAVs. Mol Ther 29, 464– 488

MUNSAT T (1991): Workshop report. International SMA Collaboration. Neuromuscul Disord 1, 81

Oldfors A (2007): Hereditary myosin myopathies. Neuromuscul Disord 17, 355–367

Pearn JH, Carter CO, Wilson J (1973): The genetic identity of acute infantile spinal muscular atrophy. Brain 96, 463–470

Preisler N, Lukacs Z, Vinge L, Madsen KL, Husu E, Hansen RS, Duno M, Andersen H, Laub M, Vissing J (2013): Late-onset Pompe disease is prevalent in unclassified limb-girdle muscular dystrophies. Mol Genet Metab 110, 287–289

Regensburger AP, Fonteyne LM, Jüngert J, Wagner AL, Gerhalter T, Nagel AM, Heiss R, Flenkenthaler F, Qurashi M, Neurath MF, et al. (2019): Detection of collagens by multispectral optoacoustic tomography as an imaging biomarker for Duchenne muscular dystrophy. Nat Med 25, 1905–1915

Regensburger AP, Brown E, Krönke G, Waldner MJ, Knieling F (2021): Optoacoustic Imaging in Inflammation. Biomedicines 9, 483

Regensburger AP, Wagner AL, Danko V, Jüngert J, Federle A, Klett D, Schuessler S, Buehler A, Neurath MF, Roos A, et al. (2022): Multispectral optoacoustic tomography for non-invasive disease phenotyping in pediatric spinal muscular atrophy patients. Photoacoustics 25, 100315

Rigo F, Hua Y, Krainer AR, Bennett CF (2012): Antisense-based therapy for the treatment of spinal muscular atrophy. J Cell Biol 199, 21–25

Ryder S, Leadley RM, Armstrong N, Westwood M, de Kock S, Butt T, Jain M, Kleijnen J (2017): The burden, epidemiology, costs and treatment for Duchenne muscular dystrophy: an evidence review. Orphanet J Rare Dis 12, 79

Schmidt J (2018): Current Classification and Management of Inflammatory Myopathies. J Neuromuscul Dis 5, 109–129

Sivaramakrishnan M, McCarthy KD, Campagne S, Huber S, Meier S, Augustin A, Heckel T, Meistermann H, Hug MN, Birrer P, et al. (2017): Binding to SMN2 pre-mRNA-protein complex elicits specificity for small molecule splicing modifiers. Nat Commun 8, 1476

Slonim AE, Bulone L, Ritz S, Goldberg T, Chen A, Martiniuk F (2000): Identification of two subtypes of infantile acid maltase deficiency. J Pediatr 137, 283–285

Valori CF, Ning K, Wyles M, Mead RJ, Grierson AJ, Shaw PJ, Azzouz M (2010): Systemic delivery

Translated from the original protocol

of scAAV9 expressing SMN prolongs survival in a model of spinal muscular atrophy. Sci Transl Med 2, 35ra42

van den Hout HMP, Hop W, van Diggelen OP, Smeitink JAM, Smit GPA, Poll-The B-TT, Bakker HD, Loonen MCB, de Klerk JBC, Reuser AJJ, van der Ploeg AT (2003): The natural course of infantile Pompe's disease: 20 original cases compared with 133 cases from the literature. Pediatrics 112, 332–340

Translated from the original protocol

Vandervelde L, Van den Bergh PYK, Goemans N, Thonnard J-L (2007): ACTIVLIM: a Rasch-built measure of activity limitations in children and adults with neuromuscular disorders. Neuromuscul Disord 17, 459–469
Vill K, Kölbel H, Schwartz O, Blaschek A, Olgemöller B, Harms E, Burggraf S, Röschinger W, Durner J, Gläser D, et al. (2019): One Year of Newborn Screening for SMA - Results of a German Pilot Project. J Neuromuscul Dis 6, 503–515
Waldner MJ, Knieling F, Egger C, Morscher S, Claussen J, Vetter M, Kielisch C, Fischer S, Pfeifer L, Hagel A, et al. (2016): Multispectral Optoacoustic Tomography in Crohn's Disease: Noninvasive Imaging of Disease Activity. Gastroenterology 151, 238–240
Wirth B, Karakaya M, Kye MJ, Mendoza-Ferreira N (2020): Twenty-Five Years of Spinal Muscular Atrophy Research: From Phenotype to Genotype to Therapy, and What Comes Next. Annu Rev Genomics Hum Genet 21, 231–261

Translated from the original protocol

Appendix

I. Control subject information
II. Patient information
III. Declaration of consent from control subjects
IV. Declaration of consent from patients
V. Manufacturer information Acuity Echo
VI. Current curriculum vitae of the director of studies
VII. Ethics application from the Erlangen Children's Hospital for the comparable MSOT study
VIII. Positive ethics vote from the Erlangen Ethics Commission
IX. Appendix for subject recruitmen

Translated from the original protocol

Translated from the original protocol

5. Statistical Analysis Plan (SAP)

Statistical Analysis Plan	
Clinical Investigation Plan Title	Multi**sp**ectral **O**ptoacoustic **T**omography for Translational Imaging in **P**ompe **D**isease (SPOT_PD)
Clinicaltrial.gov Identifier	NCT05083806
Clinical Investigation Plan Version	V1.4
Sponsor	IIT (Grant funding: Sanofi Aventis)
Document Version	SAP v1.1
**Confidentiality Statement**<br><br>All the information contained in this document is confidential.	

Inhaltsverzeichnis

1. VERSION HISTORY 3
2. LIST OF ABBREVIATIONS AND DEFINITIONS OF TERMS 3
3. INTRODUCTION 4
4. STUDY OBJECTIVES AND ENDPOINTS 5
5. INVESTIGATION PLAN 8
6. DETERMINATION OF SAMPLE SIZE 10
7. TARGET PARAMETERS 10
7. STATISTICAL METHODS 12
7.1. STUDY SUBJECTS 12
7.2. CLINICAL INVESTIGATION PLAN (CIP) DEVIATIONS 12
7.3 ANALYSIS SETS 12
7.4 GENERAL METHODOLOGY 12
7.5 HANDLING OF MISSING DATA AND DROPOUTS 12
7.6 SAFETY EVALUATION 12

1. Version History

Version	Changes	Authors, Title
v1.0	SAP generated (11/06/2021)	Adrian P. Regensburger, M.D.<br>Ferdinand Knieling, M.D.
v.1.1	DMD arm canceled (30/05/2023)	Ferdinand Knieling, M.D.

2. List of Abbreviations and Definitions of Terms

Abbreviation	Definition
OAI	Optoacoustic imaging
MSOT	Multispectral optoacoustic tomography
HFMSE	expanded Hammersmith functional motor scale
ULM/RULM	Revised Upper Limb Module
R-PACT	The Rasch-built Pompe-specific activity scale
6-MWT	6-minute walking test
RUCT	reflected ultrasound computed tomography
ROC	Receiver operator characteristics
HV	healthy volunteers
PD	Pompe disease

3. Introduction

As a new imaging modality, optoacoustic imaging (OAI) combines benefits of optical (high contrast) and acoustic (high resolution) imaging. Multispectral optoacoustic tomography (MSOT) is therefore capable of visualizing the distribution of endogenous absorbers by initiating laser-induced thermoelastic expansion and detection of resulting pressure waves. This imaging technique enables the label-free detection and quantification of different endogenous chromophores, such as melanin, hemoglobin, deoxyhemoglobin and lipids.

Pompe disease (PD) is an ultra-rare autosomal-recessive glycogen storage resulting in proximal muscle weakness and loss of respiratory function. While enzyme replacement therapy (ERT) is the only effective treatment, biomarkers for disease monitoring are scarce. In this study we want to refine the capability of MSOT to characterize muscle tissue and to determine a non-invasive, quantitative biomarker for the disease assessment in PD patients using MSOT.

4. Study Objectives and endpoints

The study population consists of patients with Pompe disease patients and healthy volunteers.

Objectives

Primary objective

- Comparison of the optoacoustic spectrum determined by MSOT in patients with PD compared to healthy volunteers, generating a new biomarker for disease monitoring in PD

Secondary outcomes:

- Comparison of the quantitative glycogen signal fraction determined by MSOT in patients with PD compared to healthy volunteers
- Comparison of the quantitative lipid signal fraction determined by MSOT in patients with PD compared to healthy volunteers
- Comparison of the quantitative fraction of collagen signal determined by MSOT in patients with PD compared to healthy volunteers
- Comparison of the quantitative fraction of hemo-/myoglobin signal determined by MSOT in patients with PD compared to healthy volunteers
- Comparison of the quantitative fraction of oxygenated/deoxygenated hemoglobin determined by MSOT in patients with PD compared to healthy volunteers
- Correlation of glycogen content determined with MSOT with disease duration/patient age
- Correlation of lipid content determined with MSOT with disease duration/patient age
- Correlation of collagen determined by MSOT with disease duration/patient age
- Correlation of haemoglobin/myoglobin content determined by MSOT with duration of disease/patient age
- Correlation of oxygenated/deoxygenated hemoglobin determined by MSOT with duration of disease / patient age
- Correlation of glycogen content determined with MSOT with R-Pact scale
- Correlation of lipid content determined with MSOT with R-PAct scale
- Correlation of collagen determined by MSOT with R-PAct scale
- Correlation of haemoglobin/myoglobin content determined by MSOT with R-PAct scale
- Correlation of oxygenated/deoxygenated hemoglobin determined by MSOT with R-PAct scale

- Correlation of glycogen content determined with MSOT with age-related functional muscle tests (Revised Upper Limb Module (RULM)/6-Minute-Walk Test (6-MWT)/Time-to-go-up-and-go-test/MRC Muscle Strength Grades)
- Correlation of lipid determined with MSOT with age-dependent functional muscle tests (RULM /6-MWT/Time-to-get-up-and-go/MRC)
- Correlation of collagen determined with MSOT with age-dependent functional muscle tests (RULM/6-MWT/Time-to-get-up-and-go/MRC)
- Correlation of hemo-/myoglobin content determined with MSOT with age-dependent functional muscle tests (RULM/6-MWT/Time-to-get-up-and-go/MRC)
- Correlation of oxygenated/deoxygenated hemoglobin determined with MSOT with age-related functional muscle tests (RULM /6-MWT/Time-to-get-up-and-go/MRC)
- Correlation of glycogen content determined with MSOT with B-mode ultrasound (Heckmatt scale/Echogenity/Gray Scale Level/UGAP)
- Correlation of lipid determined with MSOT with B-mode ultrasound (Heckmatt scale/Echogenity/Gray Scale Level/UGAPI)
- Correlation of collagen determined with B-mode ultrasound (Heckmatt scale/Echogenity/Gray Scale Level/UGAP)
- Correlation of hemo-/myoglobin content determined with MSOT with B-mode ultrasound (Heckmatt scale/Echogenity/Gray Scale Level/UGAP)
- Correlation of oxygenated/deoxygenated hemoglobin determined with B-mode ultrasound (Heckmatt scale/Echogenity/Gray Scale Level/UGAP)
- Correlation of glycogen content determined with MSOT with respiratory function tests (Spirometry)
- Correlation of lipid content determined with MSOT with respiratory function tests (Spirometry)
- Correlation of collagen determined by MSOT with respiratory function tests (Spirometry)
- Correlation of haemoglobin/myoglobin content determined by MSOT with respiratory function tests (Spirometry)
- Correlation of oxygenated/deoxygenated hemoglobin determined by MSOT with respiratory function tests (Spirometry)
- Measurement of signal differences in right / left comparison
- Correlation of glycogen content determined with MSOT with (functional) magnetic resonance imaging parameters
- Correlation of lipid determined with MSOT with (functional) magnetic resonance imaging parameters

- Correlation of collagen determined with (functional) magnetic resonance imaging parameters
- Correlation of hemo-/myoglobin content determined with (functional) magnetic resonance imaging parameters
- Correlation of oxygenated/deoxygenated hemoglobin determined with (functional) magnetic resonance imaging parameters

Primary endpoint:
Optoacoustic Absorption Spectrum of Muscle and liver in PD.
This target is measured non-invasively by MSOT.

Secondary endpoints:
Quantitative glycogen signal (in arbitrary units)
Quantitative lipid signal (in arbitrary units)
Quantitative collagen signal (in arbitrary units)
Quantitative hemo/myoglobin signal (in arbitrary units)
Muscle oxygenation (in %)
These target values are measured non-invasively by MSOT.
B-mode ultrasound

- Heckmatt scale
- Echogenitiy
- Gray Scale Level
- Ultrasound-Guided Attentuation Parameter (UGAP)

R-PaAt scale

- Summed raw score

Respiratory function test

- FVC
- FEV1

Functional magnetic resonance imaging of lung.

- Ventilation Defects / Non-defected
- Perfusion Defects / Non-defected
- Combined Defects / Non-defected

Magnetic resonance imaging of biceps muscle.

Correlation of glycogen detected by MSOT with clinical scores for determining muscle strength consisting of:

- Revised Upper Limb Module (RULM)
- MRC Muscle Strength Grades

in case of ability to walk additionally:

- 6-minute walking test
- Time-to-get-up-and-go-test

These target values are clinically determined at presentation (electronic patient record, physician letters):
Age

Sex

Weight

Skin color

Ethnic Background

Disease duration

Current medication

Results of last biopsy (if available)

Results of last MRI (if available)

5. Investigation Plan

- This is a mono-centric, open-labeled study, which aims to compare the optoacoustic spectra and optoacoustic signals between healthy volunteers (HV) and PD patients.
- The study will include 10 HV and 10 PD patients.
- Each subject will be examined by MSOT, ultrasound and physical examinations. Clinical data will be obtained from the medical record and during the study.

Inclusion criteria:

PD patients (LOPD)

- Confirmed diagnosis of Pompe disease
- From 18 years of Age
- Independent from current therapy

Healthy controls

- From 18 years of Age, matched (age, gender) to PD collective

Exclusion criteria

PD patients

- Pregnancy
- Tattoo on skin to be examined

Healthy controls

- Anamnestic of other signs of myopathy or liver disease
- Pregnancy
- Tattoo on skin to be examined

Patient/control number

As this is a pilot study, an exact case number calculation is not possible. It is planned to study a total of 10 patients with PD and 10 healthy controls.

6. Determination of Sample Size

As this is a pilot study and no information is available on the expected differences between the different groups, no case number calculation was carried out. The number of cases given represents an estimate or is within reasonable limits for a pilot study. The patients with diagnosed PD were compared to sex- and age-matched HV.

7. Target parameters

These target values are assessed at a single visit:

Study Parameters	PD	HV
MSOT	X	X
-'Glycogen'	X	X
-Lipid	X	X
-Collagen	X	X
-Hemoglobin oxygenated	X	X
-Hemoglobin deoxygenated	X	X
Ultrasound	X	X
-Heckmatt	X	X
-Echogenity	X	X
-Muscle texture	X	X
-Grey scale level	X	X
-UGAP	X	X
Magnetic resonance imaging	X	X
-Ventilation defects	X	X
-Perfusion defects	X	X
-Combined defects	X	X
-Biceps muscle imaging	x	
R-Pact scale (≥16y)	X	X
Respiratory function test	X	X
-FVC	X	X
-FEV1	X	X
Muscle tests	X	X
-RULM	X	X
-6-MWT	X	X
-Time-to-get-up-and-go	X	X
-MRC	X	X

These target values are clinically determined at the visit:

Patient History		
Confirmed Disease	X<br>Type:<br>Crim:	X
Diagnostics:<br>-Genetics<br>-Dried Blood Spot<br>-Urinary Glc 4<br>-Lymphozytes	X<br>X<br>X<br>X<br>X	
Muscle biopsy	X	
Neurological tests:<br>-EMG<br>-MRI<br>-Sleep Study	X<br>X<br>X<br>X	
Lab values<br>-CK<br>-Liver enzymes	X<br>X<br>X	
Sitting ability<br>Walking ability	X<br>X	
Ventilation	X	
Medication		

7. Statistical Methods

7.1. Study Subjects

We will describe all screened and enrolled patients.

7.2. Clinical Investigation Plan (CIP) Deviations

Data will be analyzed according to the SAP; any further/additional/deviation from the SAP will be reported as such. Further post-hoc analysis will be performed, if necessary.

7.3 Analysis Sets

One analysis set will be created for study purpose.

7.4 General Methodology

Continuous variables are given as means and standard deviations; categorical variables are provided as numbers and percentages. If appropriate, descriptive statistics will be provided using Tables.

MSOT spectra are compared between HV and PD patients. An area under the curve (AUC) of individuals/per group spectra will be compared. Furthermore, single wavelength of individuals/groups will be compared.

Between group analyses regarding unmixed and single wavelength MSOT signals will be analyzed as follows: Data are tested for normal distribution using Shapiro-Wilk test prior to inferential analysis. MSOT signals are compared between cohorts in a pairwise manner (matched for age) using dependent samples t-tests. If the assumption of normal distribution is violated Wilcoxon signed-rank tests is used. Receiver operator characteristics (ROC) analysis between muscles of HV and PD-patients is performed. As gold standard genotyping/enzymatic resting is used. All inferential tests are two-tailed, p values ≤ 0.05 are considered statistically significant. Bonferroni-Holm adjustment is used to control type I error, if appropriate. All analyses are performed using GraphPad Prism (Version 10.0 or newer, GraphPad Software, La Jolla, CA, USA).

7.5 Handling of Missing Data and Dropouts

No method of imputation will be used for missing data.

7.6 Safety Evaluation

During the study adverse events and serious adverse events will be monitored. The investigator is available for study subjects at any time, in case of any events.
