# Supplementary material for: Non-invasive optoacoustic imaging of glycogen-storage and muscle degeneration in late-onset Pompe disease
Source: Nat Commun. 2024 Sep 8;15:7843. doi: 10.1038/s41467-024-52143-6 (PMC11381542; doi:10.1038/s41467-024-52143-6)
Supplement: Supplementary file 3 — Reporting Summary [file 41467_2024_52143_MOESM3_ESM.pdf]

nature portfolio

Corresponding author(s): Knieling, Ferdinand

Last updated by author(s): Aug 10, 2024

Reporting Summary

Nature Portfolio wishes to improve the reproducibility of the work that we publish. This form provides structure for consistency and transparency in reporting. For further information on Nature Portfolio policies, see our Editorial Policies and the Editorial Policy Checklist.

Statistics

For all statistical analyses, confirm that the following items are present in the figure legend, table legend, main text, or Methods section.

n/a	Confirmed	
☐	☒	The exact sample size (*n*) for each experimental group/condition, given as a discrete number and unit of measurement
☐	☒	A statement on whether measurements were taken from distinct samples or whether the same sample was measured repeatedly
☐	☒	The statistical test(s) used AND whether they are one- or two-sided<br>*Only common tests should be described solely by name; describe more complex techniques in the Methods section.*
☐	☒	A description of all covariates tested
☐	☒	A description of any assumptions or corrections, such as tests of normality and adjustment for multiple comparisons
☐	☒	A full description of the statistical parameters including central tendency (e.g. means) or other basic estimates (e.g. regression coefficient) AND variation (e.g. standard deviation) or associated estimates of uncertainty (e.g. confidence intervals)
☐	☒	For null hypothesis testing, the test statistic (e.g. *F*, *t*, *r*) with confidence intervals, effect sizes, degrees of freedom and *P* value noted<br>*Give P values as exact values whenever suitable.*
☒	☐	For Bayesian analysis, information on the choice of priors and Markov chain Monte Carlo settings
☒	☐	For hierarchical and complex designs, identification of the appropriate level for tests and full reporting of outcomes
☐	☒	Estimates of effect sizes (e.g. Cohen's *d*, Pearson's *r*), indicating how they were calculated

Our web collection on statistics for biologists contains articles on many of the points above.

Software and code

Policy information about availability of computer code

Data collection	Data was collected in Microsoft Excel (Microsoft Corporation, Redmond, WA ,USA)
Data analysis	Data was analyzed using viewMSOT software (version 4.1, iThera Medical GmbH, Munich, Germany), iLabs software (version 1.3.16, iThera Medical GmbH, Munich, Germany), and Fiji software ImageJ software (V2.1.0/1.53c). All statistical analyses were performed using GraphPad Prism 10 (Version 10.1.0, Graphpad Software Inc.; San Diego, CA, USA).<br>For 3D phantom printing Autodesk Fusion 360 (V2.0.14567, Autodesk GmbH, München, Germany) was used.

For manuscripts utilizing custom algorithms or software that are central to the research but not yet described in published literature, software must be made available to editors and reviewers. We strongly encourage code deposition in a community repository (e.g. GitHub). See the Nature Portfolio guidelines for submitting code & software for further information.

Data

Policy information about availability of data

All manuscripts must include a data availability statement. This statement should provide the following information, where applicable:

- Accession codes, unique identifiers, or web links for publicly available datasets
- A description of any restrictions on data availability
- For clinical datasets or third party data, please ensure that the statement adheres to our policy

Due to the rarity of the disease and thus possible identifiability of individual patients, the datasets are only accessible with appropriate consent within the

framework of legal regulations from the corresponding author. The study does not report any original code.

Research involving human participants, their data, or biological material

Policy information about studies with human participants or human data. See also policy information about sex, gender (identity/presentation), and sexual orientation and race, ethnicity and racism.

Reporting on sex and gender	In each group, 5 [50 %] subjects were females. Gender was self-reported.
Reporting on race, ethnicity, or other socially relevant groupings	Such grouping has not been used/not relevant to the study.
Population characteristics	The mean age±SD was 41.2 ± 14.2 years in HV compared to 40.6 ± 12.1 years in the LOPD patients' cohort.
Recruitment	For main study: prospective, monocentric clinical study (UHE)<br>Patients (and parents) were informed about the possibility of participating in the study in the context of an elective presentation at the Clinic for Pediatrics and Adolescent Medicine (Neuropediatrics) and the Clinic for Neurology at the University Hospital in Erlangen, Germany as well as the the Clinic for Pediatrics and Adolescent Medicine (Neuropediatrics) in Gießen, Germany. Additional recruitment options include the Universtiy Medical Center Göttingen, University Hospital Halle, Clinic Rummelsberg, Schwarzenbruck, Germany, the German Society for muscular diseases (DGM), the treatNMD network and the international Pompe Association. For the acutal investigations, the patient were then referred to Erlangen.<br>Healthy volunteers wererecruited in the outpatient departments of the Clinic for Neurology at the University Hospital in Erlangen, Germany.<br>Critical ill or unstable patients were not recruited.<br><br>For the interdevice, -center and –examiner comparison: second center (UMG)
Ethics oversight	Main study: Ethics Committee of the University Hospital Erlangen (UHE), Germany (reference: 21-238_1-B)<br>Second center comparison: Ethics committee of the University Medical Center Göttingen (UMG), Germany (reference: 2/7/22)

Note that full information on the approval of the study protocol must also be provided in the manuscript.

Field-specific reporting

Please select the one below that is the best fit for your research. If you are not sure, read the appropriate sections before making your selection.

☒ Life sciences ☐ Behavioural & social sciences ☐ Ecological, evolutionary & environmental sciences

For a reference copy of the document with all sections, see nature.com/documents/nr-reporting-summary-flat.pdf

Life sciences study design

All studies must disclose on these points even when the disclosure is negative.

Sample size	As this is a pilot study and no information is available on the expected differences between the different groups, no case number calculation was carried out. The number of cases given represents an estimate or is within reasonable limits for a pilot study. . The patients with diagnosed PD were compared to sex- and age-matched HV.
Data exclusions	There were no data excluded. Only a single patient could not complete all physical tests due to physical impairments.
Replication	Ex and in vivo imaging experiments were at least performed in duplicates, and all attempts were successful. Human MSOT scanning was performed at multiple anatomic locations and validation with an external (independent dataset).
Randomization	There was no randomization. Subjects were assigned to group according to disease pathology.
Blinding	iven the variability of the clinical phenotype, blinding was not fully feasible for all patients for clinical testing, ultrasound, and MSOT. Muscle MRI was performed in a blinded fashion.

Reporting for specific materials, systems and methods

We require information from authors about some types of materials, experimental systems and methods used in many studies. Here, indicate whether each material, system or method listed is relevant to your study. If you are not sure if a list item applies to your research, read the appropriate section before selecting a response.

Materials & experimental systems

n/a	Involved in the study
x	Antibodies
x	Eukaryotic cell lines
x	Palaeontology and archaeology
x	Animals and other organisms
	x Clinical data
x	Dual use research of concern
x	Plants

Methods

n/a	Involved in the study
x	ChIP-seq
x	Flow cytometry
x	MRI-based neuroimaging

Clinical data

Policy information about clinical studies

All manuscripts should comply with the ICMJEguidelines for publication of clinical research and a completedCONSORT checklist must be included with all submissions.

Clinical trial registration

clinicaltrials.gov ID NCT05083806

Study protocol

Documents are available on clinicaltrials.gov

Data collection

A prospective, monocentric clinical study was conducted after receiving approval by the local ethics committee of the University Hospital Erlangen (UHE). The entire assessment was completing within a single visit. Study dates: May 17, 2022 to March 30, 2023.

Outcomes

As per protocol, primary outcome was:
Comparison of the optoacoustic spectrum determined by MSOT in patients with PD compared to healthy volunteers, generating a new biomarker for disease monitoring in PD.
Secondary outcomes:

- Comparison of the quantitative glycogen signal fraction determined by MSOT in patients with PD compared to healthy volunteers
- Comparison of the quantitative lipid signal fraction determined by MSOT in patients with PD compared to healthy volunteers
- Comparison of the quantitative fraction of collagen signal determined by MSOT in patients with PD compared to healthy volunteers
- Comparison of the quantitative fraction of hemo-/myoglobin signal determined by MSOT in patients with PD compared to healthy volunteers
- Comparison of the quantitative fraction of oxygenated/deoxygenated hemoglobin determined by MSOT in patients with PD compared to healthy volunteers
- Correlation of glycogen content determined with MSOT with disease duration/patient age
- Correlation of lipid content determined with MSOT with disease duration/patient age
- Correlation of collagen determined by MSOT with disease duration/patient age
- Correlation of haemoglobin/myoglobin content determined by MSOT with duration of disease/patient age
- Correlation of oxygenated/deoxygenated hemoglobin determined by MSOT with duration of disease / patient age
- Correlation of glycogen content determined with MSOT with R-Pact scale
- Correlation of lipid content determined with MSOT with R-PAct scale
- Correlation of collagen determined by MSOT with R-PAct scale
- Correlation of haemoglobin/myoglobin content determined by MSOT with R-PAct scale
- Correlation of oxygenated/deoxygenated hemoglobin determined by MSOT with R-PAct scale
- Correlation of glycogen content determined with MSOT with age-related functional muscle tests (Hammersmith Infant Neurological Examination (HINE)/The Children's Hospital of Philadelphia Infant Test of Neuromuscular Disorders (CHOP Intend)/expanded Hammersmith functional motor scale (HFMSE)/ Revised Upper Limb Module (RULM)/6-Minute-Walk Test (6-MWT)/Time-to-go-up-and-go-test/MRC Muscle Strength Grades)
- Correlation of lipid determined with MSOT with age-dependent functional muscle tests (RULM /6-MWT/Time-to-get-up-and-go/MRC)
- Correlation of collagen determined with MSOT with age-dependent functional muscle tests (RULM/6-MWT/Time-to-get-up-and-go/MRC)
- Correlation of hemo-/myoglobin content determined with MSOT with age-dependent functional muscle tests (RULM/6-MWT/Time-to-get-up-and-go/MRC)
- Correlation of oxygenated/deoxygenated hemoglobin determined with MSOT with age-related functional muscle tests (RULM /6-MWT/Time-to-get-up-and-go/MRC)
- Correlation of glycogen content determined with MSOT with B-mode ultrasound (Heckmatt scale/Echogenity/Gray Scale Level/UGAP)
- Correlation of lipid determined with MSOT with B-mode ultrasound (Heckmatt scale/Echogenity/Gray Scale Level/UGAPl)
- Correlation of collagen determined with B-mode ultrasound (Heckmatt scale/Echogenity/Gray Scale Level/UGAP)
- Correlation of hemo-/myoglobin content determined with MSOT with B-mode ultrasound (Heckmatt scale/Echogenity/Gray Scale Level/UGAP)
- Correlation of oxygenated/deoxygenated hemoglobin determined with B-mode ultrasound (Heckmatt scale/Echogenity/Gray Scale Level/UGAP)
- Correlation of glycogen content determined with MSOT with respiratory function tests (Spirometry)
- Correlation of lipid content determined with MSOT with respiratory function tests (Spirometry)
- Correlation of collagen determined by MSOT with respiratory function tests (Spirometry)

• Correlation of haemoglobin/myoglobin content determined by MSOT with respiratory function tests (Spirometry)
• Correlation of oxygenated/deoxygenated hemoglobin determined by MSOT with respiratory function tests (Spirometry)
• Measurement of signal differences in right / left comparison

• Correlation of glycogen content determined with MSOT with (functional) magnetic resonance imaging parameters
• Correlation of lipid determined with MSOT with (functional) magnetic resonance imaging parameters
• Correlation of collagen determined with (functional) magnetic resonance imaging parameters
• Correlation of hemo-/myoglobin content determined with (functional) magnetic resonance imaging parameters
• Correlation of oxygenated/deoxygenated hemoglobin determined with (functional) magnetic resonance imaging parameters

Plants

Seed stocks	n/a
Novel plant genotypes	n/a
Authentication	n/a
