# Supplementary material for: Non-invasive optoacoustic imaging of glycogen-storage and muscle degeneration in late-onset Pompe disease
Source: Nat Commun. 2024 Sep 8;15:7843. doi: 10.1038/s41467-024-52143-6 (PMC11381542; doi:10.1038/s41467-024-52143-6)
Supplement: Supplementary file 4 — Study protocol (Center 1, updated) [file 41467_2024_52143_MOESM4_ESM.pdf]

Study protocol

SPOT_PD

Multi**sp**ectral **O**ptoacoustic **T**omography for Translational Molecular Imaging in **P**ompe **D**isease

Content is confidential.

1. Table of contents

1. Table of contents 2
2. Study titel, version number, version date 3
3. Project summary 4
4. Project summary (German) 5
5. Responsabilities 6
6. Scientific background 7
7. Study objective 12
8. Endpoint parameters 15
9. Study design 18
10. Study population 20
11. Study flow 21
12. Benefit-risk-assessment 24
13. Biometrics 27
14. Data management and protection 27
15. Biomaterial handling 28
16. Individual paticipant insurance 28
17. Signatures 29

Content is confidential.

2. Study titel, version number, version date

Study title

Multispectral Optoacoustic Tomography for Translational Molecular Imaging in Pompe Disease

Version number

Version 1.3

Version date

30.05.2023

Protocol versions

Date	Version	Status	Changes
03.01.2020	1.0	Outline	
11.06.2021	1.1	Final	
05.08.2021	1.2	Revision	Minor arm excluded
02.03.2022	1.3	Amendment	MRI, UGAP included
30.05.2023	1.4	Amendment	DMD arm canceled

Content is confidential.

3. Project summary

In patients with Pompe disease (PD) a progressive abnormal lysosomal glycogen storage in muscle tissue leads to impaired muscle function and to degeneration of muscle fibers. Children and adults with PD present with limb-girdle muscular weakness, diaphragm weakness and impaired breathing ability. Further, patients with classic infantile PD suffer from hypertrophic cardiomyopathy. To date, the muscle pathology and the extent of the disease can be assessed using invasive techniques (e.g., muscle biopsies) or imaging (e.g., MRI). These techniques are time consuming, and especially in young patients, require anesthesia, which increases the acute risk of respiratory failure.

Multispectral optoacoustic tomography (MSOT) allows the detection of specific endogenous chromophores like collagen, myoglobin or hemoglobin by using a non-invasive approach comparable to conventional ultrasound. Instead of sound waves, MSOT illuminates tissue with near-infrared light of transient energy, which is absorbed and results in thermo-elastic expansion of certain molecules. This expansion generates ultrasound waves that are detected by the same device. Multispectral illumination and unmixing then allows the precise localisation and quantification of muscle-specific subcellular structures. MSOT has already been demonstrated the potential to visualize the muscular structure and the clinical extent of muscular disease in patients with Duchenne muscle dystrophy and differentiates those patients from healthy volunteers.

The aim of the study is to establish glycogen as a novel PD-specific imaging target using MSOT-imaging. Furthermore, we will apply MSOT-imaging to identify a PD-specific muscle pathology-signature by quantification of already established targets (collagen, myoglobin, hemoglobin, glycogen if applicable). This signature will aid in differentiating PD from other muscular pathologies and healthy volunteers and will ultimately serve as a potential non-invasive monitoring biomarker.

Content is confidential.

4. Project summary (German)

Bei Patienten mit Morbus Pompe (PD) führt eine fortschreitende abnorme lysosomale Glykogenspeicherung im Muskelgewebe zu einer Beeinträchtigung der Muskelfunktion und zur Degeneration von Muskelfasern. Kinder und Erwachsene mit Morbus Pompe zeigen eine Schwäche der Gliedergürtelmuskulatur, eine Zwerchfellschwäche und eine Beeinträchtigung der Atemfunktion. Außerdem leiden Patienten mit klassischer infantiler PD an einer hypertrophen Kardiomyopathie. Bislang können die Muskelpathologie und das Ausmaß der Erkrankung mit invasiven Techniken (z. B. Muskelbiopsien) oder bildgebenden Verfahren (z. B. MRT) beurteilt werden. Diese Techniken sind zeitaufwendig und erfordern insbesondere bei jungen Patienten eine Anästhesie, was das akute Risiko eines Atemversagens erhöht.

Die multispektrale optoakustische Tomographie (MSOT) ermöglicht den Nachweis spezifischer körpereigener Chromophore wie Kollagen, Myoglobin oder Hämoglobin durch einen nicht-invasiven Ansatz, der mit konventionellem Ultraschall vergleichbar ist. Anstelle von Schallwellen beleuchtet MSOT das Gewebe mit Nahinfrarotlicht von transienter Energie, das absorbiert wird und zu einer thermoelastischen Ausdehnung bestimmter Moleküle führt. Diese Ausdehnung erzeugt Ultraschallwellen, die von demselben Gerät detektiert werden. Die multispektrale Beleuchtung und Entmischung ermöglichen dann die präzise Lokalisierung und Quantifizierung von muskelspezifischen subzellulären Strukturen. MSOT hat bereits das Potential gezeigt, die Muskelstruktur und das klinische Ausmaß der Muskelerkrankung bei Patienten mit Duchenne-Muskeldystrophie sichtbar zu machen und diese Patienten von gesunden Freiwilligen zu unterscheiden.

Ziel der Studie ist es, Glykogen als neuartiges PD-spezifisches Bildgebungsziel mittels MSOT-Bildgebung zu etablieren. Darüber hinaus werden wir MSOT anwenden, um eine PD-spezifische Muskelpathologie-Signatur durch Quantifizierung bereits etablierter Targets (Kollagen, Myoglobin, Hämoglobin, Glykogen, falls zutreffend) zu identifizieren. Diese Signatur wird helfen, PD von anderen muskulären Pathologien und gesunden Probanden zu unterscheiden und wird letztendlich möglicherweise als potenzieller nicht-invasiver Überwachungs-Biomarker dienen.

Content is confidential.

5. Responsabilities

Study director

Dr. Ferdinand Knieling

Pediatric Experimental and Translational Imaging Laboratory (PETI_Lab)

Department of Pediatrics
University Hospital Erlangen
Loschgestr. 15
91054 Erlangen
Germany
Tel. 09131 8533118
Mail: ferdinand.knieling@uk-erlangen.de

Prof. Dr. Regina Trollmann

Head of the Department of Neuropediatrics

Department of Pediatrics
University Hospital Erlangen
Loschgestr. 15
91054 Erlangen
Germany
Tel.: 09131 85 33753
Mail: regina.trollmann@uk-erlangen.de

Sponsoring

Investigator-initated trial, funding by Sanofi Genzyme

6. Scientific background

Pompe disease (PD) is a rare, autosomal-recessive disorder caused by deficiency of the lysosomal acid alpha-glucosidase enzyme (GAA), leading to generalized build-up of glycogen, especially in the heart, muscle, liver and nervous system (Pompe, 1932, Hers, 1963). Among the glycogen storage diseases, PD is the only one with a defect in lysosomal metabolism.

PD is considered as a progressive disease with variation by age of onset, severity of organ involvement and degree of myopathy. This great phenotypic variability has led to the creation of types based on the age of onset and degree of organ involvement. They all have in common, that symptoms of affected patients are expected to worsen over time if left untreated. The classification is generally based on the age of onset as infantile (infantile onset Pompe disease, IOPD) when it presents during the first 12 months of life and late-onset (LOPD) when first symptoms appear after 12 months of age. If cardiomyopathy is present, IODP is generally referred to as classic Pompe disease (however there may be variably classification in the literature with the infantile or childhood forms). Clinically, infants with classic PD present during the first few months of life with rapidly progressive disease characterized by prominent hypertrophic cardiomyopathy, hepatomegaly, hypotonia, generalized muscle weakness, macroglossia, feeding difficulties and respiratory insufficiency. Mortality rate is high by one year of age if untreated (Kishnani 2004, 2006). Patients with non-classic PD will usually present within the first year of life with motor developmental delay and weakness, but without clinically relevant cardiac involvement. The rate of clinical progression is slower in these children and without treatment, death will usually occur in childhood as a result of respiratory insufficiency (Hers 1963, Slonim 2000). LODP include childhood and adult-onset PD. These patients generally present with slowly progressive limb girdle type weakness and respiratory insufficiency without significant cardiomyopathy (Cupler 2012, Preisler 2013). The diagnosis of PD is usually established by the typical clinical presentation, followed by confirmation of GAA deficiency in dried blood spots, e.g. through new-born screening (Bodamer, 2017). Further (confirmatory) methods include GAA activity measurement in lymphocytes, muscle or skin fibroblasts, as well as GAA mutation testing (Van den Hout, 2003, Case, 2012). All of them are invasive techniques. Early identification is important as it will likely significantly improve the outcome for all patients with PD as treatment can be initiated earlier. Treating the underlying cause of PD involves the replacement of the missing enzyme GAA via enzyme replacement therapy (ERT) with alglucosidase alfa (recombinant human GAA, rhGAA). Currently, this is the only specific treatment approved for PD. In classical IOPD, treatment significantly lengthens survival and improves motor development and respiratory and cardiac function (Kishnani, 2007). The sooner ERT begins, the better are the results (Chien, 2013). With ERT being one

Content is confidential.

very important aspect of care, patients will also need a multidisciplinary approach to ensure that all aspects of the disease are addressed.

Regardless of age of onset and severity, all patients with PD should be monitored prospectively (Kishnani, 2006, Bembi, 2008). However, there is lack of standardization across centers. A variety of clinical evaluations and tests are currently used for monitoring Pompe's disease, which may include laboratory tests including CK, AST, ALT, and LDH, cardiologic tests including electrocardiogram and echocardiogram and respiratory tests including sleep studies and breathing tests to measure lung capacity. To quantify muscle involvement electromyography is an option as well as clinical tests including 6 minutes walking test or timed to up and go test. Muscle MRI of affected patients often show fatty degeneration of muscles. One study showed that muscle MRI correlates with muscle function in patients with adult-onset Pompe disease (Figueroa-Bonaparte, 2016). Another study suggested that muscle imaging data in late-onset Pompe disease reveal a correlation between the pre-existing degree of lipomatous muscle alterations and the efficacy of long-term enzyme replacement therapy (Gruhn, 2015). For small children, however, there is always a need for sedation for MRI's, limiting its use. Therefore, ultrasound is another option to examine children's muscles.

At the moment there are no prospective biomarkers available to detect muscle degeneration at an early age and/or to follow up disease progression or ERT-treated patients. Within the last years our multidisciplinary research team (Medical Department 1, Department of Pediatrics and Adolescent Medicine, University Hospital Erlangen) published a novel non-invasive imaging modality to be able to detect subcellular tissue composition in vivo. Multi-spectral optoacoustic tomography (MSOT), an imaging technology comparable to ultrasound, allows quantitative imaging in patients of all ages (including the non-sedated child).

Content is confidential.

For MSOT, similar to a conventional ultrasound, an ultrasound transducer is positioned on the patient's skin. Instead of sound waves, MSOT illuminates tissue with light of transient energy, typically near-infrared laser light pulses, which are absorbed by the tissue, resulting in thermo-elastic expansion. This expansion gives rise to ultrasound waves that are detected by the same device. Studies have already shown that MSOT-based assessment of hemoglobin levels in the intestinal wall has the potential for Assessment of Crohn's Disease Activity (Knieling 2017, Waldner 2016). With a newly configurated device (Acuity Echo, iThera Medical GmbH, Munich, custom-built platform) an extended spectrum of laser light can be used, allowing the detection of not only hemoglobin but also further biomarkers like collagen or lipid. In our recent study, we were able to show the molecular composition of muscles in Duchenne muscle dystrophy via MSOT suggesting non-invasive measured collagen content as a novel biomarker for disease severity (Regensburger, 2019). In this study we want to establish glycogen as a novel PD-specific imaging target using MSOT-imaging.

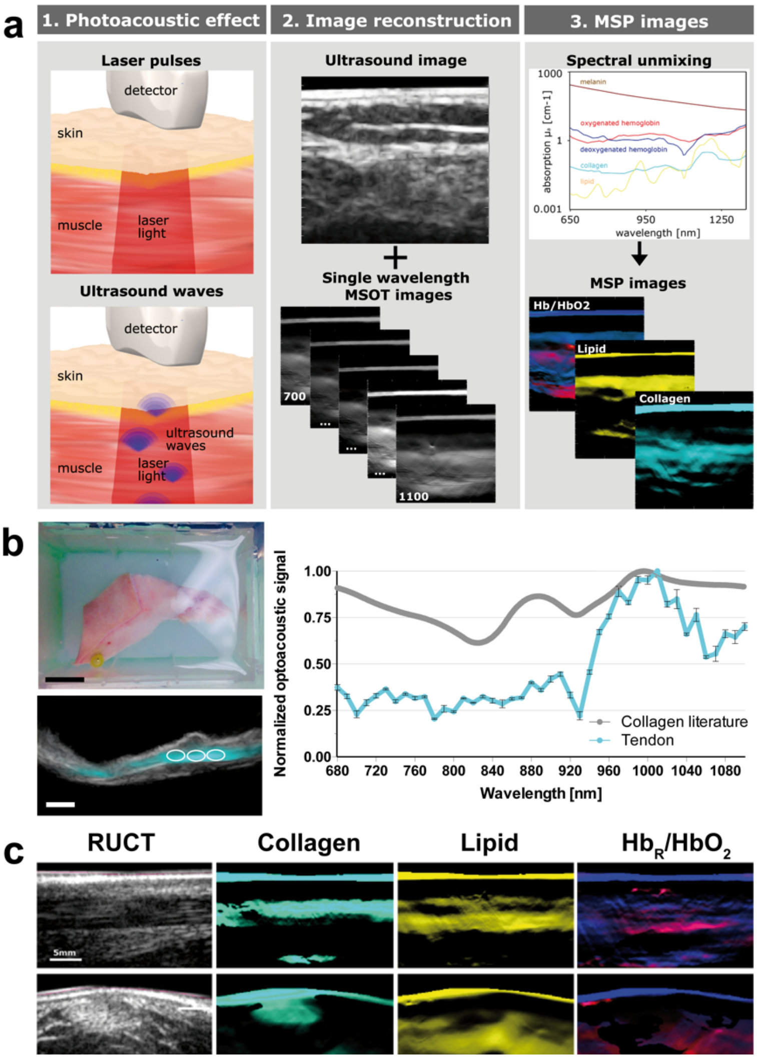

Figure 1 - MSOT principle (a), experimental preliminary work (b), and first time representation of collagen in vivo (c)

Content is confidential.

Figures 1 and 2 show exemplary MSOT images. In the light of the progression and the sometimes severe course of PD, this method would for the first time allow a non-invasive and quantitative determination of the molecular composition of muscle tissue. In this first pilot study in patients with PD, it will now be investigated whether the differences in the muscle composition of healthy volunteers and PD patients can be quantified and whether this could simultaneously be used as marker during ERT-therapy. In the future, this could generate a completely new, non-invasive method to evaluate endogenous biomarkers for therapy response.

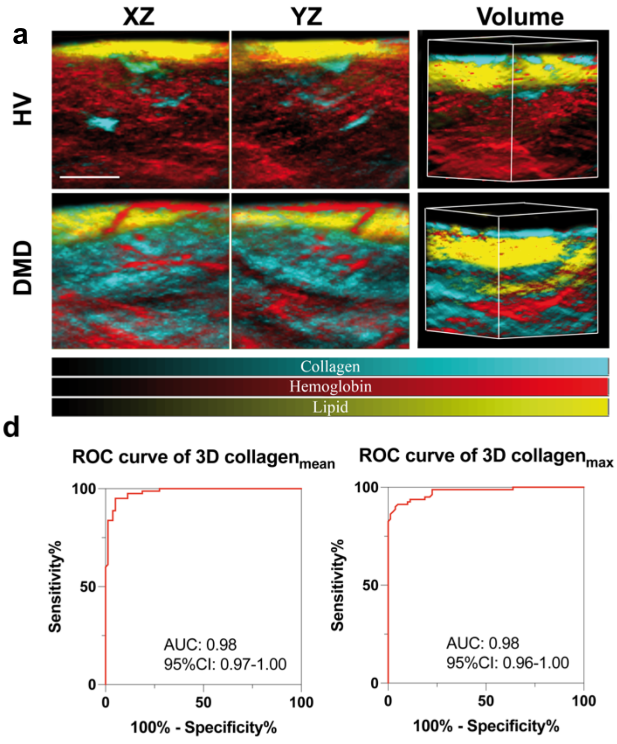

Figure 2 - Feasibility of 3D MSOT image (a) and diagnostic quality (b).

Literature

Pompe JC. Over idiopatische hypertrophie van het hart. Ned Tijdschr Geneeskd. 1932; 76; 304-12.

Hers HG. Alpha-Glucsidase deficiency in generalized glycogen storage diease (Pompe's disease), Biochem J. 1963, 86, 11-6.

Kishnani PS, Howell RR. Pompe disease in infants and children. J Pediatr. 2004;144(Suppl 5):S35-43.

Kishnani PS, Hwu WL, Mandel H, Nicolino M, Yong F, Corzo D. A retrospective, multinational, multicenter study on the natural history of infantile-onset Pompe disease. J Pediatr. 2006;148(5): 671-6.

Hers HG. Alpha-Glucosidase deficiency in generalized glycogen storage disease (Pompe's disease) Biochem J. 1963;86:11–16.

Slonim AE, Bulone L, Ritz S, Goldberg T, et al. Identification of two subtypes of infantile acid maltase deficiency. J Pediatr. 2000;137:283–285.

Cupler EJ, Consensus treatment recommendations for late-onset Pompe disease. Muscle Nerve. 2012, 45:319-33.

Preisler N. Late-onset Pompe disease is prevalent in unclassified limb-girdl muscular dystrophies. Mol Genet Metab. 2013, 110:287-9.

Bodamer OA. Newborn Screening for Pompe Disease. Pediatrics, 2017. Jul; 140 (Suppl 1) S4-S13

Van den Hout HM. The natural course of infantile Pompe's disease: 20 original cases compared with 133 cases from the literature. Pediatrics. 2003, 112.332-40.
Case LE. Infantile Pompe disease on ERT. Update on clinical presentation, musculoskeletal management and exercise considerations. Am J Med Genet C Semin Med Genet. 2012. 160C: 69-79.

Content is confidential.

Lumyzyme (alglucosidase alfa) for injection, for intravenous use (prescribing information). Cambridge, MA: Genzyme Corporation; 2014.

Myozyme (alglucosidase alfa) for injection, for intravenous use (prescribing information). Cambridge, MA: Genzyme Corporation; 2014.

Kishani PS, Recombinant human Recombinant human acid [alpha]-glucosidase: major clinical benefits in infantile-onset Pompe disease. Neurology, 2007, Jan 9; 68 82); 99-109.

Chien YH, Hwu WL, Lee NC. Pompe disease: early diagnosis and early treatment make a difference. Pediatr Neonatol. 2013;54(4):219–227

Kishnani PS, Steiner RD, Bali D,et al. Pompe disease diagnosis and management guideline [published correction appears in Genet Med. 2006;8(6):382]. Genet Med. 2006;8(5):267–288

Bembi B, Cerini E, Danesino C,et al. Management and treatment of glycogenosis type II. Neurology. 2008;71(23 suppl 2):S12–S36

Figueroa-Bonaparte S, Muscle MRI Findings in Childhood/Adult Onset Pompe Disease Correlate with Muscle Function, PLoS One. 2016; 11(10): e0163493.

Gruhn KM, Muscle imaging data in late-onset Pompe disease reveal a correlation between the pre-existing degree of lipomatous muscle alterations and the efficacy of long-term enzyme replacement therapy. Mol Genet Metab Rep. 2015 Jun; 3: 58–64.

Knieling F. Multispectral Optoacoustic Tomography for Assessment of Crohn's Disease Activity. N Engl J Med. 2017 Mar 30;376(13):1292-1294.

Knieling F. Light and sound - emerging imaging techniques for inflammatory bowel disease. World J Gastroenterol. 2016 Jul 7;22(25):5642-54.

Waldner MJ, Knieling F. et al Multispectral Optoacoustic Tomography in Crohn's Disease: Noninvasive Imaging of Disease Activity. Gastroenterology. 2016 Aug;151(2):238-40.

Regensburger A. et al. Detection of collagens by multispectral optoacoustic tomography as an imaging biomarker for Duchenne muscular dystrophy. Nat Med. 2019 Dec 11 14:9809-9821.

Content is confidential.

7. Study objective

Primary study objective:

Comparison of the optoacoustic spectrum determined by MSOT in patients with PD compared to healthy volunteers, generating a new biomarker for disease monitoring in PD.

Secondary study objectives:

- Comparison of the quantitative glycogen signal fraction determined by MSOT in patients with PD compared to healthy volunteers
- Comparison of the quantitative lipid signal fraction determined by MSOT in patients with PD compared to healthy volunteers
- Comparison of the quantitative fraction of collagen signal determined by MSOT in patients with PD compared to healthy volunteers
- Comparison of the quantitative fraction of hemo-/myoglobin signal determined by MSOT in patients with PD compared to healthy volunteers
- Comparison of the quantitative fraction of oxygenated/deoxygenated hemoglobin determined by MSOT in patients with PD compared to healthy volunteers

- Correlation of glycogen content determined with MSOT with disease duration/patient age
- Correlation of lipid content determined with MSOT with disease duration/patient age
- Correlation of collagen determined by MSOT with disease duration/patient age
- Correlation of haemoglobin/myoglobin content determined by MSOT with duration of disease/patient age
- Correlation of oxygenated/deoxygenated hemoglobin determined by MSOT with duration of disease / patient age

- Correlation of glycogen content determined with MSOT with R-Pact scale
- Correlation of lipid content determined with MSOT with R-PAct scale
- Correlation of collagen determined by MSOT with R-PAct scale
- Correlation of haemoglobin/myoglobin content determined by MSOT with R-PAct scale
- Correlation of oxygenated/deoxygenated hemoglobin determined by MSOT with R-PAct scale

- Correlation of glycogen content determined with MSOT with age-related functional muscle tests (Hammersmith Infant Neurological Examination (HINE)/The Children's

Hospital of Philadelphia Infant Test of Neuromuscular Disorders (CHOP Intend)/expanded Hammersmith functional motor scale (HFMSE)/ Revised Upper Limb Module (RULM)/6-Minute-Walk Test (6-MWT)/Time-to-go-up-and-go-test/MRC Muscle Strength Grades)

- Correlation of lipid determined with MSOT with age-dependent functional muscle tests (RULM /6-MWT/Time-to-get-up-and-go/MRC)
- Correlation of collagen determined with MSOT with age-dependent functional muscle tests (RULM/6-MWT/Time-to-get-up-and-go/MRC)
- Correlation of hemo-/myoglobin content determined with MSOT with age-dependent functional muscle tests (RULM/6-MWT/Time-to-get-up-and-go/MRC)
- Correlation of oxygenated/deoxygenated hemoglobin determined with MSOT with age-related functional muscle tests (RULM /6-MWT/Time-to-get-up-and-go/MRC)

- Correlation of glycogen content determined with MSOT with B-mode ultrasound (Heckmatt scale/Echogenity/Gray Scale Level/UGAP)
- Correlation of lipid determined with MSOT with B-mode ultrasound (Heckmatt scale/Echogenity/Gray Scale Level/UGAPI)
- Correlation of collagen determined with B-mode ultrasound (Heckmatt scale/Echogenity/Gray Scale Level/UGAP)
- Correlation of hemo-/myoglobin content determined with MSOT with B-mode ultrasound (Heckmatt scale/Echogenity/Gray Scale Level/UGAP)
- Correlation of oxygenated/deoxygenated hemoglobin determined with B-mode ultrasound (Heckmatt scale/Echogenity/Gray Scale Level/UGAP)

- Correlation of glycogen content determined with MSOT with respiratory function tests (Spirometry)
- Correlation of lipid content determined with MSOT with respiratory function tests (Spirometry)
- Correlation of collagen determined by MSOT with respiratory function tests (Spirometry)
- Correlation of haemoglobin/myoglobin content determined by MSOT with respiratory function tests (Spirometry)
- Correlation of oxygenated/deoxygenated hemoglobin determined by MSOT with respiratory function tests (Spirometry)

- Measurement of signal differences in right / left comparison

Content is confidential.

- Correlation of glycogen content determined with MSOT with (functional) magnetic resonance imaging parameters
- Correlation of lipid determined with MSOT with (functional) magnetic resonance imaging parameters
- Correlation of collagen determined with (functional) magnetic resonance imaging parameters
- Correlation of hemo-/myoglobin content determined with (functional) magnetic resonance imaging parameters
- Correlation of oxygenated/deoxygenated hemoglobin determined with (functional) magnetic resonance imaging parameters

Hypotheses:

- The optoacoustic spectrum of muscles of patients with PD is different compared to healthy volunteers
- The optoacoustic spectrum of the liver of patients with PD is different compared to healthy volunteers
- The quantitative fraction of glycogen signal in muscles determined by MSOT differs in patients with PD compared to healthy volunteers
- The quantitative fraction of lipid signal in muscles determined by MSOT differs in patients with PD compared to healthy volunteers
- The quantitative fraction of collagen signal in muscles determined by MSOT differs in patients with PD compared to healthy volunteers
- The quantitative fraction of hemo-/myoglobin in muscles determined by MSOT differs in patients with PD compared to healthy volunteers
- The quantitative fraction of oxygenated/deoxygenated hemoglobin in muscles determined by MSOT differs in patients with PD compared to healthy volunteers
- There is a correlation between MSOT derived glycogen signal and clinical status of patients with PD
- There are no side differences in patients with PD and healthy volunteers

Study type

Since no data exists so far to support the hypothesis of this study, it is an explorative study / pilot study.

Content is confidential.

8. Endpoint parameters

All measurements with MSOT are performed over the paraspinal musculature, Musculus trapezius as well as proximal and distal limb muscles in a right-left comparison (leg proximal: Musculus quadriceps, distal: Musculus triceps surae; arm proximal: Musculus biceps, distal: Forearm flexors) in healthy subjects compared to patients with PD.
Additionally, we will perform measurements of the liver with MSOT.

Primary endpoint:
Optoacoustic Absorption Spectrum of Muscle and liver in PD.
This target is measured non-invasively by MSOT.

Secondary endpoints:
Quantitative glycogen signal (in arbitrary units)
Quantitative lipid signal (in arbitrary units)
Quantitative collagen signal (in arbitrary units)
Quantitative hemo/myoglobin signal (in arbitrary units)
Muscle oxygenation (in %)
These target values are measured non-invasively by MSOT.

B-mode ultrasound
- Heckmatt scale
- Echogenitiy
- Gray Scale Level
- Ultrasound-Guided Attentuation Parameter (UGAP)

R-PaAt scale
- Summed raw score

Respiratory function test
- FVC
- FEV1

Functional magnetic resonance imaging of lung.
- Ventilation Defects / Non-defected
- Perfusion Defects / Non-defected
- Combined Defects / Non-defected

Content is confidential.

Magnetic resonance imaging of biceps muscle.

Correlation of glycogen detected by MSOT with clinical scores for determining muscle strength consisting of:

- Revised Upper Limb Module (RULM)
- MRC Muscle Strength Grades

in case of ability to walk additionally:

- 6-minute walking test
- Time-to-get-up-and-go-test

These target values are clinically determined at presentation (electronic patient record, physician letters):

Age

Sex

Weight

Skin color

Ethnic Background

Disease duration

Current medication

Results of last biopsy (if available)

Results of last MRI (if available)

Content is confidential.

Study Parameters	PD	HV
MSOT<br>-'Glycogen'<br>-Lipid<br>-Collagen<br>-Hemoglobin oxygenated<br>-Hemoglobin deoxygenated	X<br>X<br>X<br>X<br>X<br>X	X<br>X<br>X<br>X<br>X<br>X
Ultrasound<br>-Heckmatt<br>-Echogenity<br>-Muscle texture<br>-Grey scale level<br>-UGAP	X<br>X<br>X<br>X<br>X<br>X	X<br>X<br>X<br>X<br>X<br>X
Magnetic resonance imaging<br>-Ventilation defects<br>-Perfusion defects<br>-Combined defects<br>-Biceps muscle imaging	X<br>X<br>X<br>X<br>x	X<br>X<br>X<br>X
R-Pact scale (≥16y)	X	X
Respiratory function test<br>-FVC<br>-FEV1	X<br>X<br>X	X<br>X<br>X
Muscle tests<br>-RULM<br>-6-MWT<br>-Time-to-get-up-and-go<br>-MRC	X<br>X<br>X<br>X<br>X	X<br>X<br>X<br>X<br>X

Patient History		
Confirmed Disease	X<br>Type:<br>Crim:	X
Diagnostics:<br>-Genetics<br>-Dried Blood Spot<br>-Urinary Glc 4<br>-Lymphozytes	X<br>X<br>X<br>X<br>X	
Muscle biopsy	X	
Neurological tests:<br>-EMG<br>-MRI<br>-Sleep Study	X<br>X<br>X<br>X	
Lab values<br>-CK<br>-Liver enzymes	X<br>X<br>X	

Content is confidential.

Sitting ability<br>Walking ability	X<br>X	
Ventilation	X	
Medication		

9. Study design

Monocentric/Multicentric

This is a multicentric study with matched collectives (age, gender)/

Study arms: intervention/control

Interventions are not planned. A comparison is made between patients with PD and healthy volunteers. The study procedure is identical for PD patients and healthy volunteers. Healthy volunteers are used as controls (no muscular pathology, no excessive intramuscular lipids/collagen, no excessive glykogen) versus PD patients (muscular pathology, intramuscular lipids/collagen, excessive glycogen).

Randomization

Randomization is not planned. The allocation to the groups is based on the known diagnosis (PD, other muscular pathology) and controls (subjects).

Blinding

Blinding for the examination is not possible due to the possible clear clinical appearance. Blinding takes place during the measurement and and evaluation of the data. Blinding of the patients/controls is not necessary.

Content is confidential.

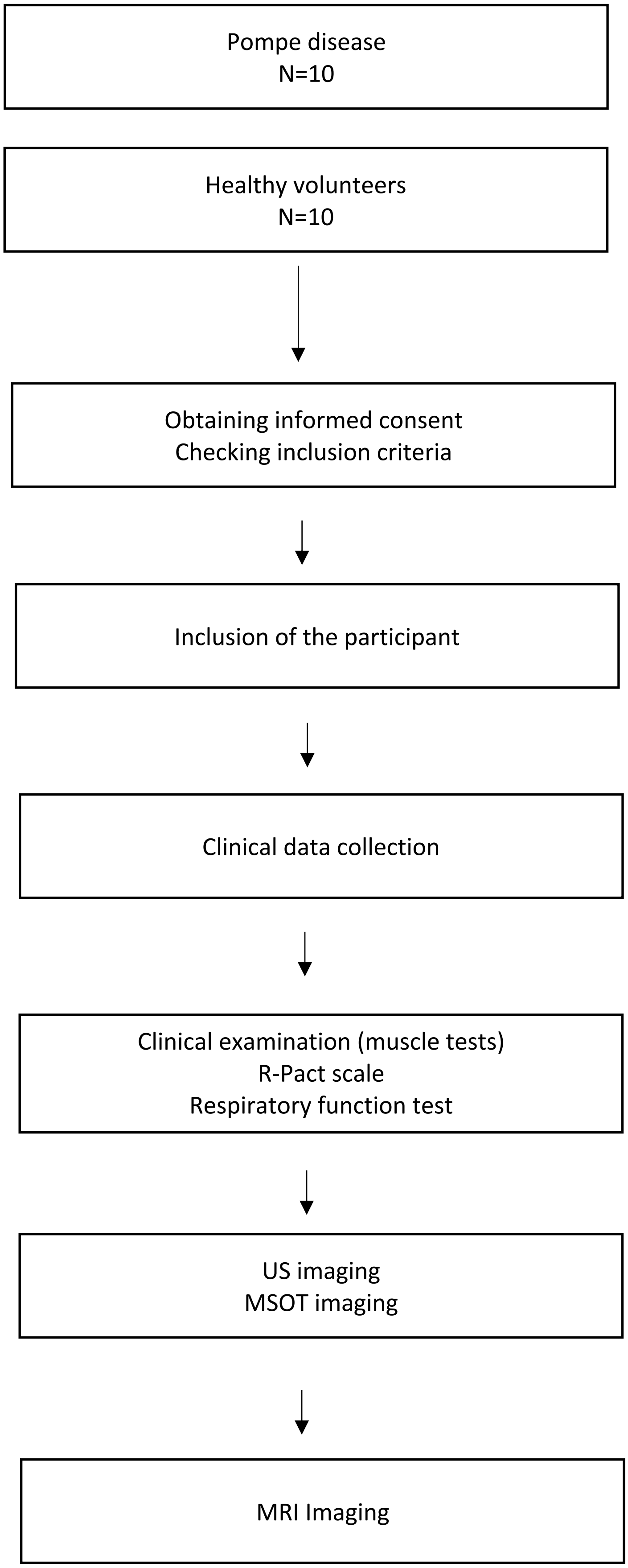

Content is confidential.

Study population

Inclusion and exclusion criteria

Inclusion criteria:
PD patients (IOPD and LOPD)

- Confirmed diagnosis of Pompe disease
- From 18 years of Age
- Independent from current therapy

Healthy controls

- From 18 years of Age, matched (age, gender) to PD collective

Exclusion criteria

PD patients

- Pregnancy
- Tattoo on skin to be examined

Healthy controls

- Anamnestic of other signs of myopathy or liver disease
- Pregnancy
- Tattoo on skin to be examined

Patient/control number

As this is a pilot study, an exact case number calculation is not possible. It is planned to study a total of 10 patients with PD and 10 healthy controls.

Recruitment routes and measures

Patients (and parents) are informed about the possibility of participating in the study in the context of an elective presentation at the Clinic for Pediatrics and Adolescent Medicine (Neuropediatrics) and the Clinic for Neurology at the University Hospital in Erlangen, Germany as well as the the Clinic for Pediatrics and Adolescent Medicine (Neuropediatrics) in Gießen, Germany. Additional recruitment options include the Clinic Rummelsberg, Schwarzenbruck, Germany, the German Society for muscular diseases (DGM), the treatNMD network and the international Pompe Association. If the patient is willing to participate, he/she will be fully

Content is confidential.

informed about the aims and methods (especially about the scientific/explorative character of the study), benefit and risk and revocability of the study participation. Patients in childhood and adolescence will also be informed and educated about the study and its procedure according to their age.

Healthy volunteers are recruited in the outpatient departments of the Clinic for Neurology at the University Hospital in Erlangen, Germany.

Acutely ill or unstable patients are not recruited. In the preliminary phase, volunteers are parallelised with the PD collective in terms of age and sex.

10. Study flow

Procedure for informing about and obtaining consent

Patients or test persons can only be included in the study after a written consent has been given. The written declaration of consent requires oral and written information of the patients/test persons as well as their parents or legal guardians about goals and methods (incl. scientific-explorative character of the study), benefit and risk as well as revocation of participation in the study. Children and adolescents are informed by means of age-appropriate, comprehensible patient information sheets. By giving their written consent, the patients/test persons and their parents/guardians declare that they agree to the collection and storage of study-relevant data and their verification by monitoring or authorities. The study participant must be clearly informed that the declaration of consent can be withdrawn at any time and without any disadvantage. Furthermore, all study participants/test persons and parents/guardians are informed that this study is a purely scientific study without any current diagnostic or therapeutic benefit.

The original of the declaration of consent will be kept in the study folder at the place of study. The patient/control and the parents/guardian receive a copy of the patient information and declaration of consent. The patient information and the consent form are attached to this protocol.

Measurements

After informing the patient / control and parent / guardian and obtaining consent, clinical scores are collected to assess muscle strength. R-Pact scale will be collected and respiratory function tests will be performed.

Subsequently, MSOT imaging is performed on 6 anatomical regions in all study participants: Paraspinal muscles, Musculus trapezius, upper/lower arm and upper/lower leg on predefined

Content is confidential.

muscle groups. (Leg proximal: Musculus quandriceps, distal: Musculus triceps surae; arm proximal: Musculus biceps, distal: Forearm flexors). The examination is analogous to sonography over the corresponding skin layers without further invasive procedures. The anatomical region can be localized by means of built-in B-image sonography; the corresponding optoacoustic signals can then be conducted. The duration per anatomical region is limited to 5 minutes; this corresponds to a maximum of 10 minutes for the parasinal muscles and Musculus trapezius, a maximum of 20 minutes for both upper extremities and 20 minutes for both lower extremities and 10 minutes for the examination of the liver. Patients can remain in a relaxed posture during the examination, without the need for breathing maneuvers or similar assistance.

At last, patients undergo MRI imaging of the lung and biceps muscle. Participants can be in a relaxed position during the examination. The time allotted for MRI is 30 minutes.

Recording of target parameters

- Determination of routine data (duration of disease, current medication)
- Clinical evaluation of muscle strength by 6-MWT, Time-to-get-up-and-go, MRC muscle test
- Assessment of R-Pact scale
- Performance of respiratory function test
- Non-invasive in-vivo measurement of glycogen, lipid, collagen, myo/hemoglobin content and oxygenation by MSOT

Content is confidential.

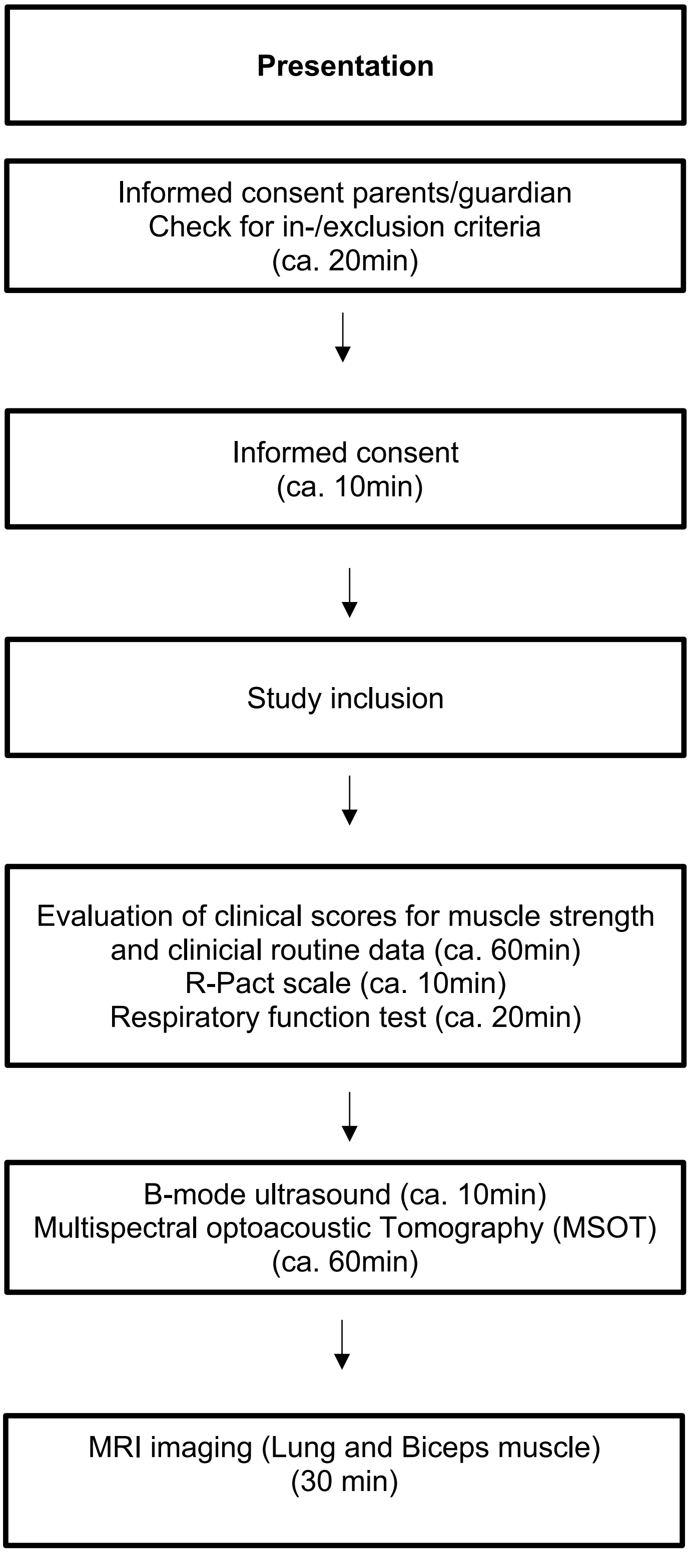

Time schedule and study duration for the individual patient/control

For the individual patient, the duration of the study participation is 220 minutes. Approximately 30 minutes are spent on education for study participants and parents/guardians, 60 minutes on clinical (routine) testing of muscle strength, 10 minutes on R-Pact scale, 20min on

respiratory function test, 10 minutes for B-mode ultrasound and 60 minutes for the MSOT examination. For MRI imaging 30 minutes are required.

Total duration of study

Depending on the number of patients, the expected total duration of the study up to the inclusion of the last patient is 8 months.

11. Benefit-risk-assessment

All study-related risks

Based on the classification criteria for medical devices (Directive 93/42/EEC, Annex IX), the iThera Medical optoacoustic system complies with Class IIa laser systems:

- Active diagnostic device
- Non-invasive
- Temporary use (<60 min)

The used system has CE-certification (TÜV Süd, 02.05.2021, type designation according to imprint: MSOT Acuity Echo). A conformity assessment procedure to expand the use of the system is not intended or planned by the manufacturer at this time. It is therefore a purely scientific pilot study. There is no dependency relationship with the manufacturer; all diagnostic and analytical procedures are available to the study directors on site. The cooperation with the company will be regulated in a separate contract drawn up by the legal department before the start of the study.

Compliance with energy limits

The laser safety and maximum permitted radiation dose for irradiation with laser pulses is regulated in the ANSI and IEC 60825 laser standards. The MSOT system complies with these standards and remains below the MPE (maximum permissible exposure) limits for skin irradiation and is therefore considered safe.

Temperature increases in tissue

Optoacoustic imaging does not result in a significant increase in tissue temperature. The absorption of a laser pulse in tissue results in a local transient temperature increase of a few millikelvin. Depending on the duration of the examination and the patient's skin type, there is typically a temperature increase in the range of less than one degree Kelvin.

Histological changes in tissue

Content is confidential.

Histological changes in the target tissue and surrounding structures are neither expected nor have they been observed in previous preclinical and clinical studies.

Slight, reversible redness or warming is only to be expected in sensitive skin.

Such impairment of the patient can be noticed by the test person or physician at any time; the examination can then be interrupted or discontinued. In any case, no irreversible damage is to be expected.

In principle, the near-infrared light used in the MSOT can lead to retinal damage if the eye is irradiated. To prevent this, the participants and examiners will wear appropriate laser safety glasses during the examination.

Since the data obtained will not be used to interpret findings, there is no risk of possible misdiagnosis or incorrect display of data in this exploratory pilot study.

There are no other risks within the scope of this study, nor have they been described based on our own preliminary data.

Magnetic resonance imaging

Unlike computed tomography, MRI does not use ionizing radiation, so no permanent side effects are to be expected.

More than 1 million MRI examinations at higher field strengths (1.5/3T) are performed annually in Germany. Provided that the general contraindications for MRI examinations are observed, no serious side effects occur. MRI is therefore one of the safest examination procedures.

The risks associated with an MRI examination emanate from the three main components of the MRI system.

Static magnetic field

The static magnetic field exerts forces and torques on ferromagnetic objects that can be so strong that the (mostly ferromagnetic) objects fly uncontrollably toward the magnet and can hit patients and staff (missile effect). The magnetic forces are proportional to the field strength B and the field change with location (dB/dz). These risks are lower with the low-field MRI system. Risks are further minimized by providing safety training to operators and excluding patients with ferromagnetic implants from the study.

The gradient system

Content is confidential.

Gradient switching can cause the appearance of magnetic phosphenes and nerve and muscle cell stimulation. Rapidly switched gradients produce high magnetic field changes per time (dB/dt) that induce voltages in the body. If a current flows through the tissue via nerve endings, for example, this can result in so-called peripheral nerve stimulation. However, the manufacturer of the gradient system guarantees compliance with the limits for gradient switching times and amplitudes recommended in the IEC 60601-2-33 guidelines. Thus, nerve stimulation effects need not be considered further in the risk assessment of this study.

Another safety-relevant effect of gradient fields is noise caused by gradient switching due to current- and field-strength-dependent Lorentz forces in the gradient tube. These unpleasant loud knocking noises occur especially during fast imaging processes where high currents flow through the gradients. Noise levels can rise up to 115dB for 1.5T tomographs (background noise: approx. 78dB). Due to the lower magnetic forces at 0.55T, we expect lower noise levels. In addition, patients always wear hearing protection during the examination, so that the noise exposure remains well below the legal limit of 99 dB.

The high-frequency system

During the MR measurement, radiofrequency (RF) fields are sent into the human body, which are partially absorbed by the tissue and can lead to an increase in body temperature. The thermoregulatory response of human tissue to RF pulses has now been studied for 50 years. For example, using conservation of energy, it has been calculated that the body temperature of lightly clothed patients with undisturbed thermoregulation at room temperature increases by up to 0.6 °C with RF exposure of 4 W/kg (63 MHz, 1.5 Tesla). The assumed specific absorption rate (SAR) of 4 W/kg body tissue corresponds to the so-called "controlled mode first level" (IEC safety guideline), which is also used as an upper limit in routine clinical imaging. The magnitude of the actual temperature rise is generally smaller because skin cooling was not considered in the calculations (worst case scenario).

The body's energy production at rest is about 1.2 W/kg - equivalent to the energy conserved when wearing a thin sweater. Most healthy people are capable of compensating for 15 times this resting energy, and only a minimal increase in core body temperature occurs. Studies at 1.5 T have shown that RF absorption in humans leads only to the expected cardiac adaptation and does not cause adverse health effects. Theoretically, a 63 kg person is even capable of emitting 1296 W to the environment through the skin by cardiac adaptation (i.e., maximum increase in blood flow) - this would correspond to a SAR of 20.6 W/kg.

The same limits are observed with the 0.55 Tesla MRI system. At 0.55 Tesla, the wavelength of the radio waves used is significantly longer, so that the spatial distribution of the energy emission is more homogeneous and thus the risks tend to be lower.

Content is confidential.

12. Biometrics

Exploratory study: Explanation of the statistical methodology, justification of the chosen case number

Case number calculation

As this is a pilot study and no information is available on the expected differences between the different groups, no case number calculation was carried out. The number of cases given represents an estimate or is within reasonable limits for a pilot study.

Statistical Methodology

Continuous variables are given as mean values with standard deviation, categorical variables as numbers with percentages where appropriate. The MSOT parameters are compared using a two-sided, unpaired t-test with equal deviations. If the standard deviation is not equal, Welch correction is applied where appropriate. ROC (Receiver Operator Characteristics) analyses between healthy and sick persons are also planned. Genetics serves as the gold standard. Correlations are indicated by the Pearson coefficient. All statistical tests will be double-sided and a p-value of <0.05 is considered statistically significant. All analyses are performed with GraphPad Prism (version 7.00 or later, GraphPad Software, La Jolla, CA, USA), RStudio (version 1.1.456 or later, RStudio Inc., Boston, MA, USA) or IBM SPSS Statistics (version 24 or later, IBM Corp., Armonk, NY, USA).

13. Data management and protection

All raw data, such as patient records, represent source documents. Their availability is ensured for routine monitoring. The participation of the individual patients or test persons in the study is documented; the study director maintains a separate list to identify the all screened and participating patients. This list contained the names and date of birth as well as the examination date and pseudonymization abbreviations of the patients and test persons. The study director is responsible for the quality of data collection and storage. The data storage (total data) is carried out on computers or specially designed network drives of the University Hospital Erlangen. The raw imaging data (no patient-related data) is stored on specially designated servers of the company iThera Medical GmbH.

Pseudonymisation

Prior to a scientific analysis of the materials and data of this study, all information will be pseudonymised in accordance with the guidelines of the Federal Data Protection Act.

Data transfer

Content is confidential.

Data sharing in this study is intended solely for the MSOT raw data. The company iThera Medical GmbH will work with this data to ensure adequate recording quality and to develop algorithms for evaluation. The data will only be transferred pseudonymized on encrypted physical drives. The data will not be used for a later approval of the prototype used. The cooperation will be explicitly regulated by the legal department before the study starts (a supplementary agreement to the service contract can be found in the appendix).

The study results can be published anonymously, but it will not be possible to draw conclusions about the identity of the participating persons. The data will be kept for 10 years and destroyed afterwards.

Revocation, data deletion

If the declaration of consent is revoked, data collected up to this point can be taken into account. The patient has the right to demand their destruction, provided that legal provisions do not conflict with the destruction.

14. Biomaterial handling

No biomaterials are obtained.

15. Individual paticipant insurance

The participants of the study are insured through the group contract of the CCS Erlangen.

Content is confidential.

16. Signatures

Dr. med. Ferdinand Knieling
Study director

Prof. Dr. med. Regina Trollmann
Study director

Prof. Dr. med. Joachim Wölfle
Clinic director

Content is confidential.
